# Myocardial composition and contractile function of right atrial trabeculae from type 2 diabetic and nondiabetic male patients

**DOI:** 10.14814/phy2.70509

**Published:** 2025-08-11

**Authors:** Liam Tianlang Zhang, Amelia Sally Power, Nicholas Kang, Marie‐Louise Ward

**Affiliations:** ^1^ Department of Physiology, Faculty of Medical and Health Sciences University of Auckland Auckland New Zealand; ^2^ Green Lane Cardiothoracic Surgical Unit Auckland City Hospital Auckland New Zealand

**Keywords:** cardiomyocyte, diabetes, fibroblast, human right atrial trabeculae, type I collagen, type III collagen

## Abstract

Diabetes impairs myocardial function. This study investigates tissue composition and contractile function of isolated atrial tissue from type 2 diabetic patients prior to heart failure. Multicellular trabeculae were dissected from freshly obtained right atrial appendage samples from consenting patients undergoing heart surgery. Trabeculae were mounted in a stress transducer at optimal length and electrically stimulated to contract. The steady‐state force produced in response to stimulation at physiological frequencies was recorded at 37°C. Myocardial composition of trabeculae from the same patient samples was examined by immunolabeling of contractile proteins, extracellular collagens (types I and III), and fibroblasts. Relative to nondiabetic, diabetic trabeculae had increased diastolic (*p* = 0.01) and decreased active stress (*p* = 0.02), with no difference in the time course of contraction and relaxation. Immunohistological findings showed that diabetic trabeculae had reduced myofilament content (*p* = 0.02), whereas the relative abundance of collagens type I and type III, cardiomyocytes, and fibroblasts was comparable to nondiabetic trabeculae. However, diabetic trabeculae had a significant reduction in the collagen I/III ratio (*p* = 0.04) with differences in fibroblast morphology. Our study demonstrated that the contractile function at this stage of diabetic heart disease was associated with small changes in myocardial composition in the right atrium.

## 1 BACKGROUND

Type 2 diabetes mellitus (T2D) is a potent modifiable risk factor for diabetic heart disease (Rubler et al., 1972). Approximately 14% of T2D patients develop heart disease independent of vascular disease and hypertension (Shah et al., 2015); evidence shows that T2D negatively impacts the contractile function of the human heart (Celentano et al., 1995). Since human heart failure was first linked to diabetes mellitus (Leyden, 1881), our understanding of the mechanisms that lead to structural and functional alterations of the diabetic heart remains limited. The increased prevalence of T2D contributes to a growing global health burden (Zhou et al., 2024), emphasizing the need for a better understanding of the pathophysiology of diabetes‐driven cardiomyopathy.

Cardiomyocytes are the force‐generating cells of the heart surrounded by myocardial extracellular matrix (ECM). While some studies suggest that contractile dysfunction of the diabetic heart originates from the cardiomyocytes (Heerebeek et al., 2008; Jones et al., 2023; Montaigne et al., 2014; Zhang et al., 2008), others have also shown that diabetes‐driven myocardial compositional remodeling and fibrosis contribute to the impaired contraction and relaxation of the diabetic heart (Cohen et al., 2021; Mizushige et al., 2000; Ng et al., 2012; Sakakibara et al., 2011; Zhang et al., 2008). However, the myocardial ECM has not been well characterized in the human right atrium, which is more readily available for study in isolation than human ventricles. Thus, little is known about how compositional and fibrotic remodeling alters right atrial function during the different stages of diabetic cardiomyopathy. To address the knowledge gap, this study compares the myocardial composition, extent of fibrosis, and contractility of right atrial tissue from T2D patients undergoing routine heart surgery with age‐matched nondiabetic (ND) controls.

Therefore, our study had the following aims: (i) to determine the relative abundance and localisation of type I and type III collagen, the most abundant ECM proteins which regulate the myocardial mechanical properties; (ii) to quantify cardiomyocyte contractile protein per tissue area; (iii) to determine the abundance of collagen‐producing fibroblasts; and (iv) to compare tissue composition with the contractile function of right atrial tissue isolated from the same patient tissue samples. To achieve our aims, we obtained small samples of freshly excised right atrial appendage (RAA) tissue from consenting patients undergoing coronary artery bypass graft surgery, with and without T2D. This study aimed to provide new knowledge of the relationship between myocardial composition and contractile function under the influence of T2D in the human right atrium.

## 2 METHODS

For detailed methods, see Appendix S1.

### 2.1 Human right atrial appendage tissue samples

A piece of RAA tissue (~0.5 × 2 cm) was obtained from the border of the cannula incision site of patients undergoing coronary artery bypass graft surgery, as previously described (Jones et al., 2023). Tissue was immediately placed in modified Krebs–Henseleit (KH) buffer solution (118 mM NaCl, 4.75 mM KCl, 1.18 mM MgSO_4_.7H_2_O, 1.18 mM KH_2_PO_4_, 24.8 mM NaHCO_3_, 11 mM of glucose, 0.25 mM CaCl_2_, and 25 mM 2,3‐butanedione monoxime (BDM)) continuously bubbled with carbogen (95% O_2_ and 5% CO_2_) to maintain pH at 7.4. Tissue was then transported within minutes to our research laboratory at the University of Auckland. On arrival, the tissue was placed in a fresh buffer solution, and trabeculae micro‐dissected from the endocardial surface.

Samples for this study were selected from male patients of similar age, body mass index (BMI), and without prior history of cardiac surgery. Recruited patients were allocated into ND and T2D groups based on previous diagnoses of T2D, with T2D patients having a glycated hemoglobin (HbA1c) level greater than 40 mmol mol^−1^.

### 2.2 Microdissection of trabeculae

Two multicellular trabeculae were microdissected free from each patient sample. One, with a diameter of 300 μm or less, was used for measurement of contractile function, and the other was fixed and stored at −80°C for immunohistological examination of myocardial composition.

#### 2.2.1 Functional trabeculae measurements

Following microdissection, trabeculae were transferred to a temperature‐controlled muscle chamber (model 801C, Aurora Scientific, Canada) on the stage of an inverted microscope (Nikon TE2000‐U, Nikon Instruments, Japan) and continuously superfused with modified KH buffer bubbled with carbogen. Trabeculae were mounted between platinum stimulating electrodes, with one end of the trabecula attached to a hook connected to a 3D micromanipulator, and the other end held in a wire hook extending from a calibrated force transducer (AE801, Kronex, Oakland, CA, USA). The circulating buffer was then replaced with a BDM‐free KH buffer, and the [Ca^2+^] increased to 1.5 mM. Once the temperature of the muscle bath was steady at 37°C, trabeculae were stimulated at 0.5 Hz with a 5 ms pulse. The stimulus voltage was increased until a force response was obtained (Radnoti REDSTIM multichannel stimulator, ADInstruments, New Zealand). The final stimulus voltage was then set at 20% above the threshold voltage. Once the force development of trabeculae reached steady‐state, the stimulation frequency was adjusted to 1 Hz. Trabeculae were then incrementally lengthened until the active force produced no longer increased with increasing length (optimal length; L_o_).

Isometric force was acquired using LabChart (version 8.1.30, AD Instruments, New Zealand). For each trabecula, the contractile force was converted to stress (mN mm^−2^) by normalizing to cross‐sectional area. This was estimated using the inverted microscope at low magnification with trabeculae at optimal length. Consecutive steady‐state twitches at each stimulation frequency were selected and analyzed using the LabChart “peak analysis” plugin. Contractile data were averaged over the 10 twitches and exported for statistical analysis. Diastolic stress was measured 50 ms before the stimulus was applied.

#### 2.2.2 Preparation of trabeculae for immunohistochemistry

**FIGURE 1:**
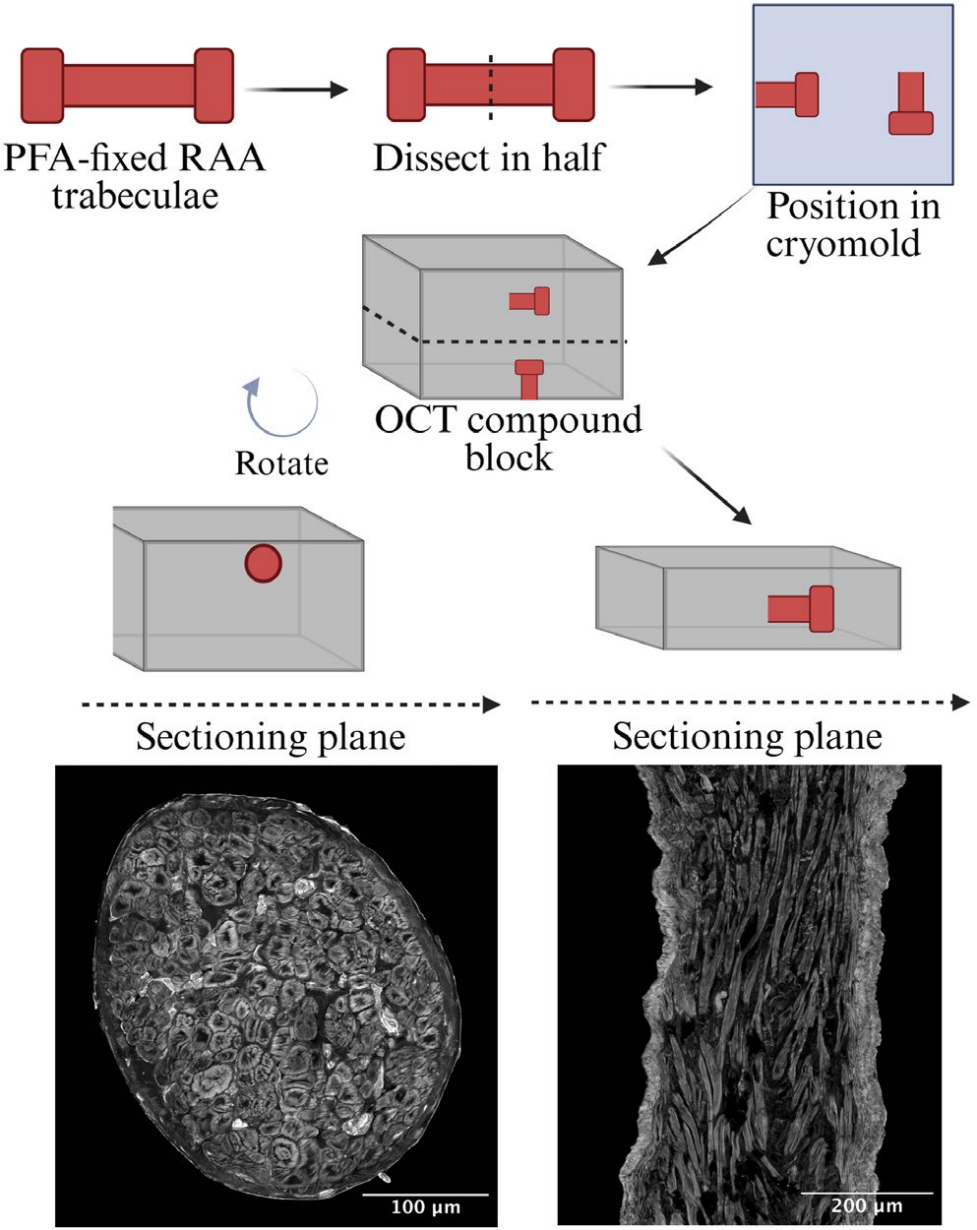
Trabeculae fixation and cryo‐sectioning. A schematic diagram showing the processing of a human right atrial appendage (RAA) trabecula during fixation and cryo‐sectioning. First, the paraformaldehyde (PFA) fixed trabecula was cut in half, and the two halves were positioned in the cryomold perpendicular to each other. Optimal cutting temperature (OCT) compound was added to the cryomold and frozen. The OCT compound block was divided into two, each containing half a trabecula, and positioned in the cryostat as shown. This enabled the acquisition of both longitudinal and transverse sections from each trabecula.

Once dissected free, trabeculae remained in dissection buffer while the blocks of tissue at either end were pinned to the base of a sylgard lined dish to ensure cardiomyocytes were longitudinally aligned and of similar sarcomere length. Once pinned in place, K‐H buffer was removed and the addition of 2% paraformaldehyde in phosphate‐buffered saline (PBS) at room temperature for 20 min. Fixed trabeculae were then sequentially dehydrated in 10%, 20%, and 30% sucrose in PBS overnight at each concentration. Dehydrated trabeculae were microdissected into two equal portions and embedded with optimal cutting temperature (OCT) compound (Sakura Finetek, USA) in cryomolds (Tissue Tek® Sakura Finetek) in orientations perpendicular to each other (Figure 1). Trabeculae positioned in the cryomolds were frozen in isopentane, cooled by liquid nitrogen, and stored at −80°C.

Figure 1 illustrates a stepwise process of cryo‐sectioning each trabecula into 10 μm sections for subsequent immunolabeling. OCT compound blocks were separated into two and positioned on the cryostat (CM3050S, Leica, Wetzlar, Germany) with cardiomyocytes being either in longitudinal or transverse orientation (Figure 1). This allowed the acquisition of highly comparable tissue sections between samples in terms of cellular orientation. Five adjacent 10 μm sections were obtained from each trabecula in both sectioning planes. Acquired sections were immediately mounted on poly‐L‐lysine coated coverslips (22 × 50 mm, #1.5, Thermo Fisher Scientific, USA).

#### 2.2.3 Immunohistochemistry

Antibodies used in immunohistochemistry and immunolabeling protocols are detailed in Table S1a,b. Validation of antibodies and fluorescent probes was carried out in paraformaldehyde‐fixed RAA wall tissue. No‐primary antibody controls and negative controls were used to test the binding specificity and reactivity of antibodies and fluorescent probes toward human heart tissue (Figure S2a).

Longitudinal sections of trabeculae were labeled for type I collagen, type III collagen, and myofilaments (F‐actin). Transverse sections of trabeculae were labeled for type I collagen, myofilaments, vimentin (fibroblasts), and nuclei. Types I and III collagen and vimentin were labeled in trabeculae sections using rabbit antitype I collagen antibody (transverse sections: 1:50, longitudinal sections: 1:100, Abcam Cat# ab34710, RRID:AB_731684), mouse anti‐type III collagen antibody (1:400, Abcam Cat# ab6310, RRID:AB_305413), and Alexa‐Fluor 647 conjugated mouse anti‐vimentin antibody (1:400, Thermo Fisher Scientific Cat# MA5‐11883‐A647, RRID:AB_2662891), respectively. Sections labeled by primary antibodies were then incubated with anti‐rabbit Alexa Fluor 488 (1:100, Thermo Fisher Scientific Cat# A‐11008, RRID:AB_143165) and anti‐mouse Alexa Fluor 647 antibodies (1:100, Thermo Fisher Scientific Cat# A‐21235, RRID:AB_2535804). Contractile proteins within cardiomyocytes were labeled with Alexa Fluor 594‐conjugated phalloidin (1:100, Thermo Fisher Scientific Cat# A12381, RRID:AB_2315633), and cell nuclei were labeled with 4',6‐diamidino‐2‐phenylindole (DAPI) (Thermo Fisher Scientific Cat# P36935), respectively.

For primary antibody labeling, trabeculae sections were first rehydrated in PBS and subsequently blocked with normal goat serum (Thermo Fisher Scientific Cat# 10000C) and Image‐iT® FX Signal Enhancer (Thermo Fisher Scientific Cat# I36933) each for 1 h. Blocked sections were washed in PBS and incubated in a “cocktail” of diluted primary antibodies prepared in 50 μL tissue incubation solution (1% bovine serum albumin, 0.05% sodium azide, and 0.05% Triton X‐100 PBS) at 4°C overnight. Primary antibody‐labeled sections were washed with PBS and then incubated in a “cocktail” of secondary antibodies and fluorescent probes prepared in 50 μL of tissue incubation solution for 2 h at room temperature. Labeled sections were subjected to a final wash in PBS, blotted dry, and mounted on slides using Prolong Gold antifade reagent containing DAPI. Slides were left to cure at room temperature for at least 48 h in the dark before being used for confocal imaging.

#### 2.2.4 Immunohistological analysis

Confocal imaging was performed using a Zeiss LSM 800 Airyscan confocal microscope (Zeiss, Oberkochen, Germany) in the Biomedical Imaging Research Unit (BIRU) at the University of Auckland. Zeiss 40x oil objective (NA 1.3) and 63× oil objective (NA 1.4) lenses (Zeiss, Oberkochen, Germany) were used to image trabeculae in longitudinal and transverse sections.

The central area of the tissue (i.e., myocardium) was captured using the “Tile‐scan” function with ZEN (Zeiss, Oberkochen, Germany). Acquired images were assembled in ZEN and analyzed using ImageJ Fiji (RRID:SCR_003070) (Schindelin et al., 2012). Images were transformed into “TIFF” format, and the techniques of global and local thresholding were used accordingly to segment the area of labelling. The relative abundance of myofilaments in trabeculae sections was determined by the area of phalloidin labelling, with the abundance of type I collagen, type III collagen, and vimentin determined by the area of corresponding antibody label relative to the tissue area examined. Custom‐written image analysis pipelines were developed in ImageJ Fiji to quantify cardiomyocyte and fibroblast cell populations, tissue density, and morphology from trabeculae transverse sections.

#### 2.2.5 Statistical analysis

All data are presented as mean ± one standard deviation (SD) unless specified otherwise. This study adopted a nested sampling design when investigating differences in myocardial composition of antibody labels between ND and T2D trabeculae. Five replicated measurements (tissue sections) were taken from each sample (trabecula) in both longitudinal and transverse orientation. Nested *t*‐tests were performed in GraphPad Prism 10 (version 10.1.2, GraphPad Software, Boston, USA) to test whether there was a significant difference in means between ND and T2D groups when considering within‐sample correlation. A mixed model analysis was performed in Statistical Analysis System (version 9.4, SAS Institute, North Carolina, USA) to test the association between different measurements in consideration of within‐sample correlation.

Contractile function data was compared between groups using two‐way ANOVA to examine the group effect of T2D and stimulation frequency. Pearson's correlation analysis was carried out to investigate the association between histological and functional data. Two‐way ANOVA and Pearson's correlation were performed in GraphPad Prism 10. Statistical significance was determined as a probability (*p*) equal to or less than 0.05, with actual *p* values provided in tables and figures.

## 3 RESULTS

### 3.1 Patient clinical data

The clinical characteristics of patients contributing tissue for this study are summarized in Table 1. All patients recruited had coronary artery disease. No significant differences between ND and T2D patient groups were observed in BMI (ND: 30 ± 4 kg m^−2^; T2D: 29 ± 3 kg m^−2^, *p* = 0.56), age (ND: 66 ± 11 years; T2D: 62 ± 11 years, *p* = 0.50), gender, and heart medications. Echocardiographic data showed both groups had normal left ventricular systolic and diastolic parameters, apart from one ND patient who had a mildly reduced left ventricular systolic function and one T2D patient with moderate left ventricular diastolic dysfunction. There was no significant difference in LVEF between the groups (ND: 56 ± 6%; T2D: 61 ± 3%, *p* = 0.06). Most patients had LVEF within the normal range, with the exception of one ND patient who had a mildly depressed LVEF of ~47%. This patient, along with two other patients from the T2D group, also had mildly dilated right atria.

**TABLE 1:** Patient characteristics and diabetes medications.

Groups	ND (*n* = 7)	T2D (*n* = 7)	*p* Value
Age (years)	66 ± 11	62 ± 11	0.50
Gender	Male	Male	–
Clinical characteristics			
BMI (kg m^−2^)	30 ± 4	29 ± 3	0.56
Ejection fraction (%)	56 ± 6	61 ± 3	0.06
Presurgical arrhythmias	1/7	2/7	0.98
HbA1c (mmol mol^−1^)	37 ± 6	60 ± 13	0.001
Smoker	2/7	5/7	0.63
Hypertension	3/7	5/7	0.88
Hyperlipidemia	6/7	5/7	0.98
Diabetes medications			
Metformin	0/7	4/7	0.23
SGLT2 inhibitors	0/7	1/7	0.90
GLP1 receptor agonists	0/7	1/7	0.90
DPPIV inhibitors	0/7	2/7	0.67
Sulfonylureas	0/7	1/7	0.90
Insulin	0/7	5/7	0.10

*Note*: Table 1 data are shown as mean ± SD, or as a fraction of the sample size. Statistical significance was determined by Student's *t*‐test, with significance of ratios determined by chi‐squared tests and *p* ≤ 0.05.

### 3.2 Trabeculae composition

**FIGURE 2:**
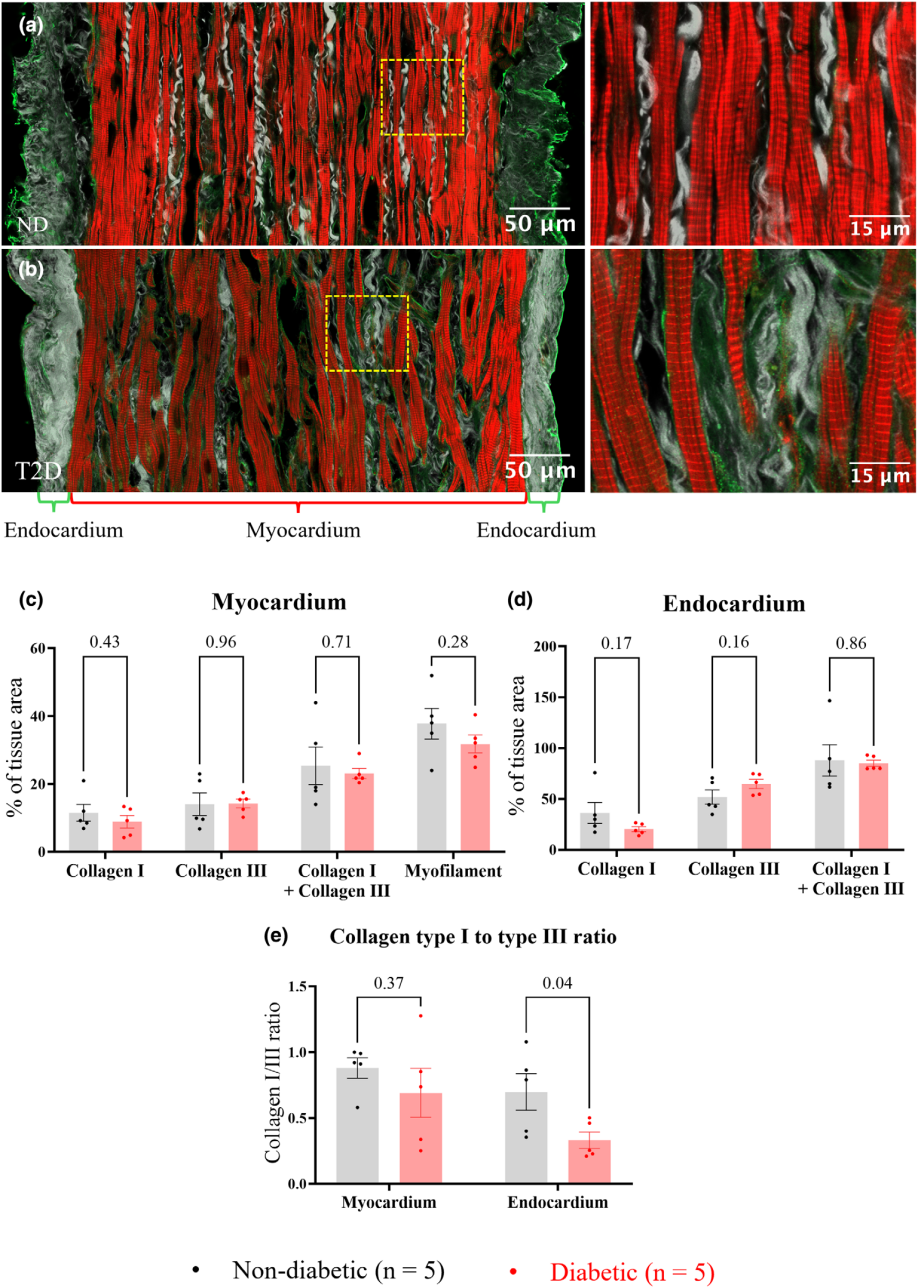
Comparison of right atrial appendage (RAA) trabeculae myocardium and endocardium composition from longitudinal sections. Panels (a) and (b) show representative RAA trabeculae from the ND and T2D patient groups, with RHS panels showing the area inside the yellow lines at higher magnification. Tissue sections were labeled with type I collagen‐antibodies (green), type III collagen‐antibodies (gray), and the marker of myofilament F‐actin, phalloidin (red). Panels (c) and (d) show the percentage area of trabeculae myocardium and endocardium occupied by the labeled proteins. Panel (e) shows the collagen type I/III ratio. Data are presented as mean ± SEM, with significance determined by nested t‐tests; *p* values are shown above the bar graphs. Significant differences between groups were indicated by *p* ≤ 0.05.

Myocardial composition was examined in RAA trabeculae from 5 ND and 5 T2D patients. Trabeculae dissected from the endocardial surface of the RAA tissue were of varying sizes. Figure 2a,b show the central portion of representative ND and T2D trabeculae (i.e., the myocardium) used for immunohistochemistry of longitudinal sections. The internal myocardium (rich in cardiomyocytes) was surrounded by an endocardium that was predominantly type I and type III collagen. Measurement of trabeculae diameter (ND: 484 ± 151 μm; T2D: 490 ± 111 μm, *p* = 0.94), myocardium diameter (ND: 450 ± 156 μm; T2D: 432 ± 115 μm, *p* = 0.84), endocardium thickness (ND: 25 ± 18 μm; T2D: 34 ± 8 μm, *p* = 0.33), sarcomere length (ND: 2.0 ± 0.3 μm; T2D: 2.2 ± 0.1 μm, *p* = 0.24), and aspect ratio (major/minor diameters, ND: 1.4 ± 0.2 μm; T2D: 1.5 ± 0.3 μm, *p* = 0.65) showed that trabeculae used for histology were not significantly different in dimensions between groups. Trabeculae of smaller cross‐sectional areas were used for force measurements to ensure that diffusion of metabolites to the core was not compromised. These trabeculae were also of comparable diameter between groups (ND: 263 ± 69 μm; T2D: 192 ± 49 μm, *p* = 0.12).

#### 3.2.1 Relative abundance of myofilaments and extracellular proteins

Since trabeculae contain distinct myocardial (inner) and endocardial (outer) regions, the composition of these two areas was separately quantified for each of the labeled trabeculae. Longitudinal trabeculae sections showed that the following relative abundances for the myocardium were not different between groups (Figure 2c): collagen type I (ND: 11.5 ± 5.6%; T2D: 8.2 ± 4.2%, *p* = 0.43), collagen type III (ND: 14.1 ± 7.4%; T2D: 14.3 ± 2.9%, *p* = 0.96), combined collagens type I and type III (ND: 25.4 ± 12.4%; T2D: 23.2 + 3.4%, *p* = 0.71), and myofilaments (ND: 37.8 ± 10.1%; T2D: 27.9 ± 5.9%, *p* = 0.28). In comparison to ND, longitudinal sections of T2D trabeculae endocardium also showed no difference in content (Figure 2d): type I collagen (ND: 36.2 ± 23.1%; T2D: 20.4 ± 5.4%, *p* = 0.17), type III collagen (ND: 51.8 ± 15.7%; T2D: 64.7 ± 10.3%, *p* = 0.16), and combined type I and type III collagens (ND: 88.0 ± 34.6%; T2D: 85.1 ± 6.8%, *p* = 0.86). However, the ratio of collagen type I to collagen type III (collagen I/III) in the endocardium of T2D trabeculae was significantly reduced compared to ND (ND: 0.70 ± 0.31; T2D: 0.33 ± 0.14, *p* = 0.04); although there was no difference between groups in the myocardial collagen I/III ratio (ND: 0.88 ± 0.17; T2D: 0.69 ± 0.42, *p* = 0.37, Figure 2e).

**FIGURE 3:**
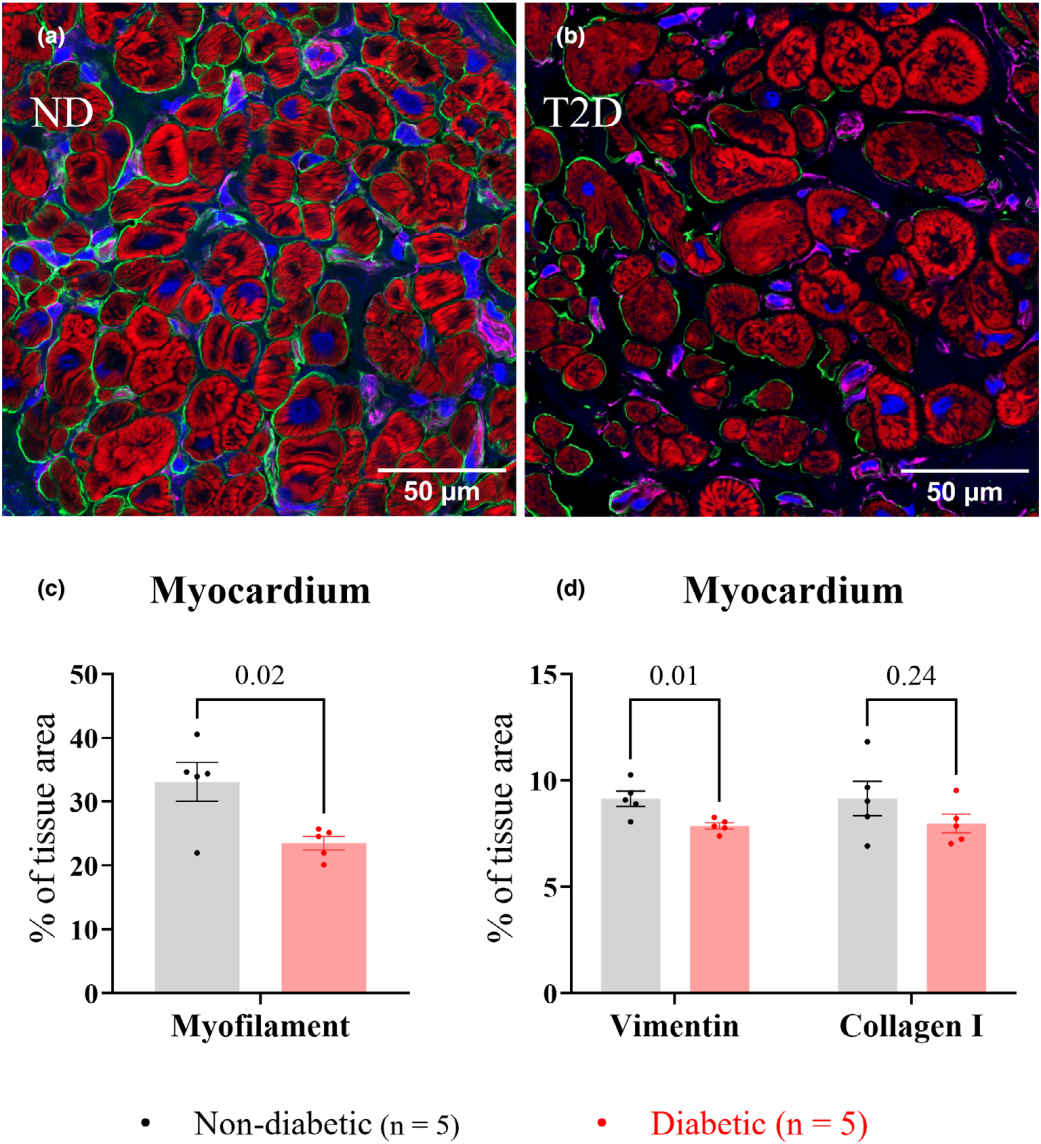
Comparison of myocardium composition of right atrial appendage (RAA) trabeculae. Panels (a) and (b) show representative RAA trabeculae sections from the ND and T2D patient groups with the area inside the yellow lines shown below at higher magnification. Transverse sections were labeled with the myofilament‐marker phalloidin (red), type I collagen antibodies (green), vimentin antibodies (purple), and the nuclei‐marker DAPI (blue). Panel (c) shows the percentage area of trabeculae sections occupied by the myofilament labeling. Panel (d) shows the percentage area of trabeculae sections occupied by vimentin and type I collagen labeling. Data are presented as mean ± SEM, with significance determined by nested t‐tests, with *p* values shown above the bar graphs. *p* ≤ 0.05 indicates a significant difference between groups.

Figure 3 shows histological analysis carried out on trabeculae transverse sections. Relative to ND, T2D trabeculae showed a significant decrease in tissue area labeled by phalloidin, a marker of myofilament F‐actin (ND: 33.1 ± 6.8%; T2D: 23.5 ± 2.4%, *p* = 0.02, Figure 3c), and vimentin antibody (ND: 9.3 ± 0.8%; T2D: 7.9 ± 0.3%, *p* = 0.01, Figure 3d), whereas no significant difference was observed between groups in the tissue area labeled by type I collagen antibody (ND: 9.2 ± 1.8%; T2D: 8.0 ± 1.0%, *p* = 0.24, Figure 3d).

#### 3.2.2 Cardiomyocytes and fibroblasts

The number of cardiomyocytes and fibroblasts was quantified for each transverse trabeculae section and normalized to the total number of cells quantified from DAPI labelling in the same section. Cardiomyocytes and fibroblasts constituted 17.6 ± 1.6% and 51.6 ± 5.4% of the total number of cells in the ND trabeculae and 18.8 ± 6.7% and 45.3 ± 5.5% in the T2D trabeculae, respectively. They were not significantly different between the ND and T2D groups (cardiomyocytes, *p* = 0.69; fibroblasts, *p* = 0.11, Figure S2b).

**FIGURE 4:**
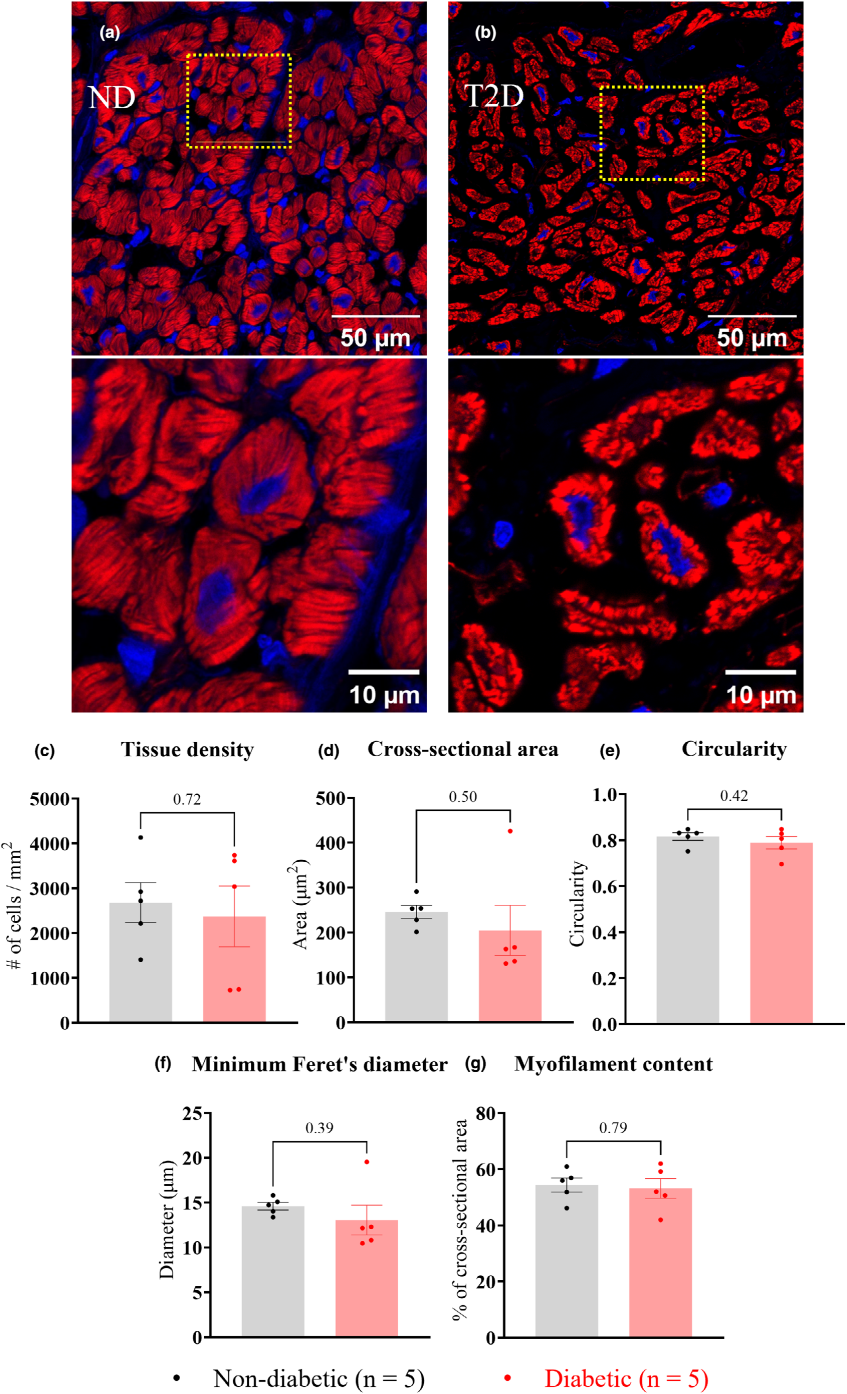
Cardiomyocyte content of right atrial appendage (RAA) trabeculae. Panels (a) and (b) show transverse sections of representative RAA trabeculae from ND (*n* = 5) and T2D (*n* = 5) patients, with areas inside the yellow lines shown below each panel at higher magnification. Cardiomyocytes were labeled with the myofilament‐marker phalloidin (red) and nuclei‐marker DAPI (blue). Manual counting of cardiomyocyte numbers was carried out following identification by phalloidin stain for *n* = 5 images per trabecula, with *N* = 5 trabeculae per group. Panels (c)–(g) show comparisons of cardiomyocyte parameters following analysis in Image J Fiji for: cardiomyocyte number mm^−2^ (c), cross‐sectional area (d), circularity (e), minimum Feret's diameter (f), and myofilament content (g). Data are presented as mean ± SEM with *p* values shown above the bar graphs. Significance was determined by nested t‐tests with *p* ≤ 0.05 indicating a significant difference between groups.

Cardiomyocytes and fibroblasts were then examined in the central myocardium using custom‐written analysis pipelines in Image J Fiji to compare tissue density and morphology between groups. In comparison to ND, cardiomyocytes in the T2D trabeculae had a comparable tissue density (ND: 2681 ± 1000 per mm^2^; T2D: 2374 ± 1515 per mm^2^, *p* = 0.72, Figure 4c), cross‐sectional area (ND: 245 ± 33 μm^2^; T2D: 205 ± 125 μm^2^, *p* = 0.50, Figure 4d), circularity (ND: 0.82 ± 0.02; T2D: 0.79 ± 0.03, *p* = 0.42, Figure 4e), minimum Feret's diameter (distance between two parallel lines tangential to the cell boundary) (ND: 14.6 ± 0.9 μm; T2D: 13.1 ± 3.7 μm, *p* = 0.39, Figure 4f), and myofilament content as ND (ND: 54.4 ± 5.6%; T2D: 53.2% ± 7.9%, *p* = 0.79, Figure 4g).

**FIGURE 5:**
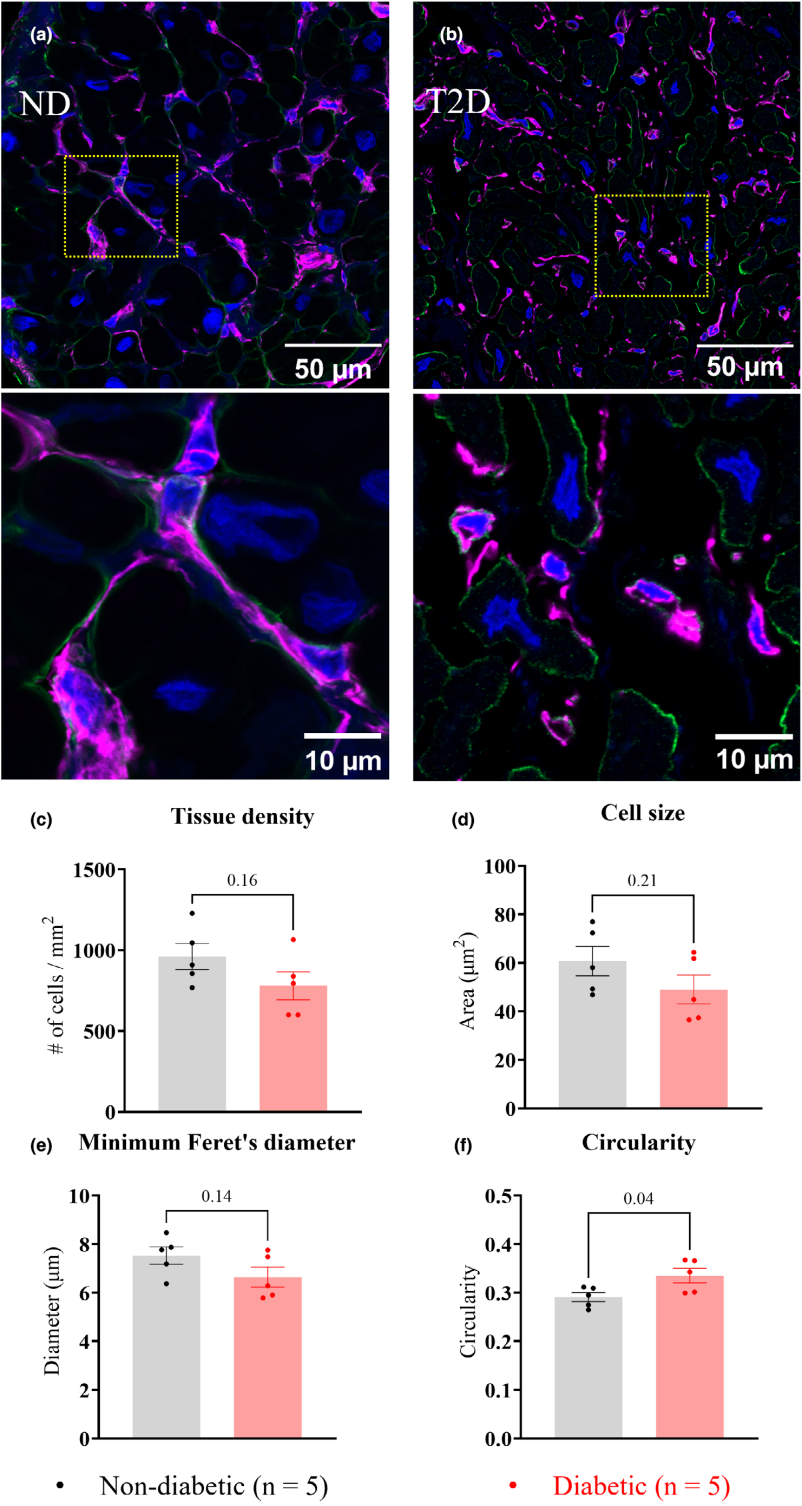
Fibroblast labeling of right atrial appendage (RAA) trabeculae. Panels (a) and (b) show transverse sections from representative trabeculae labeled to show fibroblasts and type I collagen from ND (*n* = 5) and T2D (*n* = 5) patients, with areas inside the yellow lines shown below each panel at higher magnification. Sections were labeled with type I collagen‐antibody (green), vimentin‐antibody (purple), and nuclei‐marker DAPI (blue). Panels (c)–(f) show comparisons of fibroblast parameters following analysis in Image J Fiji for: Number of fibroblasts mm^−2^ (c); fibroblast cross‐sectional area (f); minimum Feret's diameter (e); and circularity (f) between groups. Data are presented as mean ± SEM, with *p* values shown above the bar graphs. Significance was determined by nested *t*‐tests, with *p* ≤ 0.05 indicating a significant difference between groups.

**FIGURE 6:**
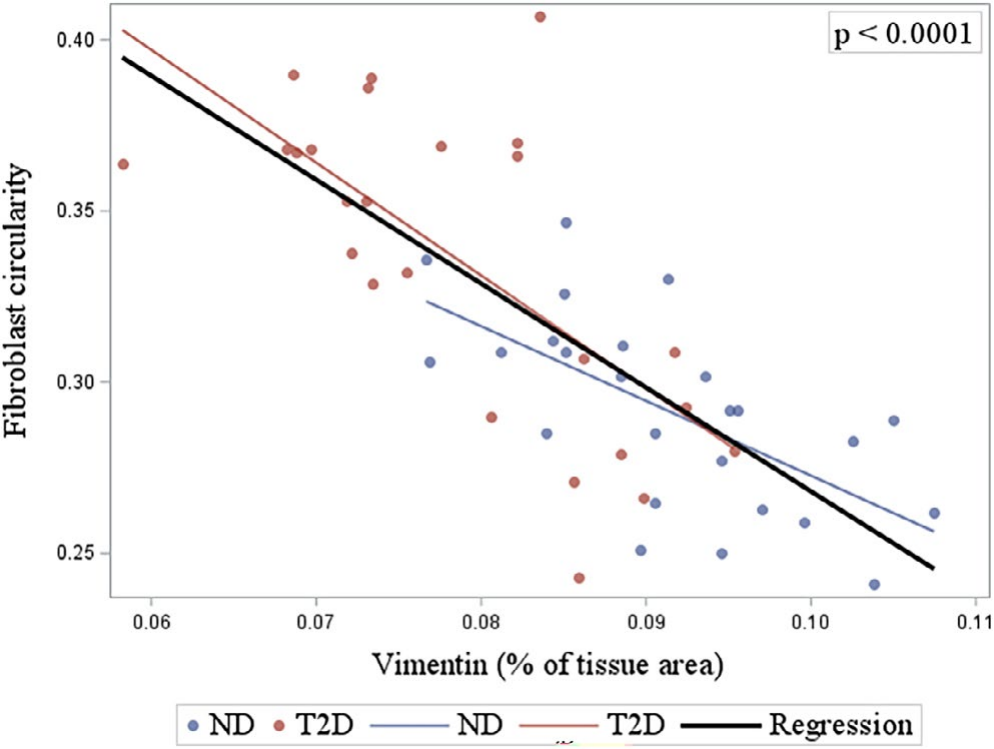
Relationship between fibroblast circularity and vimentin content of right atrial appendage (RAA) trabeculae. The relative abundance of vimentin in sections from RAA trabeculae was plotted against fibroblast circularity. Data points and regression lines for the ND group (blue) and the T2D group (red) are shown along with the combined regression line for both groups shown in black. The relationship between the two variables was examined using mixed model analysis. *p* ≤ 0.0001 indicates a statistically significant relationship between fibroblast circularity and vimentin label for the combined group data.

Fibroblasts in the T2D trabeculae showed no difference in tissue density (ND: 953 ± 180 per mm^2^; T2D: 780 ± 194 per mm^2^, *p* = 0.16, Figure 5c), cell size (ND: 60.7 ± 13.5 μm^2^; T2D: 49.0 ± 13.3 μm^2^, *p* = 0.21, Figure 5d), and minimum Feret's diameter (ND: 7.5 ± 0.8 μm; T2D: 6.6 ± 0.9 μm, *p* = 0.14, Figure 5e). However, a significant increase in fibroblast circularity was observed in T2D in comparison to ND (ND: 0.29 ± 0.02; T2D: 0.34 ± 0.03, *p* = 0.04, Figure 5f). Regression analysis carried out to examine the relationship between fibroblast and vimentin content in trabeculae showed that vimentin relative abundance was negatively associated with fibroblast circularity (*p* < 0.0001, Figure 6), but it was unrelated to the tissue density of fibroblasts (*p* = 0.66, Figure S2c). These findings suggest that the morphological changes decreased the area of vimentin labeling in fibroblasts, leading to a decrease in vimentin relative abundance in the T2D trabeculae.

### 3.3 Trabeculae contractile function

**FIGURE 7:**
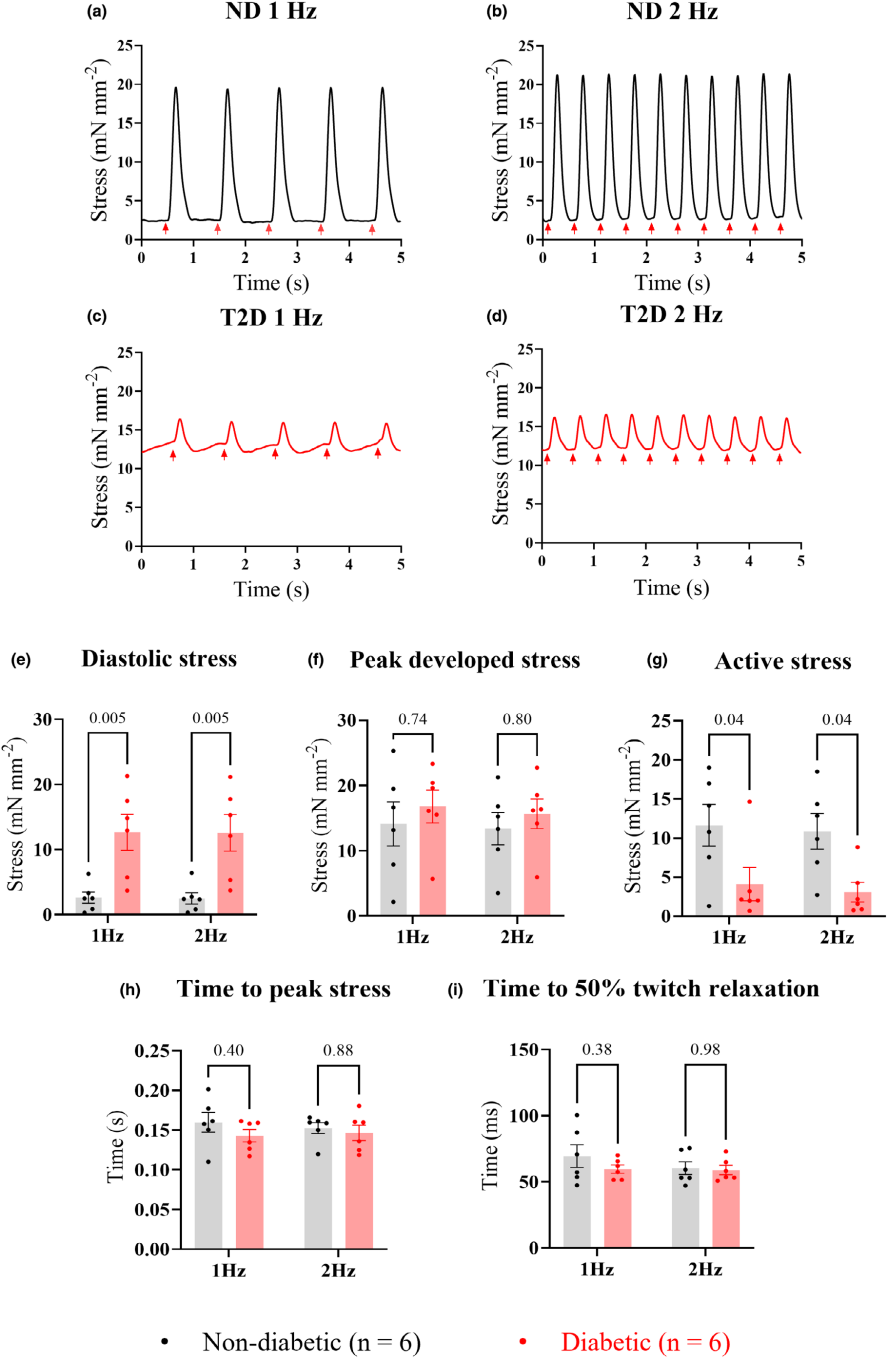
Contractile function of right atrial appendage (RAA) trabeculae at 1 Hz and 2 Hz stimulation. Panels (a)–(d) show examples of twitch stress from representative ND (a and b) and T2D (c and d) trabeculae at 1 Hz and 2 Hz stimulation frequencies. Red arrows indicate where each stimulus was applied. Trabeculae contractile parameters were averaged from consecutive steady‐state twitches from ND and T2D (*n* = 6 per group) for: diastolic stress (e); peak developed stress (f); active stress (g); time from stimulation to peak stress (h); and time from peak stress to 50% relaxation (i). Data are presented as mean ± SEM with *p* values shown above the bar graphs. Significance was determined by two‐way ANOVA for the group effect at each stimulation frequency, with *p* ≤ 0.05 indicating a difference between groups.

Figure 7 shows steady‐state functional data recorded from 6 ND and 6 T2D trabeculae at 37°C, paced at 1 and 2 Hz. T2D trabeculae had significantly higher diastolic stress (ND at 1 Hz: 2.5 ± 2.1 mN mm^−2^, and at 2 Hz: 2.5 ± 2.1 mN mm^−2^; T2D at 1 Hz: 12.7 ± 6.8 mN mm^−2^, and at 2 Hz: 12.6 ± 6.9 mN mm^−2^, *p* = 0.01, Figure 7e, Table 2), lower active stress (ND at 1 Hz: 11.9 ± 6.5 mN mm^−2^, and at 2 Hz: 10.9 ± 5.6 mN mm^−2^; T2D at 1 Hz: 4.1 ± 5.2 mN mm^−2^, and at 2 Hz: 3.1 ± 3.1 mN mm^−2^, *p* = 0.02, Figure 7g, Table 2), and no difference in peak developed stress (ND at 1 Hz: 14.4 ± 8.3 mN mm^−2^, and at 2 Hz: 13.4 ± 6.1 mN mm^−2^; T2D at 1 Hz: 16.8 ± 6.2 mN mm^−2^, and at 2 Hz: 15.7 ± 5.6 mN mm^−2^, *p* = 0.53, Figure 7f, Table 2). Time from stimulus to peak stress (ND: 1 Hz = 0.25 ± 0.19 s, 2 Hz = 0.15 ± 0.02 s; T2D: 1 Hz = 0.14 ± 0.02 s, 2 Hz = 0.15 ± 0.02 s, *p* = 0.16, Figure 7h, Table 2), and time from peak to 50% relaxation of stress (ND: 1 Hz = 69.6 ± 20.7 ms, 2 Hz = 60.4 ± 11.9 ms; T2D: 1 Hz = 59.7 ± 7.5 ms, 2 Hz = 59.0 ± 8.5 ms, *p* = 0.42, Figure 7i, Table 2) were also not different.

**TABLE 2:** Right atrial appendage (RAA) trabeculae contractile parameters at 1 Hz and 2 Hz stimulation frequencies.

Contractile parameters	ND group (*n* = 6)		T2D group (*n* = 6)		*p* Value
	1 Hz	2 Hz	1 Hz	2 Hz	
Diastolic stress (mN mm^−2^)	2.5 ± 2.1	2.5 ± 2.1	12.7 ± 6.8	12.6 ± 6.9	0.01
Peak developed stress (mN mm^−2^)	14.4 ± 8.3	13.4 ± 6.1	16.8 ± 6.2	15.7 ± 5.6	0.53
Active stress (mN mm^−2^)	11.9 ± 6.5	10.9 ± 5.6	4.1 ± 5.2	3.1 ± 3.1	0.02
Time from stimulus to peak stress (s)	0.25 ± 0.19	0.15 ± 0.02	0.14 ± 0.02	0.15 ± 0.02	0.16
Time from peak to 50% twitch relaxation (ms)	69.6 ± 20.7	60.4 ± 11.9	59.7 ± 7.5	59.0 ± 8.5	0.42

*Note*: Contractile parameters are presented as mean ± SD and averaged from consecutive steady‐state contractions at 1 Hz and 2 Hz stimulation. Statistical significances were determined by two‐way ANOVA to examine the group effect of stimulation frequency and diabetes; *p* ≤ 0.05 indicates a significant difference for the group effect.

**FIGURE 8:**
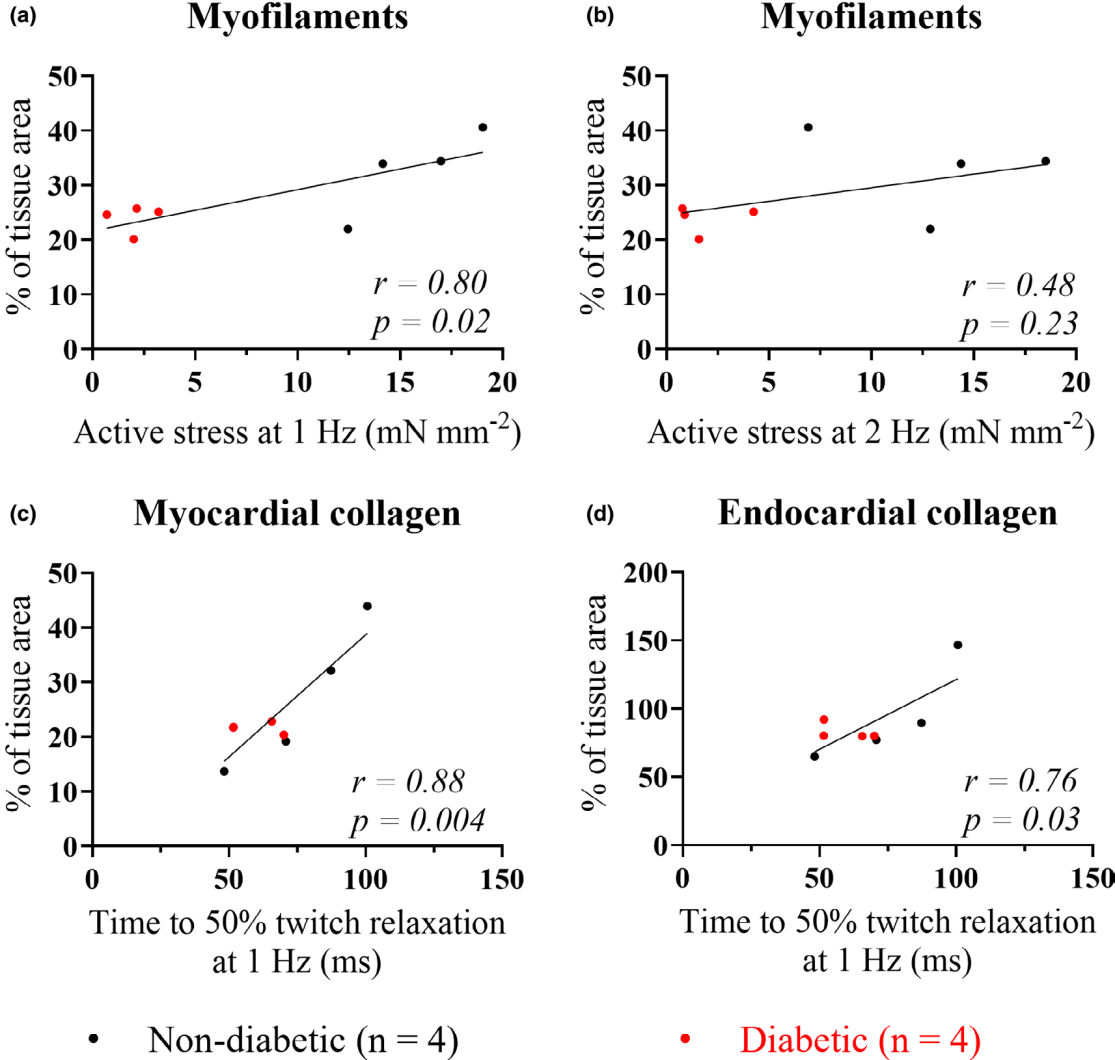
Correlation of trabeculae composition and contractile function. Panels (a) and (b) show mean data from fixed trabeculae transverse sections for myofilaments as % of tissue area plotted against active stress (mN mm^−2^) obtained from trabeculae taken from the same patient samples at 1 Hz (A) and 2 Hz (b) stimulation. Mean data are shown from trabeculae transverse sections for total collagen (I + III) as % of tissue area plotted against time from peak developed stress (mN mm^−2^) to 50% relaxation at 1 Hz for myocardial (c) and endocardial (d) tissue sections. Correlation between ND and T2D (*n* = 4 per group) was determined by two‐tailed tests; with *p* ≤ 0.05 indicating significance. Calculated *p* values and Pearson's correlation coefficients (*r*) are shown for each panel.

Correlation analyses were carried out to investigate the relationship between tissue composition and contractile function of trabeculae from the same patient samples. Trabeculae active stress produced and diastolic stress did not correlate with collagen content or the collagen I/III ratio (data not shown). However, trabeculae myofilament content positively correlated with active stress development (Figure 8a). A similar strong positive relationship was also found between trabeculae time to 50% relaxation of stress at 1 Hz and the relative abundance of collagen in the trabeculae myocardium (*r* = 0.88, *p* = 0.004, Figure 8c) and endocardium (*r* = 0.76, *p* = 0.03, Figure 8d) for both groups.

## 4 DISCUSSION

### 4.1 Patient clinical characteristics

The cohort of patients for this study were all scheduled to receive coronary artery bypass graft surgery at Auckland City Hospital. Patients with a HbA1c of less than 40 mmol mol^−1^ and/or no diagnostic history of diabetes mellitus (ND) were considered as “controls”. Thus, the investigation of atrial structure and function was carried out in the presence of coronary artery disease for both groups. ND and T2D groups were well‐matched in terms of age, clinical characteristics, and current medications. All patients had normal left ventricular function, apart from one T2D patient with left ventricular diastolic dysfunction and one ND patient with a mildly reduced LVEF. Previously, it has been reported that T2D patients have lower LVEF irrespective of the extent of coronary artery disease (Ehl et al., 2011). However, the LVEF was not significantly different for both ND and T2D groups in our study (Table 1). All patients were either overweight or obese based on their BMI, with most also diagnosed with hyperlipidemia (Table 1).

### 4.2 Composition of myocardium and endocardium

#### 4.2.1 Collagen

Histology of human left ventricular, right ventricular, and right atrial biopsies has consistently shown a greater extent of myocardial fibrosis in patients with T2D (Heerebeek et al., 2008; Lamberts et al., 2014; Regan et al., 1977; Rubler et al., 1972; Shimizu et al., 1993). However, RAA trabeculae collected from T2D patients in our study showed no major accumulation of collagen I or collagen III, irrespective of tissue orientation (Figure 2 & Figure 3), or when isolating myocardial or endocardial layers (Figure 2). Notably, T2D‐related myocardial fibrosis has been previously reported in patients with heart failure or detectable cardiomyopathy by echocardiography (Heerebeek et al., 2008; Regan et al., 1977; Rubler et al., 1972; Shimizu et al., 1993). However, the majority of patients in our study had normal left ventricular function prior to receiving heart surgery. Lamberts et al. (2014) quantified fibrous tissue in RAA biopsies from patients with coronary artery disease undergoing coronary artery bypass grafting surgery. They used picro‐sirius red staining, which labeled all collagen types, and showed evidence of myocardial fibrosis in individuals with T2D and increased BMI in the absence of any history of heart failure (Lamberts et al., 2014). Meanwhile, Campbell et al. (2011) similarly quantified type I and type III collagen in biopsies collected from the human left ventricle with coronary artery disease. The extent of myocardial fibrosis in T2D patients with normal left ventricular function was shown to be comparable to controls. In contrast, our study examined tissue sections acquired from both longitudinal and transverse sections of trabeculae dissected from the endocardial surface of RAA. Consistent with Campbell et al. (2011), we found no evidence of myocardial fibrosis at this stage of T2D. However, our findings do not rule out the possibility that diabetes‐driven myocardial fibrosis involves collagen types other than type I and type III.

Our study chose to quantify collagen types I and III as a measure of tissue fibrosis because they are the two most abundant extracellular proteins forming the structural scaffold for cardiomyocytes (Bashey et al., 1992). Previous investigations of hypertensive and ischemic myocardial fibrosis in animal models have reported distinct changes of collagen type I and III in terms of their relative abundance and alignment with cardiomyocytes (Pick et al., 1989; Weber et al., 1988). These changes progress toward unique patterns of myocardial fibrosis and alter myocardial contractile function differently depending on the etiology (Carroll et al., 1989; Jalil et al., 1989). However, we and others have shown that type I and III collagen are not causative factors for T2D‐related myocardial fibrosis in the early stages of diabetic heart disease in humans (Campbell et al., 2011). Our findings suggest that either myocardial fibrosis is not a feature of diabetic cardiomyopathy during the early phase or that other minor collagen types, for example, collagen type VI (Rasmussen et al., 2018; Spiro & Crowley, 1993; Steffensen & Rasmussen, 2018), also have a role.

Although the relative abundance of collagen type I and III remained unchanged in our study, a difference in the ratio of collagen I/III within the endocardium was observed between groups, with a decrease in the ratio observed for T2D (Figure 2). Pathological changes in the collagen I/III ratio have been previously reported in human post‐infarcted (Marijianowski et al., 1997) and dilated myocardium (Pauschinger et al., 1999), and were associated with fibrillation in the left atrial myocardium (Boldt et al., 2004). These differences were attributed to a disproportional increase in the relative abundance of type I collagen (Bishop et al., 1990). The mechanical role of type I collagen is reportedly to maintain myocardial rigidity, while type III collagen regulates myocardial elasticity (Borg et al., 1981). An observed reduction in the collagen I/III ratio might therefore indicate increased compliance of the T2D endocardium (Chung & Miller, 1974; McClain, 1974). However, it is unclear whether or not the difference in collagen I/III ratio between our patient cohorts represents an early phase in the development of dilated cardiomyopathy for T2D patients.

#### 4.2.2 Cardiomyocytes

Reduced cardiomyocyte abundance in T2D hearts has previously been associated with contractile deficit (Chowdhry et al., 2007; Frustaci et al., 2000; Munasinghe et al., 2016). Our results showed that transversely sectioned trabeculae from T2D patients had a reduced area occupied by myofilaments (Figure 3), as observed previously (Jones et al., 2023). We therefore determined whether the apparent loss of myofilaments in trabeculae cross‐sections resulted from a reduction in the number of cardiomyocytes per trabecula, a decrease in myofilament content per cardiomyocyte, or both. Investigation of cardiomyocytes from both groups showed no change in myofilament density or morphology in T2D, and that myofilament content of cardiomyocytes (% of cross‐sectional area occupied by myofilaments) did not differ between groups (Figure 4).

A plausible explanation for the reduced relative abundance of myofilaments in T2D trabeculae is therefore an increase in the ECM volume (Figure 3a,b). Since the collagen content (type I and type III) was not significantly different between groups (Figures 2 and 3), it is probable that other non‐collagen components, for instance, lipid droplets, contributed to the increased ECM in the T2D myocardium. Given that all participating patients were overweight or obese, and 11 out of 14 patients had hyperlipidemia (Table 1), myocardial lipid infiltration (myocardial steatosis) is likely to be prevalent, although not exclusive to samples from the T2D group. However, a feature of T2D is the impaired ability to transport glucose from the blood into the cells (glucose intolerance). This shifts cardiomyocyte substrate utilization from primarily glucose to lipids for energy production (Herrero et al., 2006), favoring lipid storage. Potentially, T2D myocardium might therefore have exacerbated myocardial steatosis in the presence of hyperlipidemia (McGavock et al., 2007).

#### 4.2.3 Fibroblasts

Fibroblasts are considered to be the primary cellular effectors of fibrotic remodeling in the myocardium, with evidence from animal models of T2D showing enhanced fibroblast proliferation and collagen secretion (Cohen et al., 2021; Fowlkes et al., 2013; Hutchinson et al., 2013). Our study found that fibroblasts constituted a similar percentage of cells in the ND and T2D myocardium (Figure S2b), with no difference in the density of fibroblasts between groups (Figure 5). Our findings are consistent with Sedgwick et al. (2014), who showed that the proliferation rate of human RAA fibroblasts in vitro was not affected by the diabetic status of patients. In contrast, fibroblasts from animal models of T2D typically had a more fibrotic phenotype, featured by increased proliferation and collagen secretion (Cohen et al., 2021; Fowlkes et al., 2013; Hutchinson et al., 2013). The difference in fibroblast adaptation in T2D might be due to differences in the progression of diabetic heart disease between animal models and humans.

Vimentin is an intracellular intermediate filament protein commonly used as a biomarker of fibroblasts (Dulbecco et al., 1981). It labels fibroblasts of various phenotypes, but it also labels cells other than fibroblasts within the mesenchymal lineage. Therefore, vimentin was co‐labeled with type I collagen to improve the specificity of localizing fibroblasts in our trabeculae (Moore‐Morris et al., 2014). Our results showed that T2D trabeculae contained less vimentin content compared to ND (Figure 3), with morphological differences in vimentin‐labeled fibroblasts (Figure 6 and Figure S2c). Observations from Dulbecco et al. (1981) who were the first to label vimentin in fibroblasts, suggest that the rearrangement of vimentin in fibroblasts by elongation indicates cell movement, and such rearrangement exposes more epitopes for recognition by the antibody (Dulbecco et al., 1983). Thus, the reduced area of vimentin labeling (Figure 3) and increased circularity of fibroblasts (i.e., less elongated fibroblasts) (Figure 5) in our study may indicate that fibroblasts in T2D trabeculae were more static. Restricted cell mobility could imply that ECM rigidity was increased, consistent with reports that T2D increases the cross‐linking of extracellular proteins (advanced glycation end‐products) (Monnier et al., 1984). T2D trabeculae had an increase in diastolic stress (Figure 7e), which might also result from increased rigidity of the ECM. Additionally, the changes in fibroblast morphology we observed could provide evidence of fibrotic transformation in response to T2D (Alex et al., 2023; Hillsley et al., 2022), contributing to the observed shift in the collagen I/III ratio (Figure 2). Therefore, the early morphological changes in fibroblast circularity may be a predictive factor of future fibrotic remodeling of the T2D heart.

Alternatively, the changes in fibroblast morphology might simply result from the decreased density of cardiomyocytes (indexed as phalloidin labelling of myofilaments) in diabetic myocardium. Fibroblasts in healthy myocardium are typically located in the interstitium with an elongated shape, attributed to the densely packed cardiomyocytes (Goldsmith et al., 2004). Fu et al. (2018) previously showed that fibroblasts in the mouse post‐infarcted myocardium underwent progressive changes in morphology as cardiomyocytes were replaced with scar tissue. Notably, fibroblasts further elongated and proliferated post‐infarction, indicating their fibrotic activation (Fu et al., 2018). This was, however, not observed in our diabetic tissue, suggesting a differential response of fibroblasts toward diabetes and ischemic insults.

### 4.3 Trabeculae contractile function

RAA trabeculae are suitable for measurements of contractile stress since the cardiomyocytes are aligned in parallel with the longitudinal axis of the muscles. Stress measurements in response to electrical stimulation from our RAA trabeculae were comparable to those previously reported from human left ventricular trabeculae (Munro et al., 2018; Vahl et al., 1997), suggesting that RAA trabeculae are suitable for assessing myocardial function.

Relative to ND, T2D trabeculae had increased diastolic stress irrespective of stimulation frequency (Figure 7e). However, diastolic stress was unrelated to the collagen content or collagen I/III ratio in trabeculae, meaning the increased diastolic stress was likely caused by factors other than fibrotic remodeling of the T2D myocardium. Previously, disrupted Ca^2+^ cycling (Jones et al., 2023) and altered contractile protein interactions (Musgrave et al., 2025) have been suggested to increase cardiomyocyte passive stiffness, thereby increasing the diastolic stress in the diabetic heart, independent of myocardial fibrosis (Campbell et al., 2003; King et al., 2010; Sequeira et al., 2015). In addition, titin and the microtubule network in cardiomyocytes are also key regulators of myocardial stiffness in parallel to collagen (Hamdani & Paulus, 2013). T2D has been shown to induce phosphorylation of titin in humans, which substantially increases the stiffness of cardiomyocytes (Hopf et al., 2018). In comparison, a dense and reinforced microtubule network has also been shown to impede the diastolic function of human failing hearts (Caporizzo et al., 2020). However, the relationship between T2D, microtubules, and cardiomyocyte passive stiffness in trabeculae in our study remains unclear.

T2D trabeculae showed a significant reduction in active stress produced when compared to ND (Figure 7g). This may result from the observed reduction in myofilament content in T2D trabeculae (Figure 8a,b) but does not rule out disrupted Ca^2+^ cycling. Both observations were previously reported in trabeculae from a similar cohort of T2D patients (Jones et al., 2023).

The time from stimulation to peak stress, and the time from peak stress to 50% relaxation, were comparable between the groups (Figure 7h,i), indicating a similar time course for contraction and relaxation of trabeculae from the two groups. We additionally showed a strong positive correlation between the time to trabeculae relaxation and the collagen content (Figure 8c,d), although this correlation was diminished at 2 Hz stimulation frequency (Figure S2d).

## 5 CONCLUSION

This study compared the contractile response of human RAA trabeculae and key ECM components in tissue derived from the same ND and T2D patient samples. All patients had underlying coronary artery disease, but trabeculae from T2D patients had increased diastolic stress, which was unrelated to the ECM composition. We also observed morphological differences in fibroblasts from T2D trabeculae and a difference in the collagen type I/III ratio, which could be early predictors of structural remodeling in this cohort of T2D patients. T2D trabeculae had reduced myofilament content in comparison to ND, contributing to the lower active stress development. However, the loss of myofilaments in T2D was not explained by a reduction in the relative number of cardiomyocytes nor by evidence of morphological changes in cardiomyocytes. Therefore, it is possible that differences in the relative area of myofilaments per T2D trabeculae transverse section result from increases in myocardial ECM components. Importantly, the observed increase in diastolic stress and decreased active stress production implies that this cohort of T2D patients is progressing toward the development of systolic and diastolic heart failure.

## AUTHOR CONTRIBUTIONS

M‐LW and ASP conceived and co‐supervised the project. NK provided clinical insight and assisted in tissue acquisition. LTZ carried out immunohistochemistry and confocal imaging. ASP conducted experiments to obtain contractile data. LTZ analyzed the data. All authors contributed to the preparation of the final manuscript.

## FUNDING INFORMATION

This work was supported by the Auckland Medical Research Foundation (1124006 to M‐LW & ASP), Maurice and Phyllis Paykel Trust (231109 to M‐LW), and the Health Research Council of New Zealand (24‐747 Emerging Researcher First Grant to ASP).

## CONFLICT OF INTEREST STATEMENT

The authors declare no conflicts of interest.

## ETHICS STATEMENT

The collection and use of human tissue in this study was approved by the Auckland District Health Board (ADHB) Research Committee (reference: A+7593) and the Southern Health and Disability Ethics Committee (reference: PR/6432). Informed consent was obtained in writing from patients prior to routine coronary artery bypass grafting surgery for a small sample of right atrial appendage tissue to be obtained for research into diabetic heart disease at the University of Auckland. The patient consenting process complied with the principles outlined in the Declaration of Helsinki.

## Supporting information


Appendix S1.

Figure S2.

## Data Availability

Data will be made available on request.

## References

[phy270509-bib-0001] Alex, L. , Tuleta, I. , Hanna, A. , & Frangogiannis, N. G. (2023). Diabetes induces cardiac fibroblast activation, promoting a matrix‐preserving nonmyofibroblast phenotype, without stimulating pericyte to fibroblast conversion. Journal of the American Heart Association, 12(6), e027463. 10.1161/JAHA.122.027463 36892073 PMC10111546

[phy270509-bib-0002] Bashey, R. I. , Martinez‐Hernandez, A. , & Jimenez, S. A. (1992). Isolation, characterization, and localization of cardiac collagen type VI. Associations with other extracellular matrix components. Circulation Research, 70(5), 1006–1017. 10.1161/01.RES.70.5.1006 1568294

[phy270509-bib-0003] Bishop, J. E. , Greenbaum, R. , Gibson, D. G. , Yacoub, M. , & Laurent, G. J. (1990). Enhanced deposition of predominantly type I collagen in myocardial disease. Journal of Molecular and Cellular Cardiology, 22(10), 1157–1165. 10.1016/0022-2828(90)90079-H 2095438

[phy270509-bib-0004] Boldt, A. , Wetzel, U. , Lauschke, J. , Weigl, J. , Gummert, J. , Hindricks, G. , Kottkamp, H. , & Dhein, S. (2004). Fibrosis in left atrial tissue of patients with atrial fibrillation with and without underlying mitral valve disease. Heart, 90(4), 400–405. 10.1136/hrt.2003.015347 15020515 PMC1768173

[phy270509-bib-0005] Borg, T. K. , Ranson, W. F. , Moslehy, F. A. , & Caulfield, J. B. (1981). Structural basis of ventricular stiffness. Laboratory Investigation, 44(1), 49–54. 7453130

[phy270509-bib-0006] Campbell, D. J. , Somaratne, J. B. , Jenkins, A. J. , Prior, D. L. , Yii, M. , Kenny, J. F. , Newcomb, A. E. , Schalkwijk, C. G. , Black, M. J. , & Kelly, D. J. (2011). Impact of type 2 diabetes and the metabolic syndrome on myocardial structure and microvasculature of men with coronary artery disease. Cardiovascular Diabetology, 10, 80. 10.1186/1475-2840-10-80 21929744 PMC3182888

[phy270509-bib-0007] Campbell, K. S. , Patel, J. R. , & Moss, R. L. (2003). Cycling cross‐bridges increase myocardial stiffness at submaximal levels of Ca2+ activation. Biophysical Journal, 84(6), 3807–3815. 10.1016/S0006-3495(03)75108-X 12770886 PMC1302962

[phy270509-bib-0008] Caporizzo, M. A. , Chen, C. Y. , Bedi, K. , Margulies, K. B. , & Prosser, B. L. (2020). Microtubules increase diastolic stiffness in failing human cardiomyocytes and myocardium. Circulation, 141(11), 902–915. 10.1161/circulationaha.119.043930 31941365 PMC7078018

[phy270509-bib-0009] Carroll, E. P. , Janicki, J. S. , Pick, R. , & Weber, K. T. (1989). Myocardial stiffness and reparative fibrosis following coronary embolisation in the rat. Cardiovascular Research, 23(8), 655–661. 10.1093/cvr/23.8.655 2598220

[phy270509-bib-0010] Celentano, A. , Vaccaro, O. , Tammaro, P. , Galderisi, M. , Crivaro, M. , Oliviero, M. , Imperatore, G. , Palmieri, V. , Iovino, V. , Riccardi, G. , & Divitiis, O. d. (1995). Early abnormalities of cardiac function in non‐insulin‐dependent diabetes mellitus and impaired glucose tolerance. The American Journal of Cardiology, 76(16), 1173–1176. 10.1016/S0002-9149(99)80330-0 7484905

[phy270509-bib-0011] Chowdhry, M. F. , Vohra, H. A. , & Galiñanes, M. (2007). Diabetes increases apoptosis and necrosis in both ischemic and nonischemic human myocardium: Role of caspases and poly‐adenosine diphosphate‐ribose polymerase. The Journal of Thoracic and Cardiovascular Surgery, 134(1), 124–131. 10.1016/j.jtcvs.2006.12.059 17599497

[phy270509-bib-0012] Chung, E. , & Miller, E. J. (1974). Collagen polymorphism: Characterization of molecules with the chain composition [α1 (III)] 3 in human tissues. Science, 183(4130), 1200–1201. 10.1126/science.183.4130.1200 4812351

[phy270509-bib-0013] Cohen, C. D. , De Blasio, M. J. , Lee, M. K. S. , Farrugia, G. E. , Prakoso, D. , Krstevski, C. , Deo, M. , Donner, D. G. , Kiriazis, H. , Flynn, M. C. , Gaynor, T. L. , Murphy, A. J. , Drummond, G. R. , Pinto, A. R. , & Ritchie, R. H. (2021). Diastolic dysfunction in a pre‐clinical model of diabetes is associated with changes in the cardiac non‐myocyte cellular composition. Cardiovascular Diabetology, 20(1), 116. 10.1186/s12933-021-01303-9 34074290 PMC8170962

[phy270509-bib-0014] Dulbecco, R. , Allen, R. , Okada, S. , & Bowman, M. (1983). Functional changes of intermediate filaments in fibroblastic cells revealed by a monoclonal antibody. Proceedings of the National Academy of Sciences of The United States of America, 80(7), 1915–1918. 10.1073/pnas.80.7.1915 6132382 PMC393721

[phy270509-bib-0015] Dulbecco, R. , Unger, M. , Bologna, M. , Battifora, H. , Syka, P. , & Okada, S. (1981). Cross‐reactivity between Thy‐1 and a component of intermediate filaments demonstrated using a monoclonal antibody. Nature, 292(5825), 772–774. 10.1038/292772a0 6115318

[phy270509-bib-0016] Ehl, N. F. , Kühne, M. , Brinkert, M. , Müller‐Brand, J. , & Zellweger, M. J. (2011). Diabetes reduces left ventricular ejection fraction‐‐irrespective of presence and extent of coronary artery disease. European Journal of Endocrinology, 165(6), 945–951. 10.1530/eje-11-0687 21903896

[phy270509-bib-0017] Fowlkes, V. , Clark, J. , Fix, C. , Law, B. A. , Morales, M. O. , Qiao, X. , Ako‐Asare, K. , Goldsmith, J. G. , Carver, W. , Murray, D. B. , & Goldsmith, E. C. (2013). Type II diabetes promotes a myofibroblast phenotype in cardiac fibroblasts. Life Sciences, 92(11), 669–676. 10.1016/j.lfs.2013.01.003 23333820 PMC3810176

[phy270509-bib-0018] Frustaci, A. , Kajstura, J. , Chimenti, C. , Jakoniuk, I. , Leri, A. , Maseri, A. , Nadal‐Ginard, B. , & Anversa, P. (2000). Myocardial cell death in human diabetes. Circulation Research, 87(12), 1123–1132. 10.1161/01.RES.87.12.1123 11110769

[phy270509-bib-0019] Fu, X. , Khalil, H. , Kanisicak, O. , Boyer, J. G. , Vagnozzi, R. J. , Maliken, B. D. , Sargent, M. A. , Prasad, V. , Valiente‐Alandi, I. , Blaxall, B. C. , & Molkentin, J. D. (2018). Specialized fibroblast differentiated states underlie scar formation in the infarcted mouse heart. The Journal of Clinical Investigation, 128(5), 2127–2143. 10.1172/jci98215 29664017 PMC5957472

[phy270509-bib-0020] Goldsmith, E. C. , Hoffman, A. , Morales, M. O. , Potts, J. D. , Price, R. L. , McFadden, A. , Rice, M. , & Borg, T. K. (2004). Organization of fibroblasts in the heart. Developmental Dynamics, 230(4), 787–794. 10.1002/dvdy.20095 15254913

[phy270509-bib-0021] Hamdani, N. , & Paulus, W. J. (2013). Myocardial titin and collagen in cardiac diastolic dysfunction. Circulation, 128(1), 5–8. 10.1161/CIRCULATIONAHA.113.003437 23709670

[phy270509-bib-0022] Heerebeek, L. v. , Hamdani, N. , Handoko, M. L. , Falcao‐Pires, I. , Musters, R. J. , Kupreishvili, K. , Ijsselmuiden, A. J. J. , Schalkwijk, C. G. , Bronzwaer, J. G. F. , Diamant, M. , Borbély, A. , Velden, J. v. d. , Stienen, G. J. M. , Laarman, G. J. , Niessen, H. W. M. , & Paulus, W. J. (2008). Diastolic stiffness of the failing diabetic heart. Circulation, 117(1), 43–51. 10.1161/CIRCULATIONAHA.107.728550 18071071

[phy270509-bib-0023] Herrero, P. , Peterson, L. R. , McGill, J. B. , Matthew, S. , Lesniak, D. , Dence, C. , & Gropler, R. J. (2006). Increased myocardial fatty acid metabolism in patients with type 1 diabetes mellitus. Journal of the American College of Cardiology, 47(3), 598–604. 10.1016/j.jacc.2005.09.030 16458143

[phy270509-bib-0024] Hillsley, A. , Santoso, M. S. , Engels, S. M. , Halwachs, K. N. , Contreras, L. M. , & Rosales, A. M. (2022). A strategy to quantify myofibroblast activation on a continuous spectrum. Scientific Reports, 12(1), 12239. 10.1038/s41598-022-16158-7 35851602 PMC9293987

[phy270509-bib-0025] Hopf, A. E. , Andresen, C. , Kötter, S. , Isić, M. , Ulrich, K. , Sahin, S. , Bongardt, S. , Röll, W. , Drove, F. , Scheerer, N. , Vandekerckhove, L. , De Keulenaer, G. W. , Hamdani, N. , Linke, W. A. , & Krüger, M. (2018). Diabetes‐induced cardiomyocyte passive stiffening is caused by impaired insulin‐dependent titin modification and can Be modulated by neuregulin‐1. Circulation Research, 123(3), 342–355. 10.1161/circresaha.117.312166 29760016

[phy270509-bib-0026] Hutchinson, K. R. , Lord, C. K. , West, T. A. , & Stewart, J. A., Jr. (2013). Cardiac fibroblast‐dependent extracellular matrix accumulation is associated with diastolic stiffness in type 2 diabetes. PLoS One, 8(8), e72080. 10.1371/journal.pone.0072080 23991045 PMC3749105

[phy270509-bib-0027] Jalil, J. E. , Doering, C. W. , Janicki, J. S. , Pick, R. , Shroff, S. G. , & Weber, K. T. (1989). Fibrillar collagen and myocardial stiffness in the intact hypertrophied rat left ventricle. Circulation Research, 64(6), 1041–1050. 10.1161/01.res.64.6.1041 2524288

[phy270509-bib-0028] Jones, T. L. M. , Kaur, S. , Kang, N. , Ruygrok, P. N. , & Ward, M.‐L. (2023). Impaired calcium handling mechanisms in atrial trabeculae of diabetic patients. Physiological Reports, 11(3), e15599. 10.14814/phy2.15599 36750180 PMC9904963

[phy270509-bib-0029] King, N. M. P. , Methawasin, M. , Nedrud, J. , Harrell, N. , Chung, C. S. , Helmes, M. , & Granzier, H. (2010). Mouse intact cardiac myocyte mechanics: Cross‐bridge and titin‐based stress in unactivated cells. Journal of General Physiology, 137(1), 81–91. 10.1085/jgp.201010499 PMC3010058 21187335

[phy270509-bib-0030] Lamberts, R. R. , Lingam, S. J. , Wang, H. Y. , Bollen, I. A. , Hughes, G. , Galvin, I. F. , Bunton, R. W. , Bahn, A. , Katare, R. , Baldi, J. C. , Williams, M. J. , Saxena, P. , Coffey, S. , & Jones, P. P. (2014). Impaired relaxation despite upregulated calcium‐handling protein atrial myocardium from type 2 diabetic patients with preserved ejection fraction. Cardiovascular Diabetology, 13, 72. 10.1186/1475-2840-13-72 24708792 PMC3997226

[phy270509-bib-0031] Leyden, E. v. (1881). Asthma and diabetes mellitus. Zeutschr Klin Med, 3, 358–364.

[phy270509-bib-0032] Marijianowski, M. M. H. , Teeling, P. , & Becker, A. E. (1997). Remodeling after myocardial infarction in humans is not associated with interstitial fibrosis of noninfarcted myocardium. Journal of the American College of Cardiology, 30(1), 76–82. 10.1016/S0735-1097(97)00100-9 9207624

[phy270509-bib-0033] McClain, P. E. (1974). Characterization of cardiac muscle collagen: Molecular heterogeneity. Journal of Biological Chemistry, 249(7), 2303–2311. 10.1016/S0021-9258(19)42832-9 4362072

[phy270509-bib-0034] McGavock, J. M. , Lingvay, I. , Zib, I. , Tillery, T. , Salas, N. , Unger, R. , Levine, B. D. , Raskin, P. , Victor, R. G. , & Szczepaniak, L. S. (2007). Cardiac steatosis in diabetes mellitus: A 1H‐magnetic resonance spectroscopy study. Circulation, 116(10), 1170–1175. 10.1161/circulationaha.106.645614 17698735

[phy270509-bib-0035] Mizushige, K. , Yao, L. , Noma, T. , Kiyomoto, H. , Yu, Y. , Hosomi, N. , Ohmori, K. , & Matsuo, H. (2000). Alteration in left ventricular diastolic filling and accumulation of myocardial collagen at insulin‐resistant prediabetic stage of a type II diabetic rat model. Circulation, 101(8), 899–907. 10.1161/01.cir.101.8.899 10694530

[phy270509-bib-0036] Monnier, V. M. , Kohn, R. R. , & Cerami, A. (1984). Accelerated age‐related browning of human collagen in diabetes mellitus. Proceedings of the National Academy of Sciences of The United States of America, 81(2), 583–587. 10.1073/pnas.81.2.583 6582514 PMC344723

[phy270509-bib-0037] Montaigne, D. , Marechal, X. , Coisne, A. , Debry, N. , Modine, T. , Fayad, G. , Potelle, C. , El Arid, J. M. , Mouton, S. , Sebti, Y. , Duez, H. , Preau, S. , Remy‐Jouet, I. , Zerimech, F. , Koussa, M. , Richard, V. , Neviere, R. , Edme, J. L. , Lefebvre, P. , & Staels, B. (2014). Myocardial contractile dysfunction is associated with impaired mitochondrial function and dynamics in type 2 diabetic but not in obese patients. Circulation, 130(7), 554–564. 10.1161/circulationaha.113.008476 24928681

[phy270509-bib-0038] Moore‐Morris, T. , Guimarães‐Camboa, N. , Banerjee, I. , Zambon, A. C. , Kisseleva, T. , Velayoudon, A. , Stallcup, W. B. , Gu, Y. , Dalton, N. D. , Cedenilla, M. , Gomez‐Amaro, R. , Zhou, B. , Brenner, D. A. , Peterson, K. L. , Chen, J. , & Evans, S. M. (2014). Resident fibroblast lineages mediate pressure overload‐induced cardiac fibrosis. The Journal of Clinical Investigation, 124(7), 2921–2934. 10.1172/jci74783 24937432 PMC4071409

[phy270509-bib-0039] Munasinghe, P. E. , Riu, F. , Dixit, P. , Edamatsu, M. , Saxena, P. , Hamer, N. S. J. , Galvin, I. F. , Bunton, R. W. , Lequeux, S. , Jones, G. , Lamberts, R. R. , Emanueli, C. , Madeddu, P. , & Katare, R. (2016). Type‐2 diabetes increases autophagy in the human heart through promotion of Beclin‐1 mediated pathway. International Journal of Cardiology, 202, 13–20. 10.1016/j.ijcard.2015.08.111 26386349

[phy270509-bib-0040] Munro, M. L. , Shen, X. , Ward, M. , Ruygrok, P. N. , Crossman, D. J. , & Soeller, C. (2018). Highly variable contractile performance correlates with myocyte content in trabeculae from failing human hearts. Scientific Reports, 8(1), 2957. 10.1038/s41598-018-21199-y 29440728 PMC5811450

[phy270509-bib-0041] Musgrave, J. H. , Han, J. C. , Ward, M. L. , Kang, N. , Taberner, A. J. , & Tran, K. (2025). Human cardiac tissues produce lower contractile stress and exhibit slower cross‐bridge cycling in type 2 diabetes. Cardiovascular Diabetology, 24(1), 266. 10.1186/s12933-025-02820-7 40611280 PMC12224372

[phy270509-bib-0042] Ng, A. C. , Auger, D. , Delgado, V. , van Elderen, S. G. , Bertini, M. , Siebelink, H. M. , van der Geest, R. J. , Bonetti, C. , van der Velde, E. T. , de Roos, A. , Smit, J. W. , Leung, D. Y. , Bax, J. J. , & Lamb, H. J. (2012). Association between diffuse myocardial fibrosis by cardiac magnetic resonance contrast‐enhanced T_1_ mapping and subclinical myocardial dysfunction in diabetic patients: A pilot study. Circulation. Cardiovascular Imaging, 5(1), 51–59. 10.1161/circimaging.111.965608 22135399

[phy270509-bib-0043] Pauschinger, M. , Knopf, D. , Petschauer, S. , Doerner, A. , Poller, W. , Schwimmbeck, P. L. , Kühl, U. , & Schultheiss, H.‐P. (1999). Dilated cardiomyopathy is associated with significant changes in collagen type I/III ratio. Circulation, 99(21), 2750–2756. 10.1161/01.CIR.99.21.2750 10351968

[phy270509-bib-0044] Pick, R. , Janicki, J. S. , & Weber, K. T. (1989). Myocardial fibrosis in nonhuman primate with pressure overload hypertrophy. The American Journal of Pathology, 135(5), 771–781. 2530905 PMC1880105

[phy270509-bib-0045] Rasmussen, D. G. K. , Hansen, T. W. , von Scholten, B. J. , Nielsen, S. H. , Reinhard, H. , Parving, H. H. , Tepel, M. , Karsdal, M. A. , Jacobsen, P. K. , Genovese, F. , & Rossing, P. (2018). Higher collagen VI formation is associated with all‐cause mortality in patients with type 2 diabetes and microalbuminuria. Diabetes Care, 41(7), 1493–1500. 10.2337/dc17-2392 29643059

[phy270509-bib-0046] Regan, T. J. , Lyons, M. M. , Ahmed, S. S. , Levinson, G. E. , Oldewurtel, H. A. , Ahmad, M. R. , & Haider, B. (1977). Evidence for cardiomyopathy in familial diabetes mellitus. The Journal of Clinical Investigation, 60(4), 884–899. 10.1172/jci108843 893679 PMC372437

[phy270509-bib-0047] Rubler, S. , Dlugash, J. , Yuceoglu, Y. Z. , Kumral, T. , Branwood, A. W. , & Grishman, A. (1972). New type of cardiomyopathy associated with diabetic glomerulosclerosis. The American Journal of Cardiology, 30(6), 595–602. 10.1016/0002-9149(72)90595-4 4263660

[phy270509-bib-0048] Sakakibara, M. , Hirashiki, A. , Cheng, X. W. , Bando, Y. , Ohshima, K. , Okumura, T. , Funahashi, H. , Ohshima, S. , & Murohara, T. (2011). Association of diabetes mellitus with myocardial collagen accumulation and relaxation impairment in patients with dilated cardiomyopathy. Diabetes Research and Clinical Practice, 92(3), 348–355. 10.1016/j.diabres.2011.02.023 21414680

[phy270509-bib-0049] Schindelin, J. , Arganda‐Carreras, I. , Frise, E. , Kaynig, V. , Longair, M. , Pietzsch, T. , Preibisch, S. , Rueden, C. , Saalfeld, S. , Schmid, B. , Tinevez, J.‐Y. , White, D. J. , Hartenstein, V. , Eliceiri, K. , Tomancak, P. , & Cardona, A. (2012). Fiji: An open‐source platform for biological‐image analysis. Nature Methods, 9(7), 676–682. 10.1038/nmeth.2019 22743772 PMC3855844

[phy270509-bib-0050] Sedgwick, B. , Riches, K. , Bageghni, S. A. , O'Regan, D. J. , Porter, K. E. , & Turner, N. A. (2014). Investigating inherent functional differences between human cardiac fibroblasts cultured from nondiabetic and type 2 diabetic donors. Cardiovascular Pathology, 23(4), 204–210. 10.1016/j.carpath.2014.03.004 24746387

[phy270509-bib-0051] Sequeira, V. , Najafi, A. , McConnell, M. , Fowler, E. D. , Bollen, I. A. E. , Wüst, R. C. I. , dos Remedios, C. , Helmes, M. , White, E. , Stienen, G. J. M. , Tardiff, J. , Kuster, D. W. D. , & van der Velden, J. (2015). Synergistic role of ADP and Ca2+ in diastolic myocardial stiffness. The Journal of Physiology, 593(17), 3899–3916. 10.1113/JP270354 26096258 PMC4575576

[phy270509-bib-0052] Shah, A. D. , Langenberg, C. , Rapsomaniki, E. , Denaxas, S. , Pujades‐Rodriguez, M. , Gale, C. P. , Deanfield, J. , Smeeth, L. , Timmis, A. , & Hemingway, H. (2015). Type 2 diabetes and incidence of cardiovascular diseases: A cohort study in 1·9 million people. The Lancet Diabetes and Endocrinology, 3(2), 105–113. 10.1016/s2213-8587(14)70219-0 25466521 PMC4303913

[phy270509-bib-0053] Shimizu, M. , Umeda, K. , Sugihara, N. , Yoshio, H. , Ino, H. , Takeda, R. , Okada, Y. , & Nakanishi, I. (1993). Collagen remodelling in myocardia of patients with diabetes. Journal of Clinical Pathology, 46(1), 32–36. 10.1136/jcp.46.1.32 7679418 PMC501107

[phy270509-bib-0054] Spiro, M. J. , & Crowley, T. J. (1993). Increased rat myocardial type VI collagen in diabetes mellitus and hypertension. Diabetologia, 36(2), 93–98. 10.1007/bf00400687 8458534

[phy270509-bib-0055] Steffensen, L. B. , & Rasmussen, L. M. (2018). A role for collagen type IV in cardiovascular disease? American Journal of Physiology. Heart and Circulatory Physiology, 315(3), H610–h625. 10.1152/ajpheart.00070.2018 29677463

[phy270509-bib-0056] Vahl, C. F. , Timek, T. , Bonz, A. , Kochsiek, N. , Fuchs, H. , Schäffer, L. , Rosenberg, M. , Dillmann, R. , & Hagl, S. (1997). Myocardial length‐force relationship in end stage dilated cardiomyopathy and normal human myocardium: Analysis of intact and skinned left ventricular trabeculae obtained during 11 heart transplantations. Basic Research in Cardiology, 92(4), 261–270. 10.1007/BF00788521 9342433

[phy270509-bib-0057] Weber, K. T. , Janicki, J. S. , Shroff, S. G. , Pick, R. , Chen, R. M. , & Bashey, R. I. (1988). Collagen remodeling of the pressure‐overloaded, hypertrophied nonhuman primate myocardium. Circulation Research, 62(4), 757–765. 10.1161/01.res.62.4.757 2964945

[phy270509-bib-0058] Zhang, L. , Cannell, M. B. , Phillips, A. R. , Cooper, G. J. , & Ward, M. L. (2008). Altered calcium homeostasis does not explain the contractile deficit of diabetic cardiomyopathy. Diabetes, 57(8), 2158–2166. 10.2337/db08-0140 18492789 PMC2494698

[phy270509-bib-0059] Zhou, B. , Rayner, A. W. , Gregg, E. W. , Sheffer, K. E. , Carrillo‐Larco, R. M. , Bennett, J. E. , Shaw, J. E. , Paciorek, C. J. , Singleton, R. K. , Barradas Pires, A. , Stevens, G. A. , Danaei, G. , Lhoste, V. P. F. , Phelps, N. H. , Heap, R. A. , Jain, L. , D'Ailhaud De Brisis, Y. , Galeazzi, A. , Kengne, A. , … Adams, R. J. (2024). Worldwide trends in diabetes prevalence and treatment from 1990 to 2022: A pooled analysis of 1108 population‐representative studies with 141 million participants. The Lancet, 404(10467), 2077–2093. 10.1016/S0140-6736(24)02317-1 PMC7616842 39549716

