# Supplementary material for: Myocardial composition and contractile function of right atrial trabeculae from type 2 diabetic and nondiabetic male patients
Source: Physiol Rep. 2025 Aug 11;13(15):e70509. doi: 10.14814/phy2.70509 (PMC12339417; doi:10.14814/phy2.70509)
Supplement: Supplementary file 1 — Appendix S1. [file PHY2-13-e70509-s002.pdf]

Methods

Human Right Atrial Appendage Tissue Acquisition

Auckland City Hospital Research Nurses in the Cardiovascular Intensive Care Unit carried out the patient consenting process following the processes stipulated by our ethical approvals. Patients scheduled to receive coronary artery bypass graft (CABG) surgery were recruited based on the exclusionary criteria below. Consenting patients agreed to the donation of a small right atrial appendage (RAA) sample for research into diabetic heart disease at the University of Auckland.

Exclusion criteria:

1) Patients undergoing multiple procedures during their surgery
2) Patients with insufficient fluency in the English language to provide informed consent
3) Patients who have previously undergone cardiac surgery
4) Patients with a diagnosed cardiomyopathy or heart failure

Recruited patients had a piece of RAA tissue (~ 0.5 x 2 cm) excised from the border of the cannula incision site during the CABG surgery. The dissected tissue was immediately placed in Krebs-Henseleit (KH) buffer solution bubbled with carbogen (95% O_2 and 5% CO_2) and transported to our research laboratory at the University of Auckland, as previously described by Jones et al. (2023).

KH is a physiological buffer mimicking the ionic composition of the human extracellular environment in vivo (Krebs & Henseleit, 1932). It has been used as a standard bathing solution to prepare dissected human heart tissue for experiments (Mulieri et al., 1989). KH buffer was prepared in batches containing 118 mM NaCl, 4.75 mM KCl, 1.18 mM $MgSO_4.7H_2O$, 1.18 mM KH_2PO_4 and 24.8 mM $NaHCO_3$. On the day of tissue collection, 11 mM of glucose, 25 mM 2,3-butanedione monoxime (BDM), and 0.25 mM $CaCl_2$ were added to the buffer, and the buffer was bubbled with carbogen (95% O_2 and 5% CO_2). The addition of glucose to the buffer only during the day of tissue collection was to prevent bacterial growth. The addition of BDM suppressed cardiac muscle contraction to protect myocardium from irreversible damage due to contracture and energy and oxygen deprivation (Sellin & McArdle, 1994; Wiggins et al., 1980).

Immunohistochemistry (IHC)

This study immunolabelled collagen type I & III and vimentin (fibroblasts) in trabeculae sections using Alexa Fluor-conjugated mono-specific antibodies. Cardiomyocytes and cell nuclei were labelled with Alexa Fluor-conjugated phalloidin and 4′,6-diamidino-2-phenylindole (DAPI), respectively. Table S1a summarises the antibodies and fluorescent probes used in the study. Longitudinal and transverse sections of trabeculae were labelled in two sequential incubations across two days, using different combinations of antibodies and fluorescent probes, summarised in Table S1b. All immunolabelling was carried out at room temperature under controlled humidity unless specified otherwise. For primary antibody labelling, selected tissue slides were retrieved from the -20°C freezer and placed in an incubation chamber. Sections were first rehydrated in phosphate-buffered saline (PBS) for 5 mins, an isotonic buffer solution with a pH of 7.4 containing NaCl, KCl, KH_2PO_4 and Na_2HPO_4. Rehydrated sections were then blocked with normal goat serum for 1h (Thermo Fisher Scientific, USA). After primary blocking, sections were washed in 20 µL of PBS three times for a total of 15 mins and additionally blocked with Image-iT® FX Signal Enhancer for 1h (Thermo Fisher Scientific, USA). At 30 mins before completion of blocking, a “cocktail” of primary antibodies was prepared in 50 µL tissue incubation solution (1 % bovine serum albumin, 0.05 % sodium azide and 0.05 % Triton X-100 PBS). Sections were blot-dried after blocking and then incubated in the diluted primary antibodies at 4 °C overnight.

For secondary labelling, primary antibody-labelled sections were washed in 20 µL of PBS three times for a total of 1h. At 30 mins before the completion of washing, a “cocktail” of secondary antibodies and fluorescent probes was prepared in 50 µL of tissue incubation solution. Washed sections were blot-dried and incubated in diluted secondary antibodies for 2h at room temperature in the dark. Once completed, the sections were washed in PBS for 1h and blot-dried. 10 µL of Prolong Gold antifade reagent containing DAPI (Thermo Fisher Scientific, USA) was applied on top of the tissue and immediately mounted onto slides. The corners of the coverslips were sealed with nail polish and left to cure in the dark at room temperature for at least 48 hours. Cured slides were fully sealed off and stored at 4 °C until imaging.

Table S1a **The antibodies and fluorescent probes used in immunohistochemistry**

Antibody	Catalog #	Clone/Isotype	Manufacturer
Rabbit collagen I polyclonal Antibody	Ab34710	Rabbit IgG	Abcam
Mouse collagen III monoclonal Antibody	Ab6310	Mouse IgG1	Abcam
Alexa Fluor 647 conjugated Vimentin monoclonal antibody	MA5-11883-A647	V9/Mouse IgG1	Thermo Fisher Scientific, USA
Alexa Fluor 647 conjugated anti-mouse antibody	A21235	Goat IgG	Thermo Fisher Scientific, USA
Alexa Fluor 488 conjugated anti-rabbit antibody	A11008	Goat IgG	Thermo Fisher Scientific, USA
Alexa Fluor 594 conjugated Phalloidin	A12381	-	Thermo Fisher Scientific, USA
DNA stain DAPI in Prolong Gold mountant	P36935	-	Thermo Fisher Scientific, USA

Table S1b. Applications of antibodies and fluorescent probes in labelling longitudinal and transverse sections of trabeculae

**Trabeculae longitudinal sections**	**Type I collagen**	**Type III collagen**	**Myofilament**	**Nuclei**
**Primary incubation**	Rabbit anti-collagen I antibody (1:100)	Mouse anti-collagen III antibody (1:400)		
**Secondary incubation**	Anti-rabbit Alexa Fluor 488 antibody (1:100)	Anti-mouse Alexa Fluor 647 antibody (1:100)	Alexa Fluor 594 conjugated Phalloidin (1:100)	DAPI (Prolong Gold antifade)
**Trabeculae transverse sections**	**Type I collagen**	**Fibroblast**	**Myofilament**	**Nuclei**
**Primary incubation**	Rabbit anti-collagen I antibody (1:50)	Mouse Alexa Fluor 647 conjugated anti-vimentin antibody (1:400)		
**Secondary incubation**	Anti-rabbit Alexa Fluor 488 antibody (1:100)		Alexa Fluor 594 conjugated Phalloidin (1:100)	DAPI (Prolong Gold antifade mountant)

Tile Imaging

The "Tile-scan" function allows a large tissue area to be captured in high resolution by taking multiple small images and "stitching" them together to create one large image. In ZEN, a preview scan was first taken using a 10 x air objective (NA = 0.45) to locate the area of tissue on the slide. A region of interest (ROI) was freehand drawn on the preview images, and ZEN automatically distributed tiles to cover the tissue area with a 10% overlapping area between the tiles. To calibrate the 2D focal plane of the tiles, support points were evenly distributed across the tissue area, and the "Auto-focus" function was used to find the brightest plane at each support point that was considered the optimal plane.

Once the placement of the tiles and focal plane calibration was completed, laser light of 405, 488, 561, and 640 nm was used to excite DAPI, Alexa Fluor 488, 594, and 647 fluorophores in the tissue sections, respectively. Emitted light was collected within the corresponding wavelength ranges of 400-496, 496-584, 595-637, and 655-700 nm. Multiple high-resolution 2D fluorescent images (~ 30-130) were sequentially captured by the detector. All images were captured in 16-bit with pixel sizes of 0.07 µm or 0.09 µm, depending on which 63x and 40x magnification was used. The image frame size was automatically optimised by ZEN before imaging, which met the minimal requirement of Nyquist sampling. Finally, all captured images were stitched together using the "Stitching" processing function in ZEN and imported into ImageJ Fiji.

Trabeculae Diameter, Longitudinal and Transverse Sectional Area, and Sarcomere length

Before quantifying the area of each label, the diameter of each trabecula was measured from the images to investigate whether there was any difference in trabeculae size between the ND and T2D groups. As shown in Figure S1a, trabeculae diameter was measured freehand from the trabeculae longitudinal section. Five measurements were taken from each trabeculae section and averaged to obtain the diameter for that section. Meanwhile, it was noticeable that trabeculae contained two layers: the myocardium, rich in myofilaments, and the surrounding endocardium, which contained predominantly collagen (Figure S1a). Each tissue sample had a different proportion of the myo- and endocardium dependent on the trabeculae diameter. Thus, the diameter of the myocardium was freehand measured, as shown in Figure S1a. Regarding the endocardium, the "Local thickness" analysis was adapted from ImageJ Fiji to

measure the thickness of the endocardium. This analysis is based on the algorithm first proposed by Saito and Toriwaki (1994) that computes the diameter of the largest sphere that can fit inside the object (endocardium). Figure S1b shows a stepwise process of carrying out the “Local thickness” analysis to measure the trabeculae endocardial thickness.

Furthermore, longitudinal and cross-sectional area was measured from the trabeculae tissue sections and used for composition analyses. As shown in Figure S1c, the longitudinal area of the trabeculae was determined by tracing the boundary of type I collagen labelling. The image channel of type I collagen was duplicated, and global thresholding was applied using a self-determined threshold to isolate the pixels. Pixel clusters with intensity equal to or above the threshold were converted to binary format and transformed into a binary image. The hollow regions within the signal area were filled, and the area was measured to obtain the longitudinal area of the trabeculae. Following the same protocol, the cross-sectional area of the trabeculae was obtained from images that capture the transverse section of the trabeculae, as shown in Figure S1d. Since trabeculae myo- and endocardium have different compositions, these two layers were isolated from the images and their area and composition were quantified independently. To isolate the myocardium from the images, global thresholding was applied to the image channel of phalloidin to create binary masks that represent the myocardium. As shown in Figure S1e, by subtracting the myocardium from the entire tissue area obtained from the previous analysis (see Figure S1c), the remaining area represents the endocardium.

Lastly, trabeculae sarcomere length was measured from the longitudinal sections using Fourier analysis and compared between groups (Cooley & Tukey, 1965). A stepwise process of performing Fourier analysis is shown in Figure S1f. First, the longitudinal section of a cardiomyocyte with a clear periodic structure was freehand isolated from the image. Fast Fourier Transform (FFT) was applied to transform the image into a Fourier image (frequency domain). Thresholding was applied to the Fourier image with a self-determined threshold to exclude all pixel clusters except for the cluster at the centre and the two adjacent clusters. Reverse FFT was then applied to transform the Fourier image back into the confocal image (spatial domain). The application of FFT to the image was to identify all repeating structures in the cardiomyocytes and transform them into the frequency domain. Thresholding applied to the Fourier image was to filter out all repeating structures in the cardiomyocytes except for the one with the highest frequency, presumably the Z-lines (Figure S1f). Finally, the application of reverse FFT was to return the image from the frequency to the spatial domain. The intensity

plot of the image was obtained, and the peaks in the plot profile were assumed to represent the signals of Z-lines in the sarcomere (Figure S1f). The spacing between the peaks along the x-axis was measured and averaged to obtain the mean sarcomere length in the selected cardiomyocyte. Sarcomere length was measured from five randomly selected cardiomyocytes from every tissue section and averaged to obtain the sarcomere length for that section.

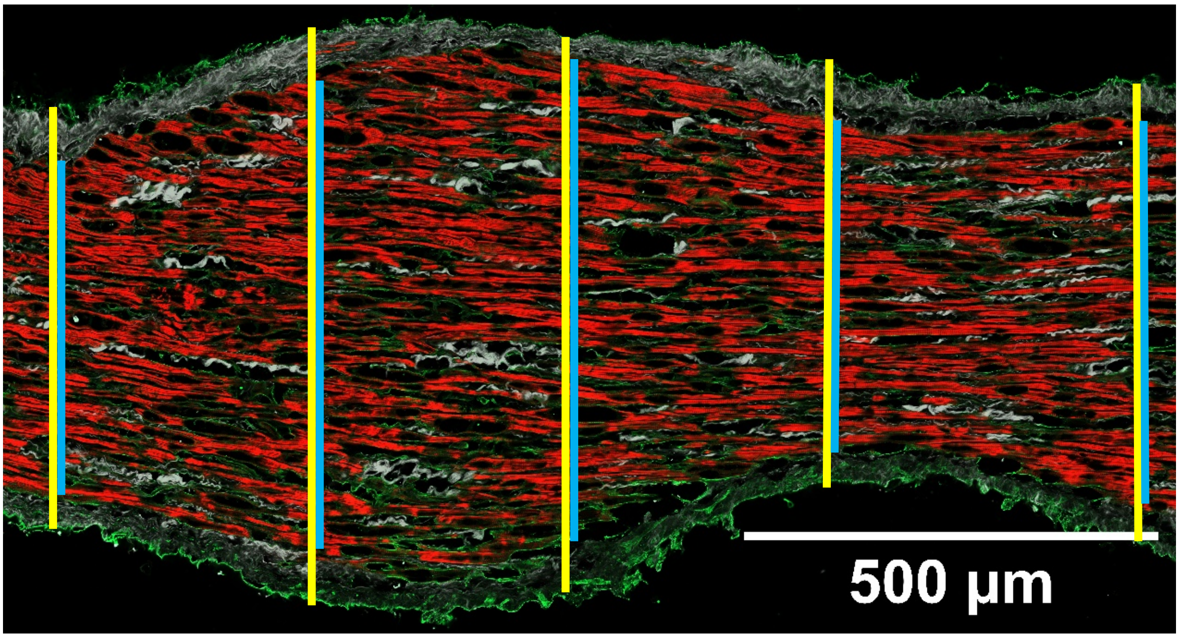

Figure S1a Trabeculae diameter measurement. The confocal image shows an example trabecula from a longitudinal section labelled by phalloidin (red), type I collagen antibodies (green), and type III collagen antibodies (grey). Yellow lines were drawn between the border of the trabecula, perpendicular to the long axis of the muscle. Blue lines were drawn between the border of the phalloidin labelling parallel to the yellow lines. The length of the lines was measured to obtain the average diameter of the trabecula (yellow lines) and the myocardium (blue lines).

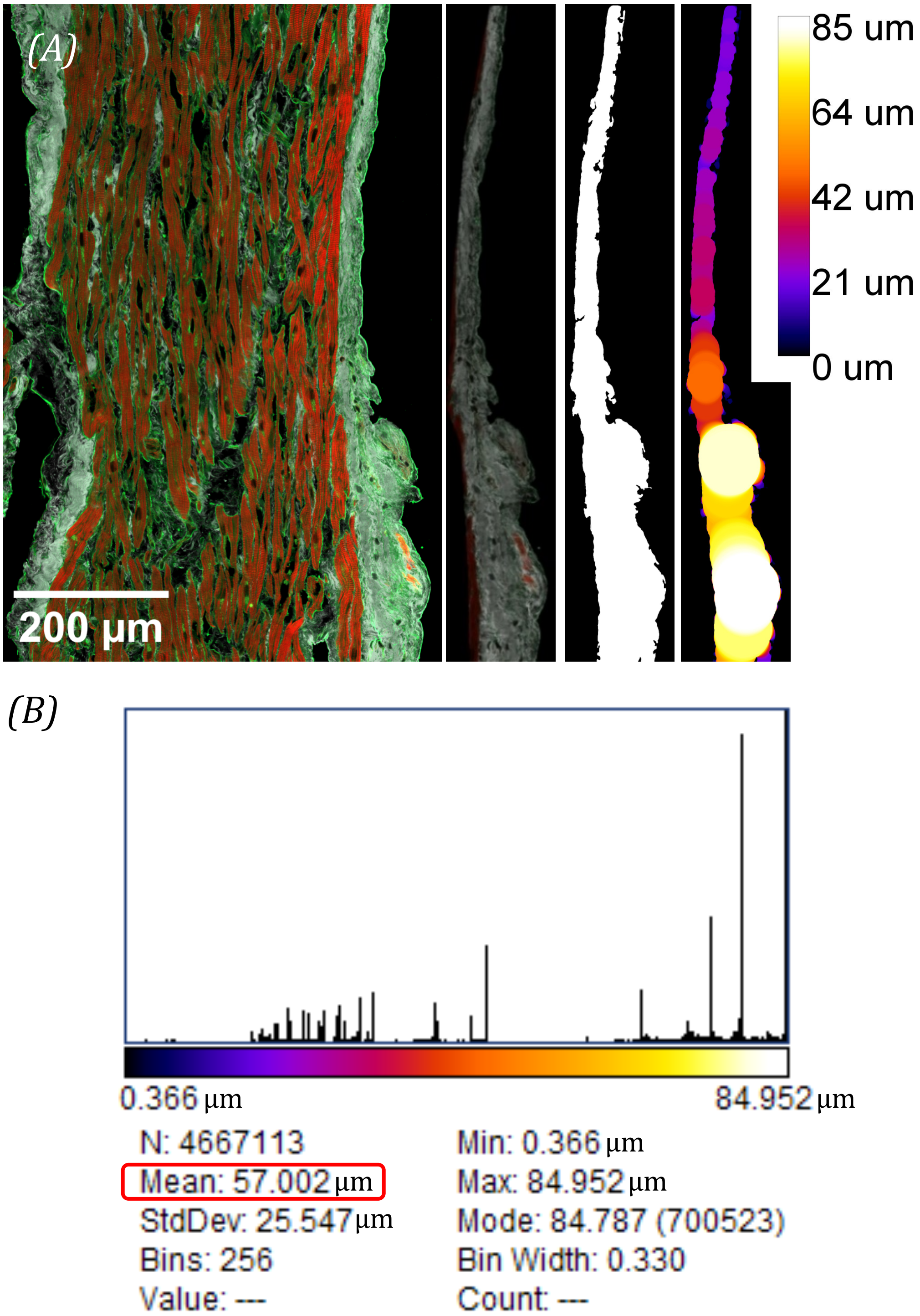

Figure S1b **Trabeculae endocardial thickness measurement.**

(A) demonstrates the method of obtaining the endocardial thickness of a representative trabecula from a longitudinal section. The endocardium of this trabecula was first manually cropped from the image, and global thresholding was applied to create a binary image. The "Local Thickness" function in ImageJ Fiji was applied to generate a "Local Thickness map". The brightness of the colour corresponds to the local thickness shown in the calibration bar. In the histogram shown in (B), the "Mean" value (red rectangle) is the calculated mean thickness of the endocardium of this trabecula.

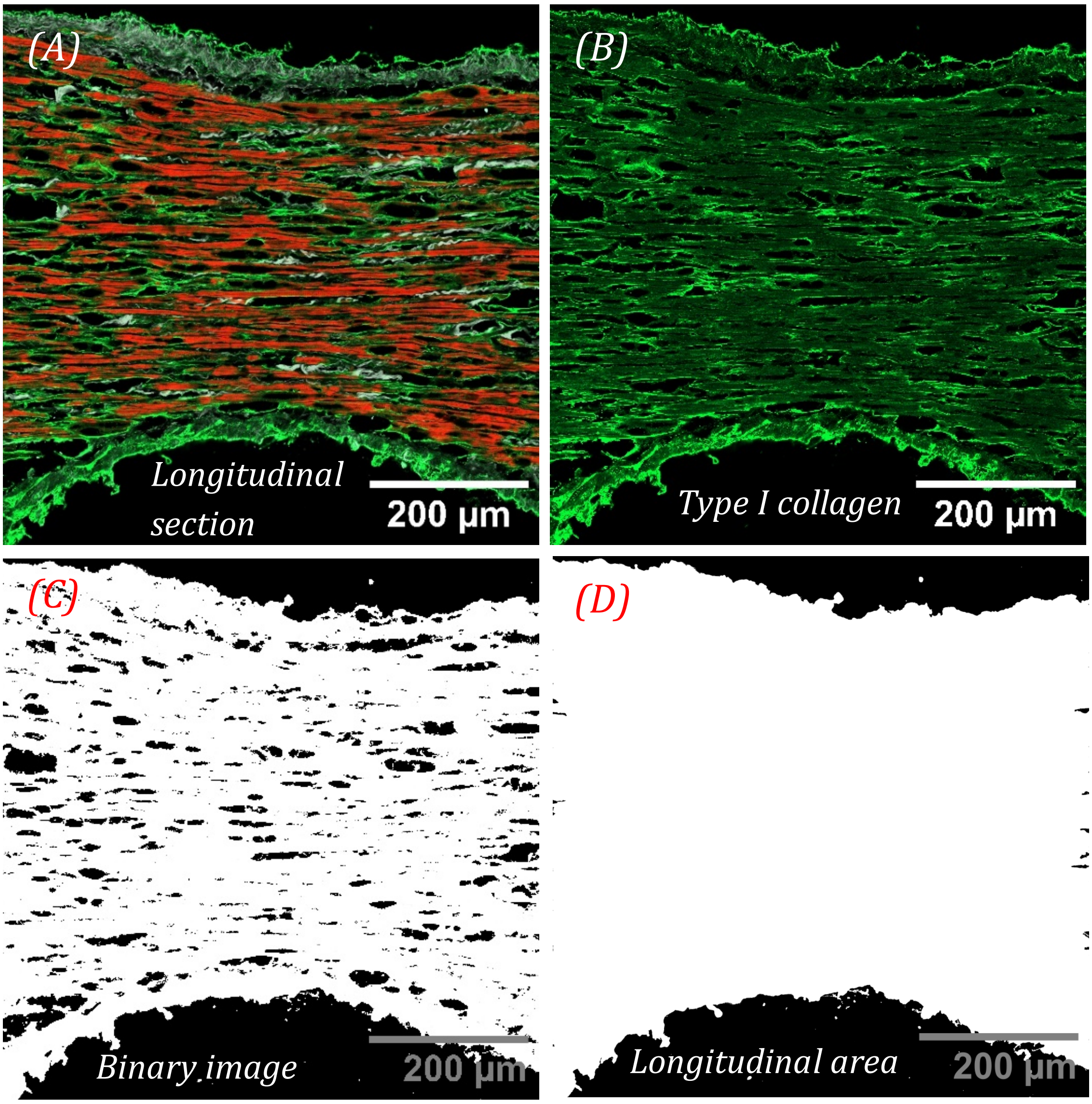

Figure S1c. **Trabeculae longitudinal area quantification.**

(A) shows an example trabecula from the longitudinal section labelled by phalloidin (red), type I collagen antibodies (green), and type III collagen antibodies (grey). (B) is the image channel of type I collagen isolated from (A). (C) is the binary mask generated by applying global thresholding to (B) with a self-determined threshold. (D) is (C) after filling holes in the tissue. The area of the mask in (D) (white pixels) was measured to obtain the tissue area in this section used for subsequent compositional analysis.

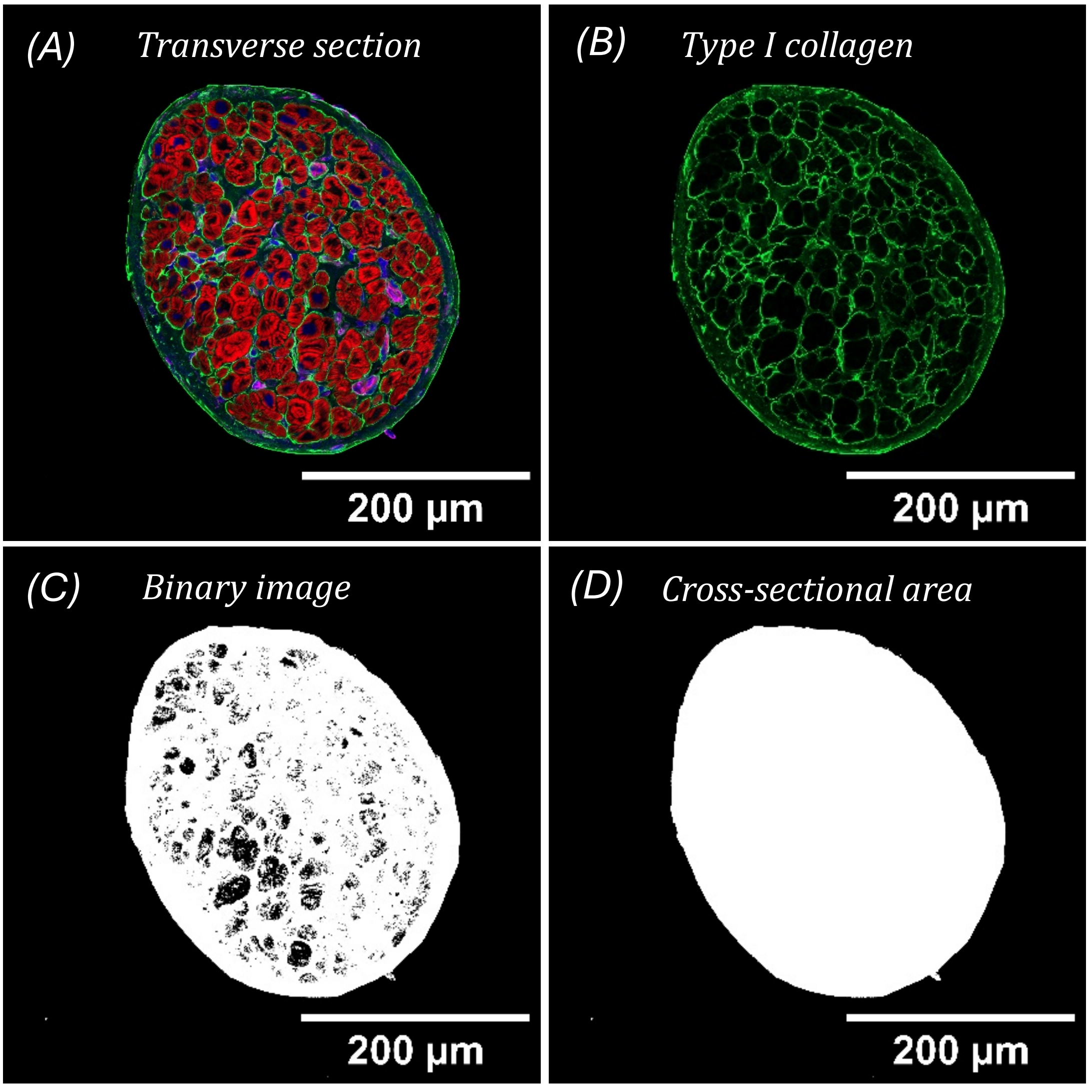

Figure S1d **Trabeculae cross-sectional area quantification.**

(A) shows an example trabecula in the transverse section labelled by phalloidin (red), type I collagen-antibodies (green), vimentin-antibodies (purple), and nuclei-marker (DAPI; blue). (B) shows the type I collagen image channel isolated from (A). (C) is the binary mask generated by applying global thresholding to (B) with a self-determined threshold. (D) is (C) after filling holes in the tissue area. The area of the mask in (D) (white pixels) was measured to obtain the cross-sectional area of this trabecula.

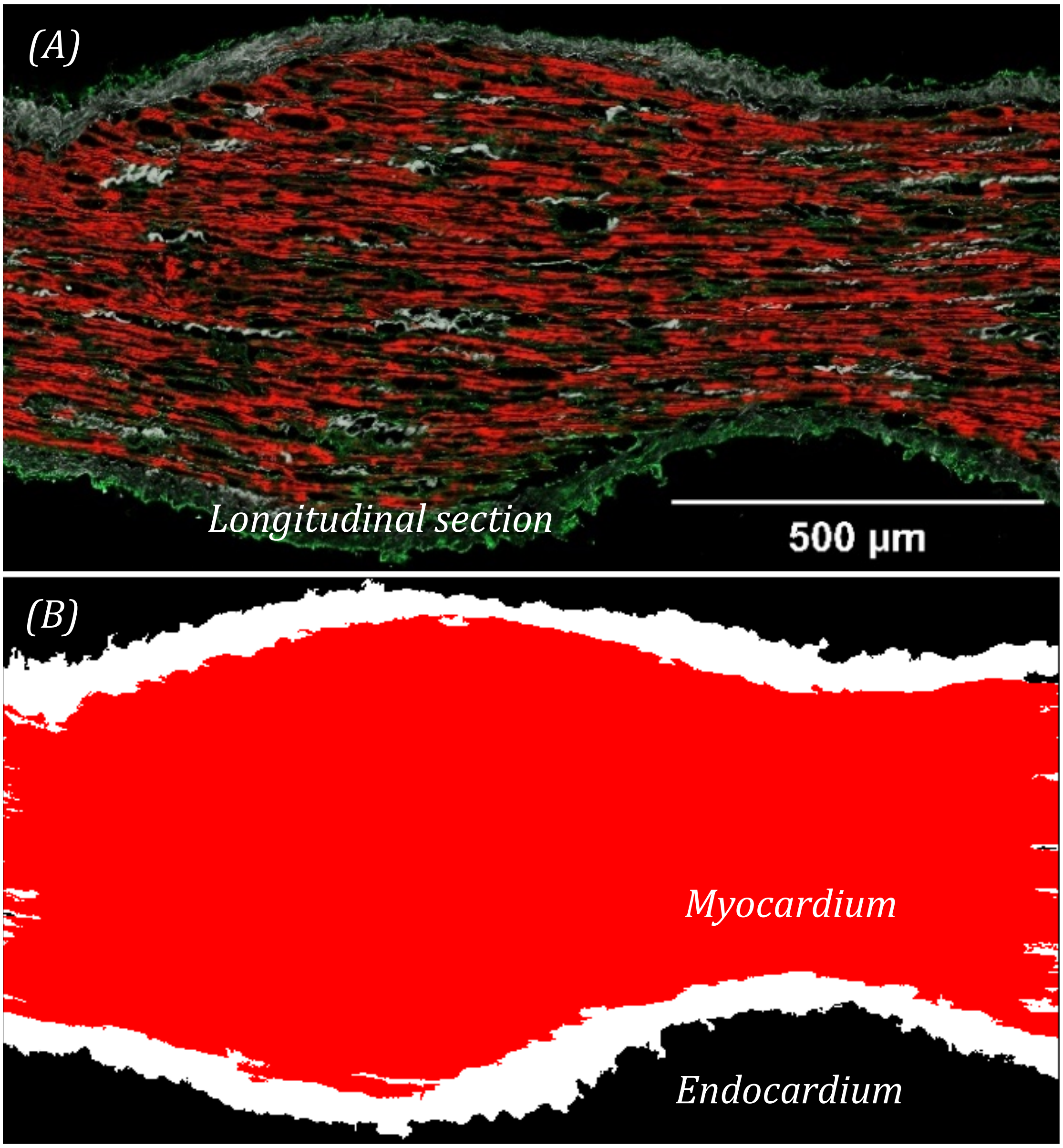

Figure S1e **Trabeculae myocardium and endocardium isolation.**

(A) shows an example trabecula from the longitudinal section labelled by phalloidin (red), type I collagen antibodies (green), and type III collagen antibodies (grey). (B) shows the method of isolating the myo- and endocardium by first applying thresholding to the image channel of phalloidin to obtain a binary mask of the myocardium (red pixels). The area of the myocardium was then deducted from the tissue area obtained previously (see Figure S1c) to obtain the area of the endocardium (white pixels).

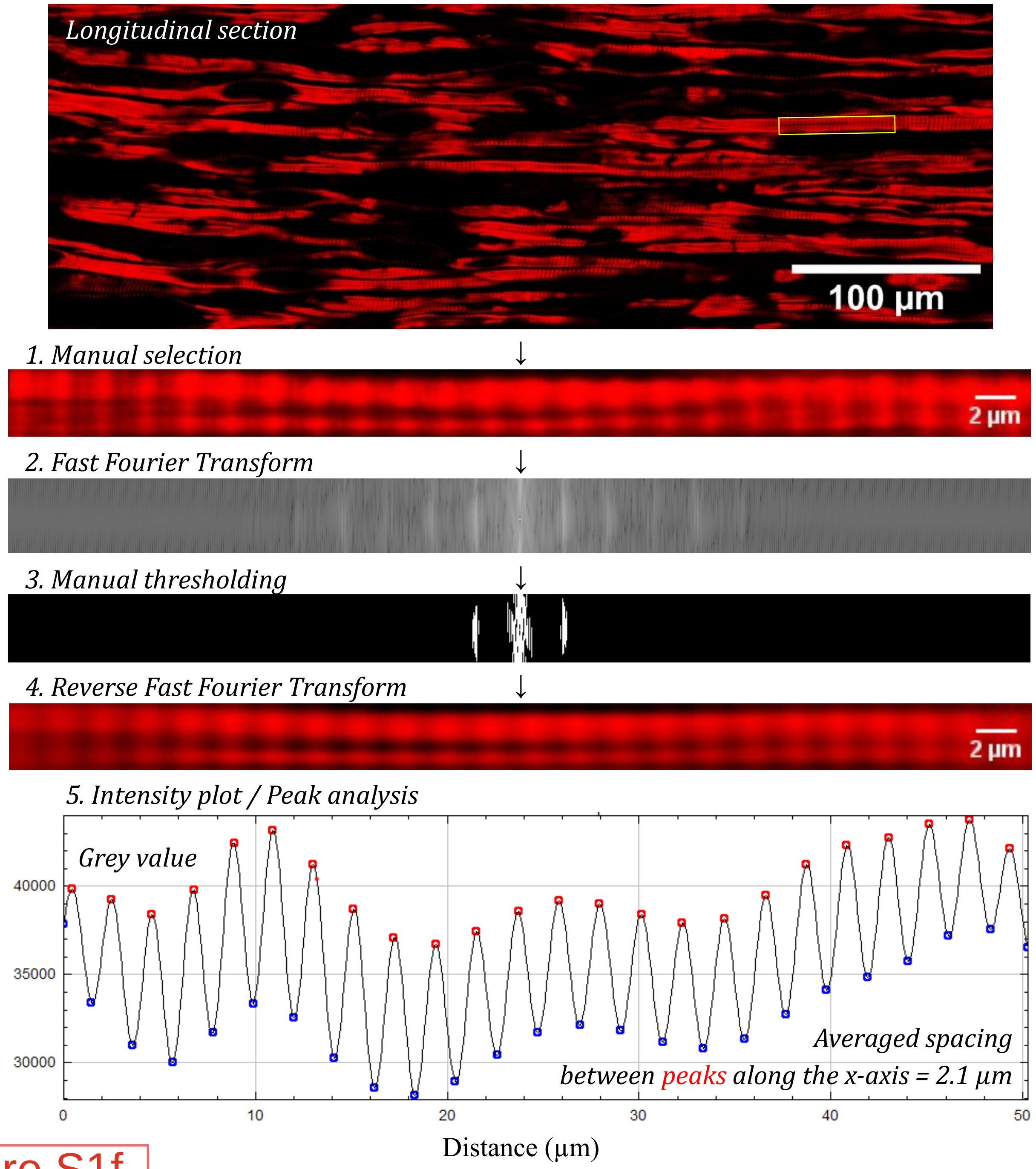

Figure S1f **Sarcomere length measurement.**

Figure shows the process of measuring sarcomere length from the longitudinal section of an example trabecula. Tissue section was labelled with the myofilament-marker phalloidin (red). (1) A section of cardiomyocyte with a clear periodic structure (yellow rectangle) was manually selected from the image. (2) Fast Fourier Transform (FFT) was applied to the selected section. (3) Manual thresholding was applied to the Fourier-transformed image to remove all pixel clusters except for the central and two adjacent bands. (4) Reverse FFT was applied to the binary image. (5) The plot profile of (4) was obtained, and "Find Peaks" function (adapted from A collection of Broadly Applicable Routines (BAR); ImageJ Fiji) was used to detect the peaks (red dots). The averaged spacing between the peaks along the x-axis was calculated to obtain the averaged sarcomere length.

Quantitative Analysis of Myofilaments and Extracellular Proteins in the Right Atrial Appendage Trabeculae

After quantifying the diameter, area, and sarcomere length of the RAA trabeculae, we proceed to quantify the relative abundance of proteins of interest in the tissue. Myofilaments, type I and type III collagen were quantified from the trabeculae longitudinal tissue sections. From the transverse sections, myofilaments, type I collagen, and vimentin were quantified. Type III collagen was not labelled and quantified from the transverse sections because confocal microscopy only permits imaging of four labels from every section. Vimentin was therefore substituted in place of type III collagen to quantify the fibroblasts. The relative abundance of the myofilaments was determined by the area of phalloidin labelling relative to the tissue area obtained from the previous analysis (see Figure S1c), and type I and type III collagen and vimentin were determined by the area of antibody labelling relative to the tissue area.

When optimising the image analysis process, global thresholding and different automated thresholding algorithms were tested. However, global thresholding showed limitations when the labels had an inconsistent intensity caused by either tilting of the tissue section, uneven staining, and/or the nature of the tissue structure. Figure S1g shows an example trabecula in the transverse section. The intensity of type I collagen labelling showed a gradient because the tissue section was tilted. Applying global thresholding was unable to generate a binary image that truly reflected the tissue distribution and density of type I collagen, and this was irrelevant to which thresholding algorithms were used. Thus, local thresholding was used alternatively, a more advanced thresholding technique that generates threshold values specific to smaller areas of the images to differentiate the labels from the background signal (Neerad et al., 2011). This method improved the consistency and accuracy of thresholding (Figure S1g).

To quantify the area of type I and III collagen and phalloidin labelling from the trabeculae longitudinal sections, the corresponding image channels were duplicated, converted from 16 to 8-bit, and median filtered (radius = 2 µm) to improve signal-to-noise ratio. “Local thresholding” was applied to images by using the algorithm of “Phanasalkar”, developed by Neerad et al. (2011), to generate the binary images shown in Figure S1h. The area of labels was measured from the binary images and normalised to the tissue area obtained previously (see Figure S1c) to determine the relative abundance of the labelled proteins in the tissue. As shown in Figure S1i, the same image analysis pipeline was applied to quantify the relative

abundance of type I collagen, vimentin, and myofilaments from the transverse sections of trabeculae.

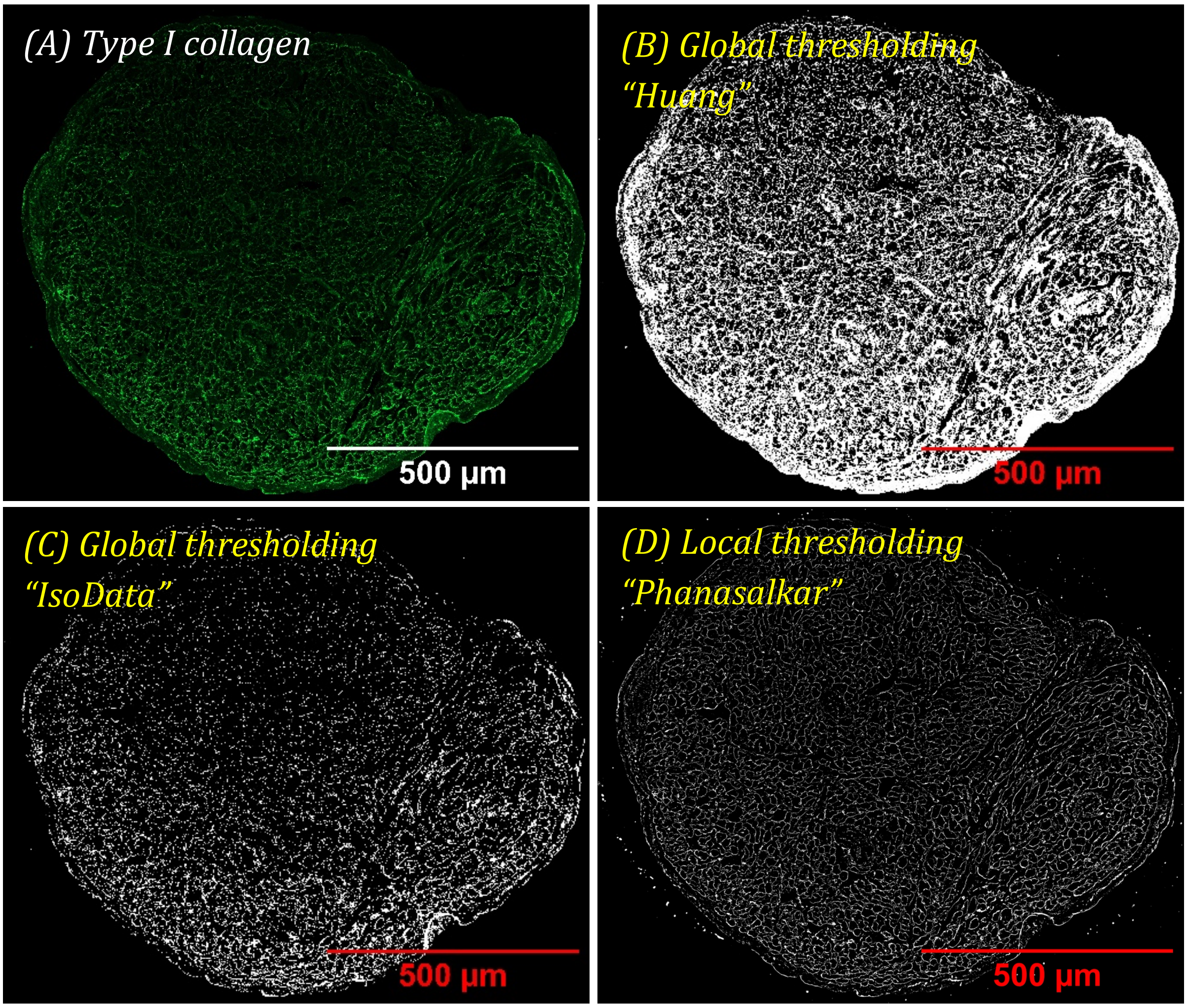

Figure S1g **Comparison of global and local thresholding.**

(A) shows the transverse section of an example trabecula labelled by type I collagen-antibodies (green). The intensity of the labelling shows a gradient that gradually decreases from the bottom right to the top left of the trabecula. The regional differences in density of type I collagen labelling were due to the tissue section positioned at the bottom right being higher than the top left. (B) shows the application of global thresholding on (A) using the algorithm of "Huang". The generated binary image exaggerated the labelling area of type I collagen at the bottom right. (C) shows the application of global thresholding on (A) using the algorithm of "IsoData". The binary image created from (C), however, failed to demonstrate all the type I collagen labelling at the top left of the tissue. (D) is the binary image created by applying local thresholding to (A) using the "Phanasalkar" algorithm. The signal area in (D) shows type I collagen labelling was evenly distributed, which better reflects the actual distribution of type I collagen in this tissue section.

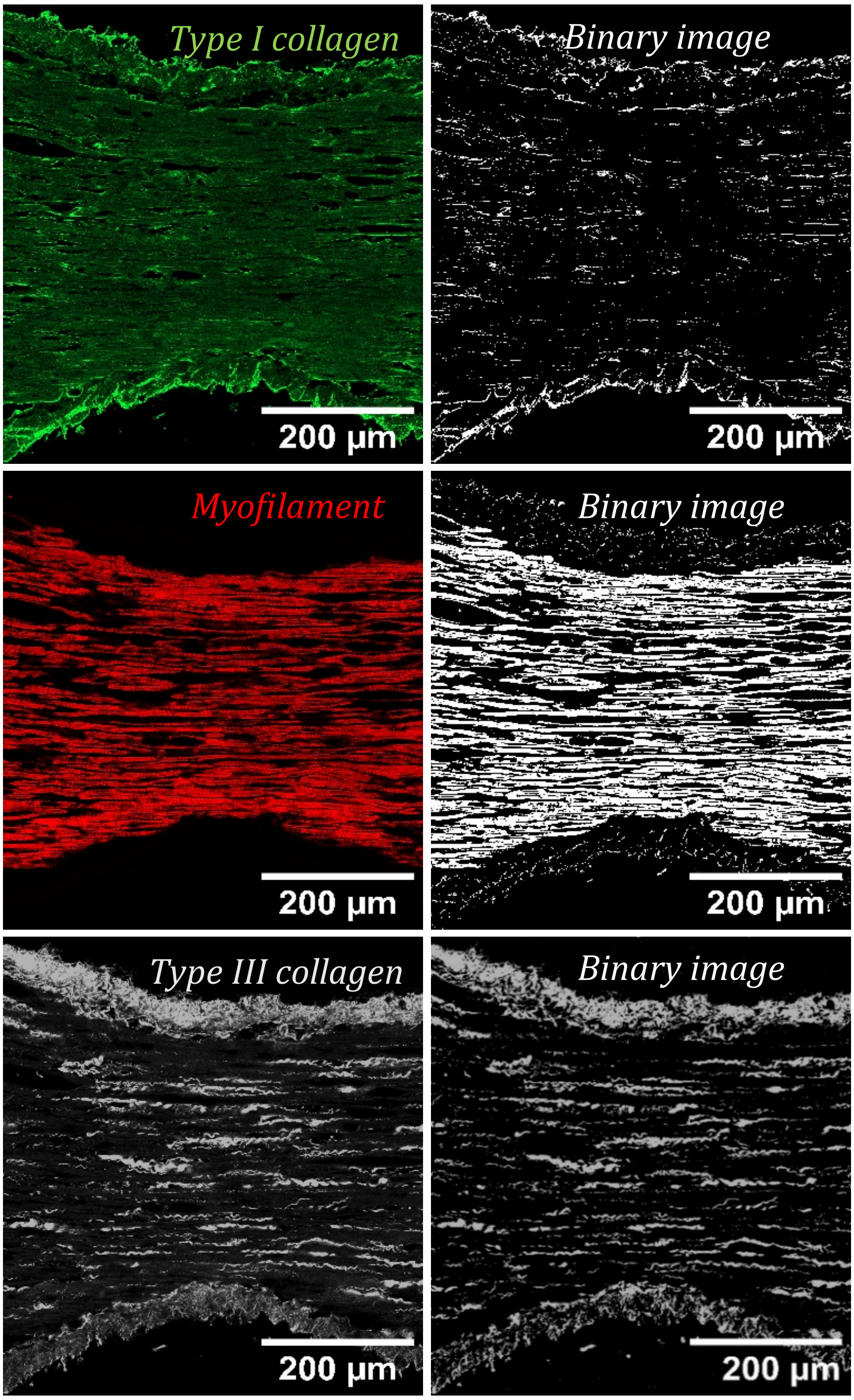

Figure S1h. **Quantitative analysis of type I collagen, type III collagen, and myofilaments from the longitudinal section of a trabecula.**

Images in the left column show an example trabecula in longitudinal section labelled by type I collagen-antibodies (green), phalloidin (red), and type III collagen-antibodies (grey). Images in the right column are the binary images created by applying local thresholding to the images in the left column using the algorithm of "Phanasalkar". The signal area in the binary images (white pixels) was measured to obtain the tissue area corresponding to the labelled proteins.

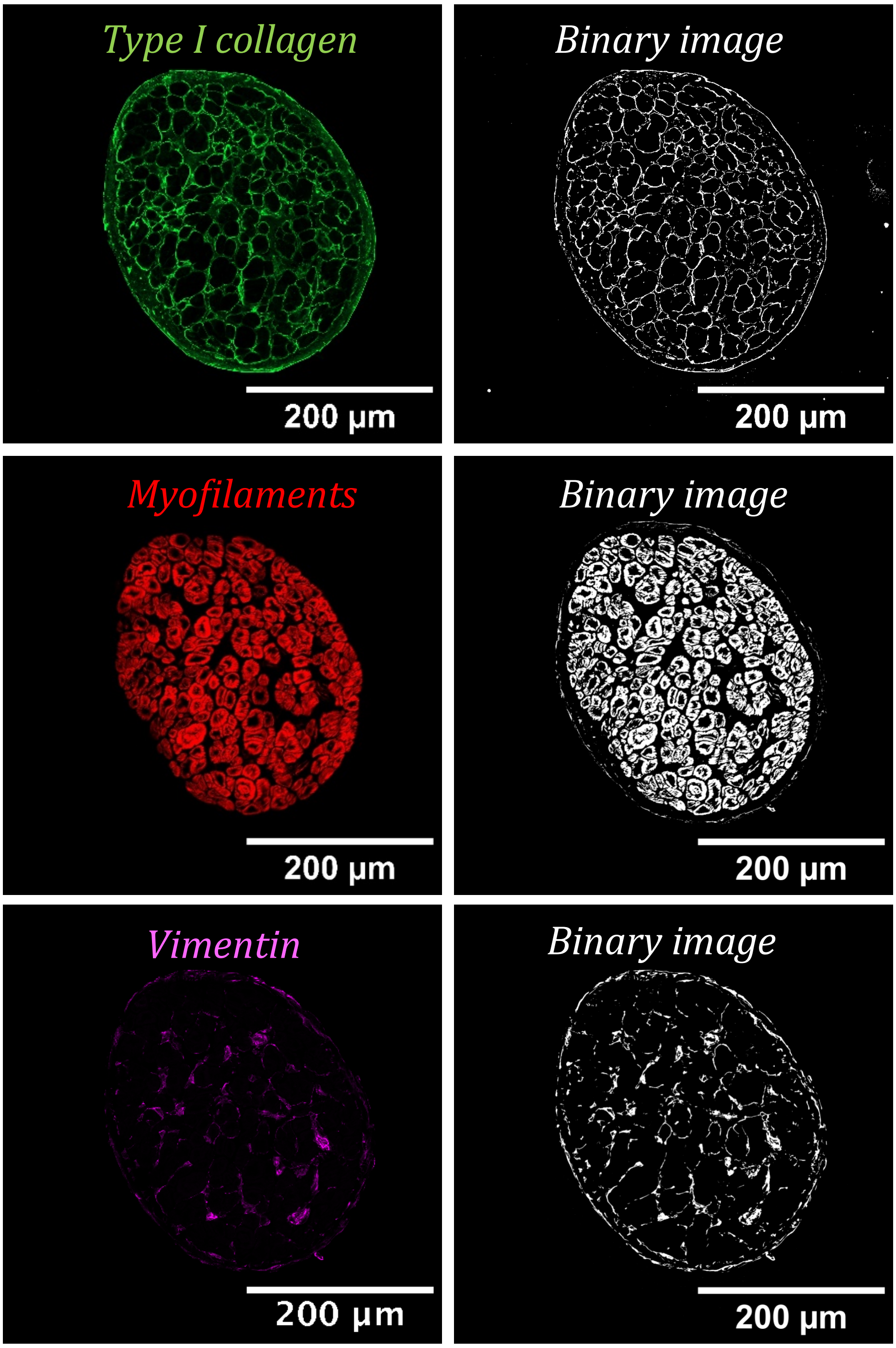

Figure S1i **Quantitative analysis of type I collagen, myofilaments, and vimentin from the transverse section of a trabecula.**

Images in the left column show an example trabecula from the transverse section labelled by type I collagen-antibodies (green), phalloidin (red), and vimentin-antibodies (purple). Images in the right column are the binary images created by applying local thresholding to the images on the left column using the algorithm of “Phanasalkar”. The signal area in the binary images (white pixels) was measured to obtain the tissue area corresponding to the labelled proteins.

Quantitative Analysis of Cardiomyocytes and Fibroblasts in the Right Atrial Appendage Trabeculae

Cardiomyocytes and fibroblasts are two distinct groups of cells in the RAA myocardium. Cardiomyocytes take up the most myocardial volume, and fibroblasts are more abundant in terms of number (Litviňuková et al., 2020). One of the objectives of this study was to investigate whether T2D induced changes in the cellular composition of trabeculae, and this was achieved by quantifying the relative occurrence of cardiomyocytes and fibroblasts in the cell population. Additionally, this study examined the changes in tissue density and morphology of these two groups of cells under the influence of T2D. To automate these analysis processes, several image-processing pipelines were developed to analyse the images that perform a series of commands in ImageJ Fiji (macro). The following sections describe the methods used to quantify cell nuclei and identify cardiomyocytes and fibroblasts from the transverse sections of RAA trabeculae.

Cell Nuclei Quantification

The primary step in quantifying cellular composition was to estimate the total number of cells in the tissue from the nuclei labelling (DAPI). Figure S1j shows the process of quantifying cell nuclei from an example trabecula. The image channel of DAPI was filtered by "Gaussian Blur" (sigma = 2), and global thresholding was applied to produce a binary image of DAPI labelling using the algorithm of "MaxEntropy" (Kapur et al., 1985). Every isolated signal cluster in the binary image within a minimal area of 2 μm^2 was identified as one nucleus and counted using the "Analyze Particles" function (ImageJ Fiji).

After obtaining the total number of nuclei, a custom-written macro was used to isolate and count the nuclei from cardiomyocytes. As shown in Figure S1k, the binary image of myofilament was adapted from the previous analysis (see Figure S1i) and overlaid on the binary image of nuclei (see Figure S1j). "Morphological reconstruction" analysis was adapted from an open-source plugin in ImageJ Fiji named "MorpholibJ" (Legland et al., 2016) and used to analyse the stacked image for isolating nuclei that co-localised with phalloidin labelling. The isolated nuclei were identified as cardiomyocyte nuclei, counted, and normalised to the total number of nuclei obtained previously (see Figure S1j) to determine the cardiomyocyte's occurrence in the cell population. Similarly, the fibroblast's occurrence in the cell population was quantified from the transverse sections using the same macro, but instead,

the fibroblast nuclei were identified from their co-localisation with type I collagen and vimentin labelling, as shown in Figure S1l.

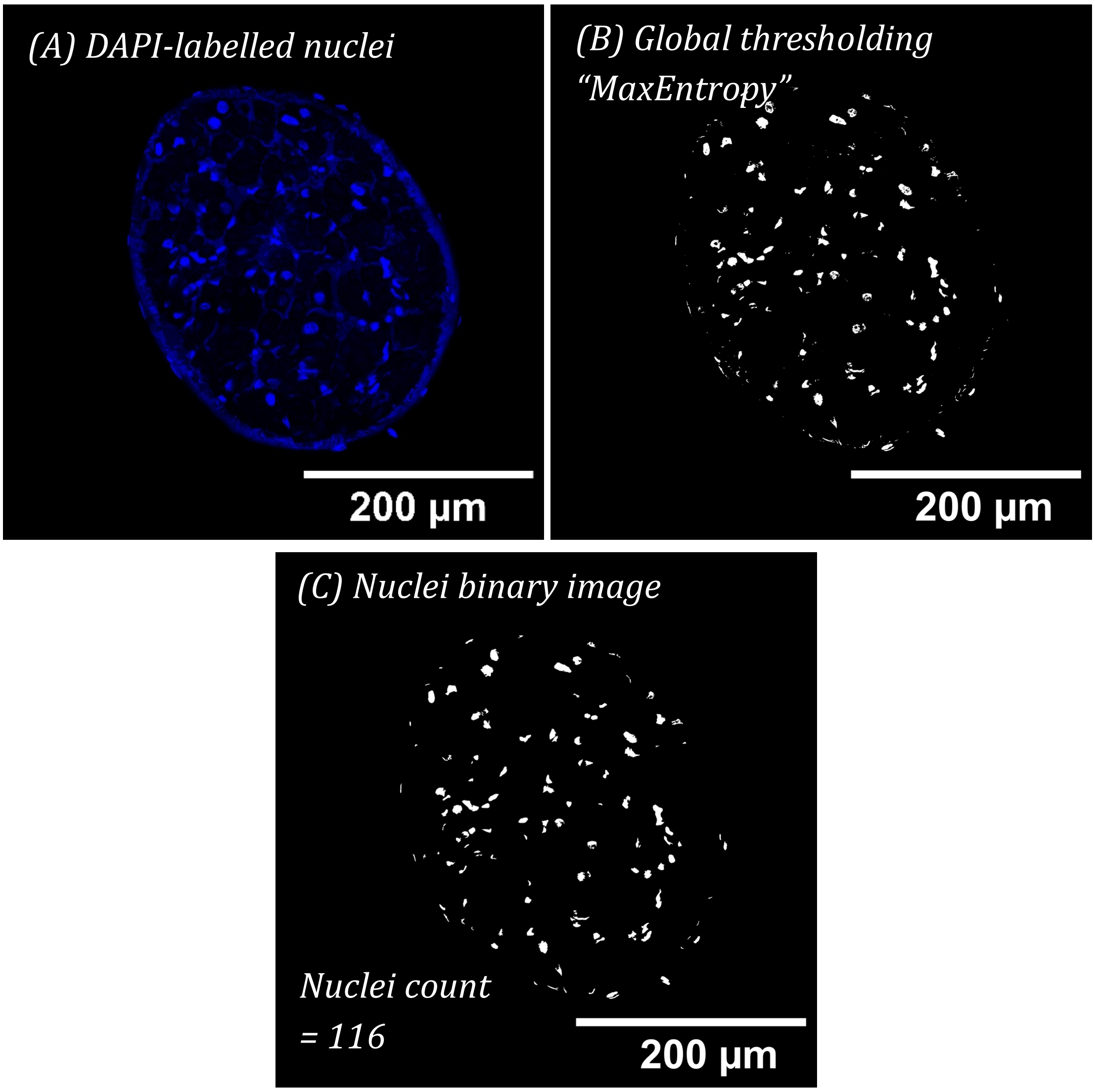

Figure S1j. Cell nuclei quantification

(A) is a confocal image of an example trabecula labelled by nuclei-marker (DAPI; blue). (B) is the binary mark created by applying global thresholding to (A) using the algorithm of "MaxEntropy". The signal clusters in (B) with a minimal area of 2 μm^2 were isolated using the "Analyze Particles" function. (C) is the resulting binary image, and the isolated signal clusters in the image were identified as nuclei and counted to obtain the total number of nuclei estimated from this section.

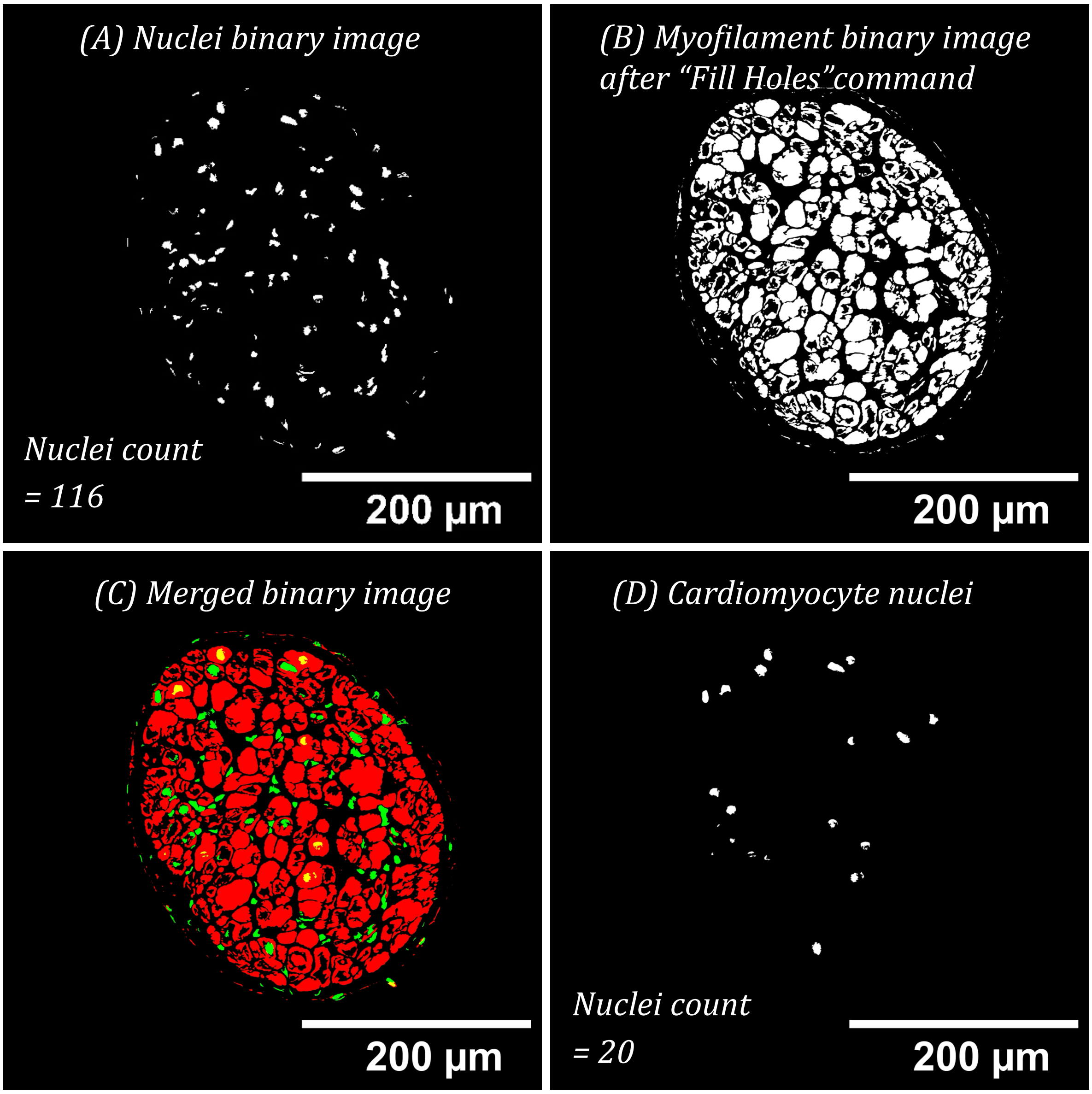

Figure S1k. Quantitative analysis of cardiomyocyte nuclei from the transverse section of a trabecula.

(A) is the binary image of nuclei copied from Figure S1j. (B) is the binary image adapted from Figure S1i. (C) is the merged image of (A) and (B), with phalloidin labelling shown in red and DAPI labelling shown in green. (D) shows the cardiomyocyte nuclei isolated from (C) by applying "morphological reconstruction", which identified nuclei that were co-localised with phalloidin labelling (myofilaments).

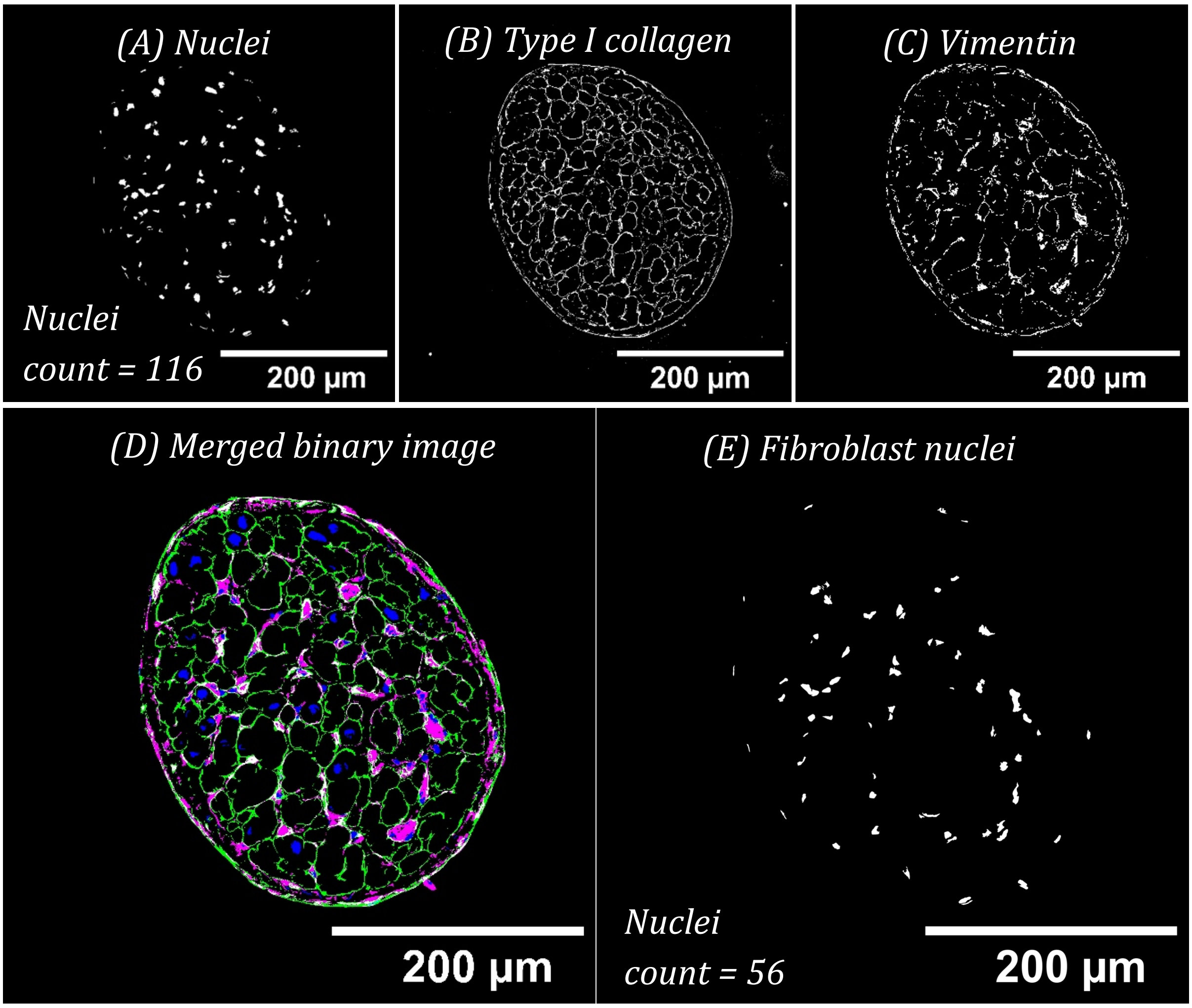

Figure S1l. Quantitative analysis of fibroblast nuclei from the transverse section of a trabecula.

(A) is the binary image of nuclei from Figure S1j). (B) and (C) are the binary images of type I collagen and vimentin from Figure S1i. (D) is the merged image of (A), (B), and (C), showing the co-localisation of nuclei (blue), type I collagen (green), and vimentin (purple). (E) shows nuclei isolated from (D) by applying “Morphological reconstruction” to identify nuclei that co-localised with both vimentin and type I collagen. The nuclei shown in (E) were counted to obtain the number of fibroblast nuclei in this trabecula from the transverse section.

Cardiomyocyte Identification and Quantification

Cardiomyocytes make up a small percentage of the myocardial cell population in number but occupy the majority of the myocardial space. The number of myofilament clusters was manually counted from the trabeculae transverse sections to determine the relative abundance of cardiomyocytes. As shown in Figure S1m, a 200 μm diameter circle was drawn at the centre of a tissue section, and the number of myofilament clusters within the circle was manually counted. The number of clusters was normalised to the area of the circle to obtain an estimated tissue density of cardiomyocytes. Furthermore, 3 to 5 myofilament clusters with centrally located nuclei were manually selected across the tissue section, depending on the availability, and used for subsequent morphological analysis.

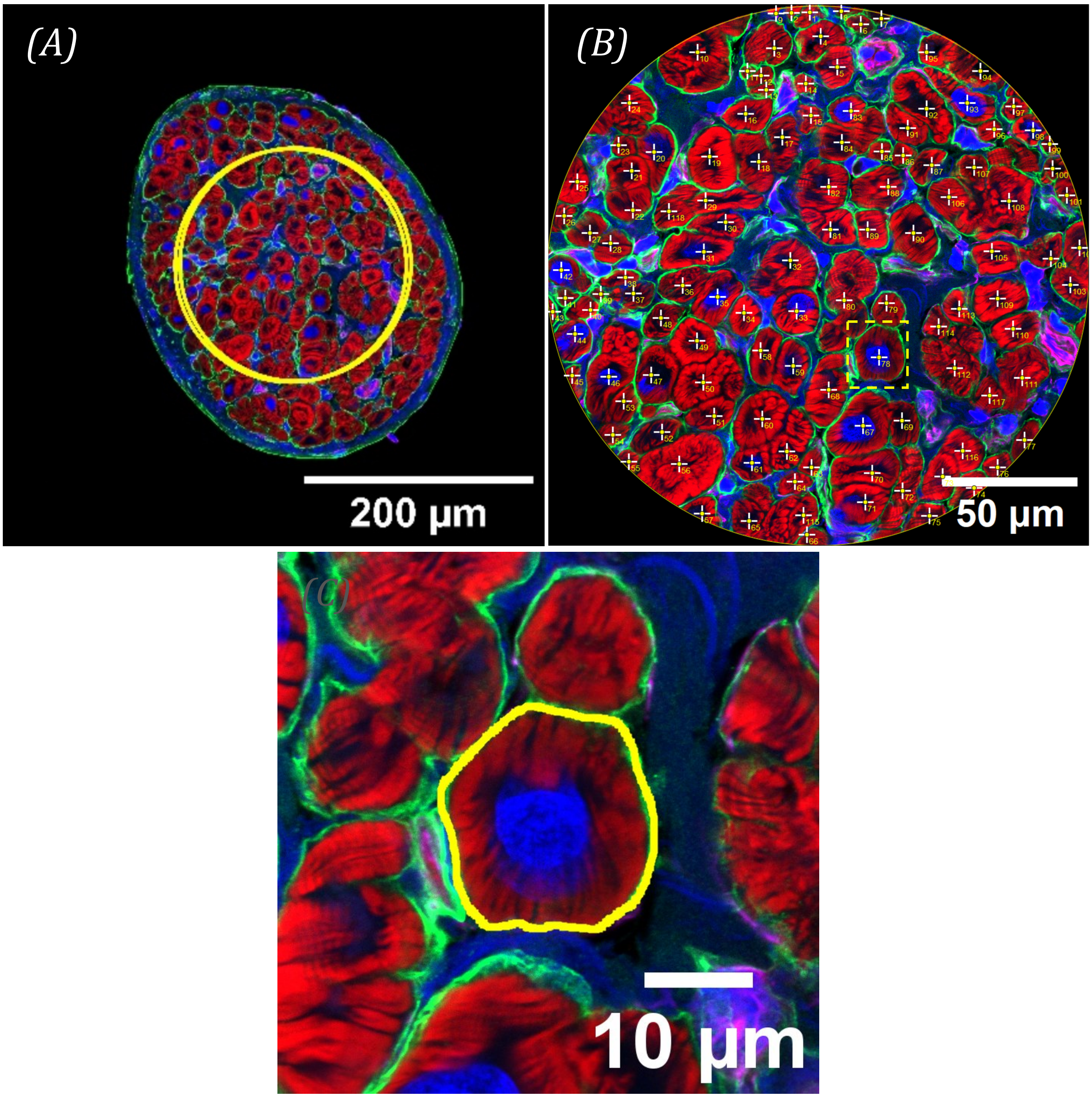

Figure S1m **. Cardiomyocyte quantification and manual selection.**

(A) shows an example trabecula in transverse section labelled with phalloidin (red), type I collagen-antibodies (green), vimentin-antibodies (purple), and nuclei-marker (DAPI; blue). (B) shows an enlarged area of (A) within the yellow circle. Every cross indicates a manually selected single cluster of phalloidin-labelled myofilaments identified as one cardiomyocyte. (C) shows a cardiomyocyte outlined by freehand used for subsequent morphological analysis, including cross-sectional area, diameter, perimeter, and circularity.

Fibroblast Identification and Quantification

Fibroblasts are much smaller cells than cardiomyocytes, although more abundant, making manual quantification a difficult task. Therefore, a custom-written macro was created to identify and quantify fibroblasts from trabeculae transverse sections. As shown in Figure S1-14, the binary images of vimentin and fibroblast nuclei obtained from the previous analysis (see Figure S1i & Figure S1l) were first merged, and "Morphological reconstruction" was used to segment the area of vimentin labelling co-localised with fibroblast nuclei. This allowed a binary image of vimentin labelling to be obtained, which was exclusive to the fibroblasts. Since vimentin is an intermediate filament protein associated with the cytoskeleton of fibroblasts, it is assumed that vimentin labelling within fibroblasts gives the overall shape of the fibroblasts, as previously described by Dulbecco et al. (1983). Segmented vimentin labelling was then added to the binary image of fibroblast nuclei to obtain a binary image identifying fibroblast cells. Additionally, fibroblasts can cross-link with the neighbouring fibroblasts via their filopodia to become a single entity. The "Marker-controlled Watershed" function adapted from "MorpholibJ" was therefore applied to separate the crosslinked fibroblasts, based on their nuclei (Meyer & Beucher, 1990). Finally, "Analyze Particles" (ImageJ Fiji) was applied to identify and isolate fibroblasts for subsequent morphological analysis.

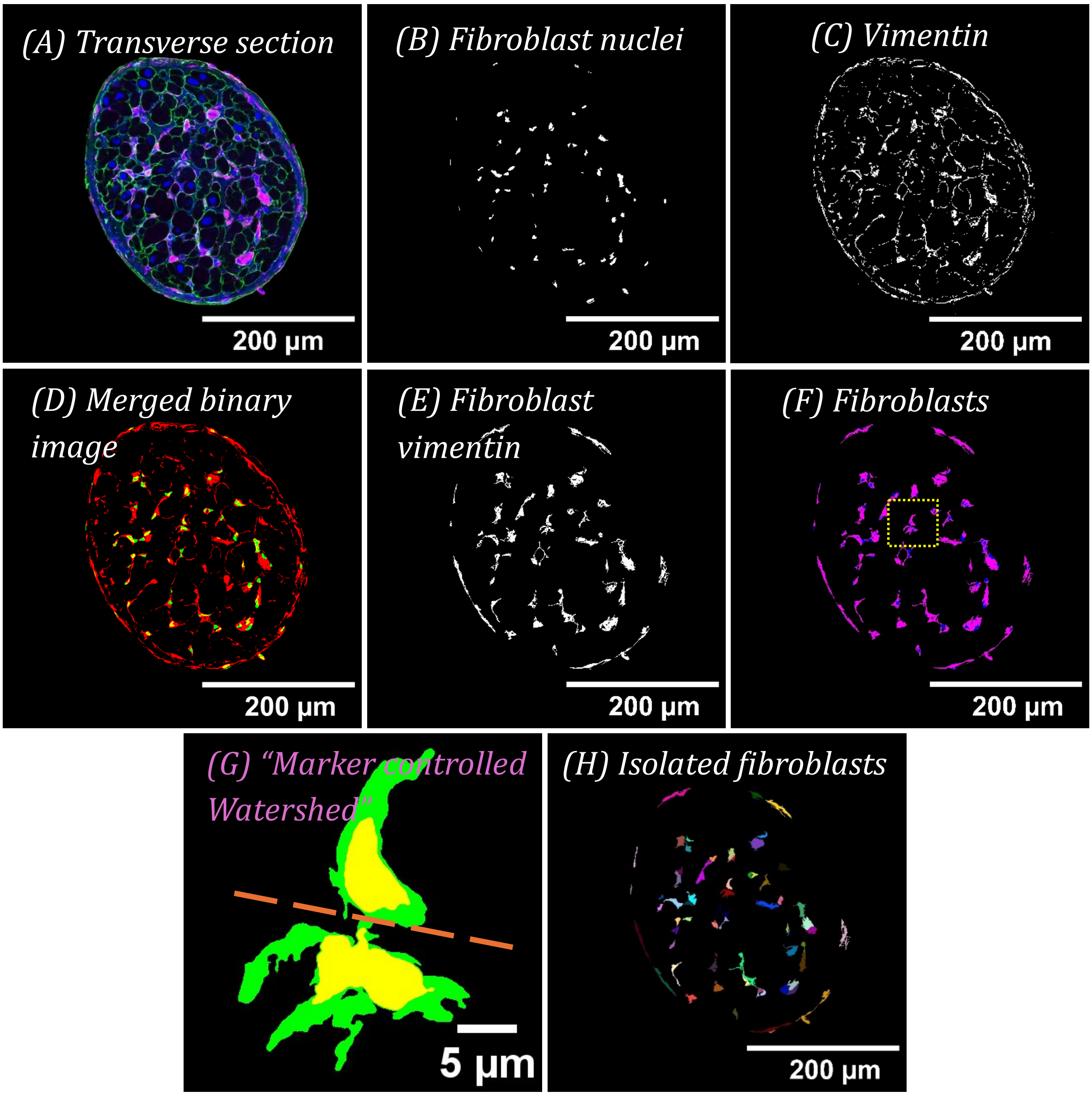

Figure S1n **Fibroblast identification and quantification.**

(A) shows an example trabecula transverse section labelled by type I collagen-antibodies (green), vimentin-antibodies (purple), and nuclei-marker (DAPI; blue). (B) shows the binary image of fibroblast nuclei copied fron Figure S1l [illegible]. (C) is the binary image of vimentin copied from Figure S1i . (D) shows the merged image of (B) and (C), with vimentin shown in red and nuclei shown in green. (E) is an image of fibroblast vimentin obtained by applying "Morphological reconstruction" to (D) to isolate vimentin labelling that was co-localised with fibroblast nuclei. (F) is the merged image of (B) and (E) showing the fibroblasts isolated from image (A). (G) shows the application of "Marker-controlled Watershed", which separates the two crosslinked fibroblasts into independent cells. (H) is the resulting binary image of fibroblasts after (A) was analysed using a custom-written macro. The different colours in (H) each indicate a single fibroblast.

Morphological Analysis of Cardiomyocytes and Fibroblasts

The morphology of cardiomyocytes and fibroblasts was described by parameters that include cross-sectional area, perimeter, Feret's minimal diameter, and circularity, with examples shown in Figure S1o. Feret's diameter, also called the "calliper diameter", describes the cell size in cross-section by measuring the maximal and minimal distance between two parallel lines tangential to the cell boundary (Feret, 1930). Meanwhile, the circularity of cardiomyocytes and fibroblasts was also determined (Chan et al., 2015; Hillsley et al., 2022). Circularity is a measure of how similar the cell shape is to a circle and is defined by the equation = 4π x ($area/perimeter^2$) on a scale from 0 to 1. A circularity of "1" indicates the cell has a perfect circular shape, and "0" indicates the cell is elongated. Additionally, the myofilament content of the cardiomyocytes was estimated by normalising the area of myofilament within the cardiomyocytes to the cross-sectional area of the cardiomyocyte.

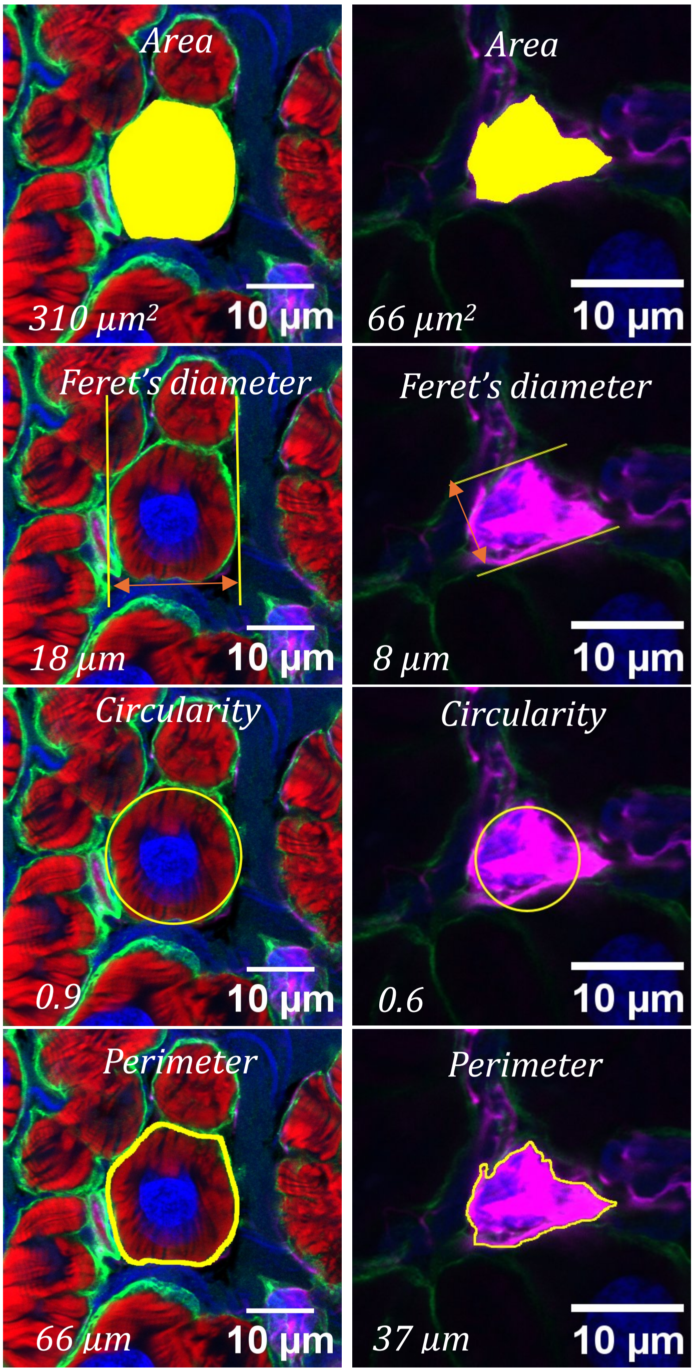

Figure S1o**. Morphological analysis of a cardiomyocyte and fibroblast.**

Confocal images on the left show a cardiomyocyte, and images on the right show a fibroblast. Both cells were identified from the transverse section of an example trabecula. The tissue section was labelled with phalloidin (red), vimentin-antibodies (purple), type I collagen-antibodies (green), and nuclei-marker (DAPI; blue). The cardiomyocyte identified has an area of 310 µm^2, minimum Feret's diameter of 18 µm, circularity of 0.9, and perimeter of 66 µm. The identified fibroblast has an area of 66 µm^2, minimum Feret's diameter of 8 µm, circularity of 0.6, and perimeter of 37 µm.

Measurement of Trabeculae Contractile Force

Measurements of trabeculae contractile force were carried out on the day of tissue collection. Trabeculae used in functional experiments were dissected from the same patient sample as those used for histology in this study. It is assumed that trabeculae from the same patient samples had comparable functional and structural characteristics, representative of the RAA tissue. A summary of the Methods used to obtain functional data is described below.

Following dissection, trabeculae were immediately transferred to a muscle chamber warmed to 37 ºC (model 801C, Aurora Scientific, Canada) and bathed in circulating oxygenated KH buffer containing 20 mM BDM and 0.5 mM Ca^2. One end of the trabecula was attached by a hook connected to a micromanipulator that allowed stretching and positioning of the trabecula. The other end of the trabecula was held in a wire hook connected to a force transducer (AE801, Kronex, Oakland, CA, USA). Figure S1p shows the experimental setup used for the measurement of contractile force in trabeculae.

After mounting, trabeculae were positioned between the stimulating electrodes connected to a stimulator (Radnoti REDSTIM multi-channel stimulator, ADInstruments, New Zealand). The circulating buffer was replaced with a BDM-free KH buffer, and the [Ca^{2+}] was increased to 1.5 mM. Trabeculae were electrically stimulated at a frequency of 0.5 Hz, and the stimulus voltage incrementally increased until a force response was obtained. The final stimulus voltage was then set at 20% above the threshold voltage. Once the muscle reached steady-state with each twitch of the same amplitude, the stimulation frequency was adjusted to 1 Hz. Trabeculae were then incrementally lengthened until the active force produced no longer increased with increasing length (optimal length; L_o) (Figure S1q).

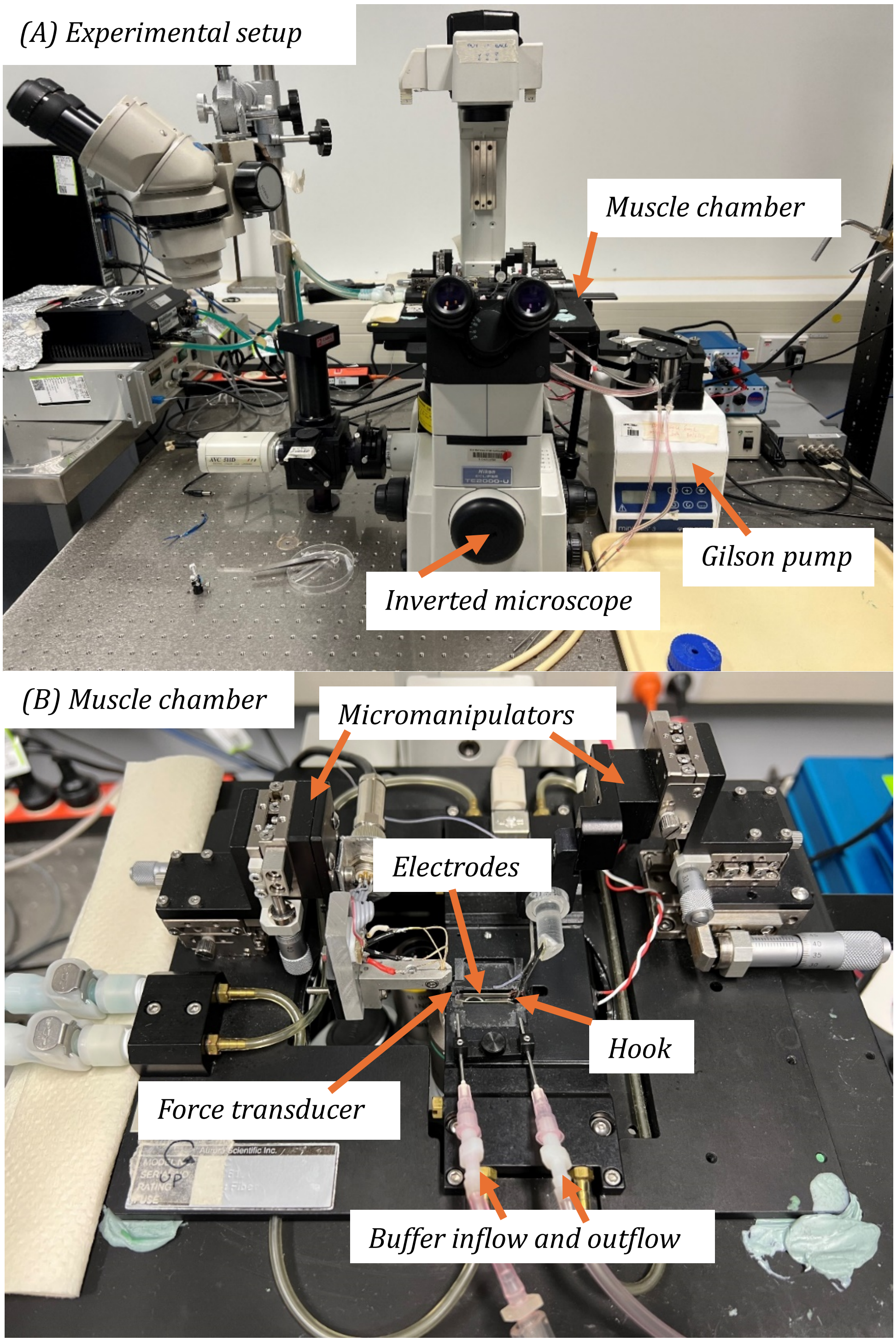

Figure S1p. Experimental setup for measurement of trabeculae contractile force.

(A) shows the inverted microscope, with the muscle chamber on the microscope stage. A Gilson pump circulated oxygenated KH buffer to superfuse the trabecula mounted in the muscle chamber. (B) shows a close-up view of the muscle chamber (Aurora Scientific, Canada) mounted above the objective on the stage of the inverted microscope. Each trabecula was positioned in the muscle chamber between two electrodes, attached at one end to a hook connected to a force transducer. The inflow and outflow ports continuously maintained the circulation of KH buffer in the muscle chamber.

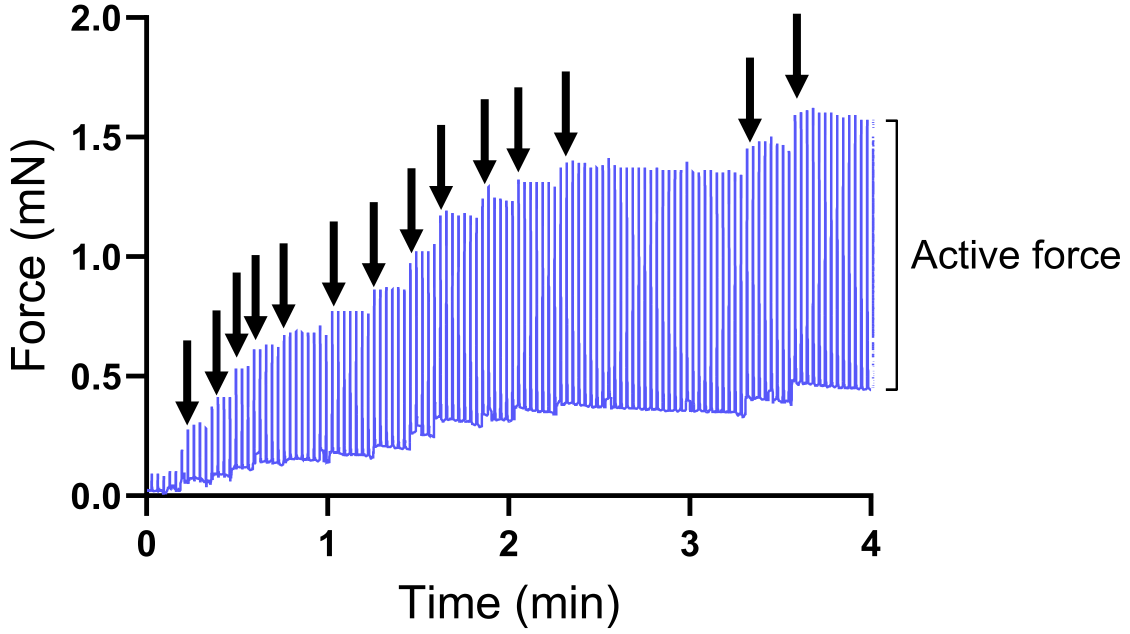

Figure S1q. **Stretching of the trabeculae to the optimal length.**

A representative trace showing the force response to increasing trabecula length. The trabecula was incrementally lengthened (arrows) from slack length until the active force produced no longer increased in response to an increase in length. Passive force and peak force increased with each increase in length. The length of the trabecula where the maximal active force (i.e. peak force minus passive force) was measured was defined as "optimal length" (L_o).

Functional Data Analysis

The contractile force produced by the trabeculae was measured by a force transducer as voltage signals using an instrument interface (AD Instruments, New Zealand) and recorded using the software LabChart (AD Instruments, New Zealand). Prior to experimentation, calibration of the force transducer was carried out by recording the force transducer voltage in response to weights of known mass hung from the force transducer hook. A calibration curve was then graphed, and a calibration factor was determined for the conversion of the force transducer voltage to force in mN within LabChart software.

For each trabecula, the contractile force was converted to stress (in units of mN mm^{-2}) by normalising to trabecula cross-sectional area. This was estimated from the trabecula diameter measured using the inverted microscope (Nikon TE2000-U, Nikon Instruments, Japan) at low magnification during experimentation, assuming each trabecula had a cylindrical shape. Figure S1r S1-18 shows a LabChart output measured from a representative trabecula.

Ten consecutive twitches were selected from the recordings and analysed using the "peak analysis" plugin in LabChart. Figure S1s shows the peak analysis settings used to determine the contractile parameters measured from the twitches. Figure S1t show all the contractile parameters measured from a representative twitch, including peak developed stress, diastolic stress (passive stress), active stress, time to peak stress, and time to 50% twitch relaxation. The contractile data were averaged over the ten twitches and exported for statistical analysis.

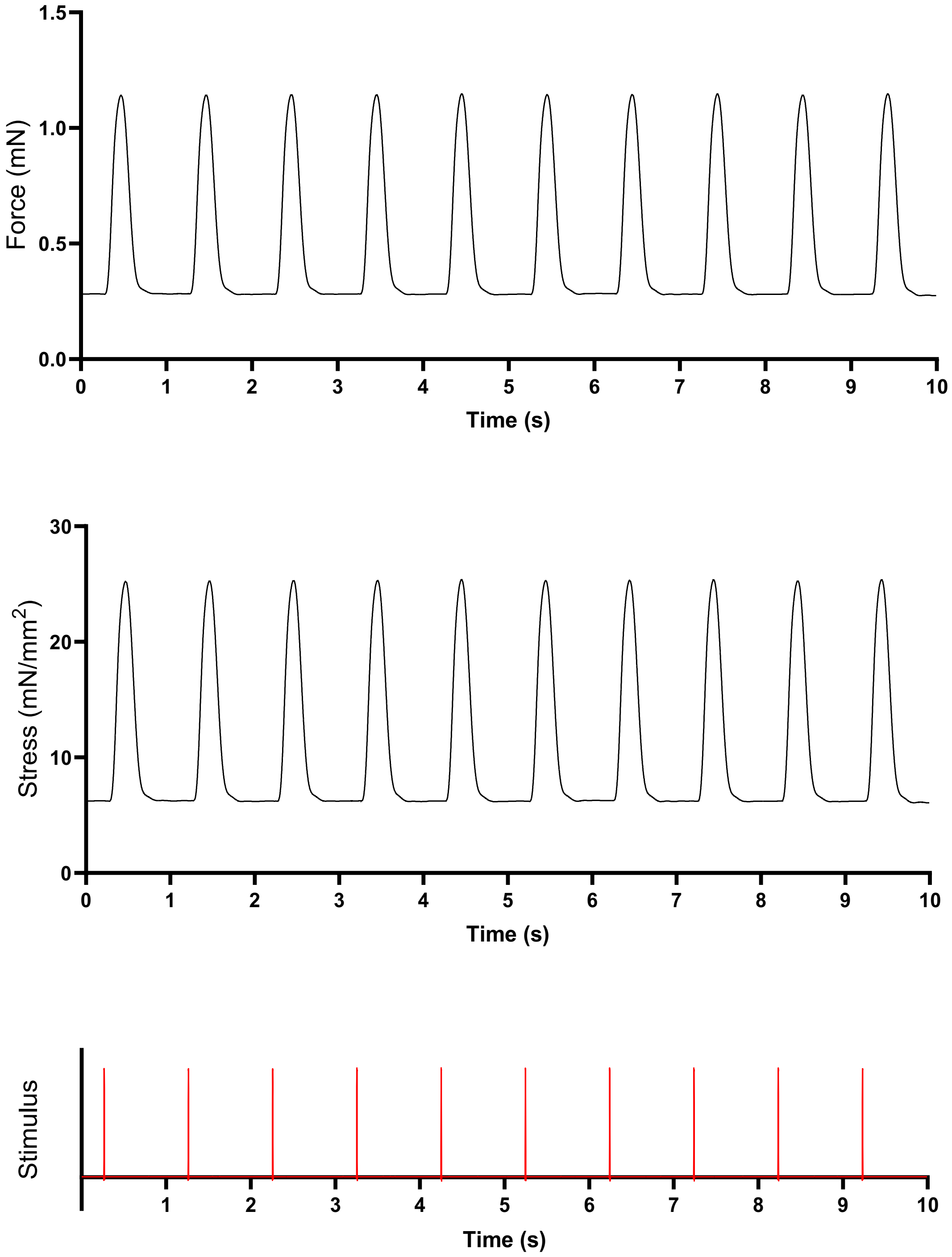

Figure S1r. **Example of a LabChart output.**

This figure shows three variables measured in LabChart software from a representative trabecula at 1 Hz stimulation. The three traces are aligned in time. The top trace shows the isometric force measured when the trabecula contracted at optimal length. The second trace shows the force produced converted to stress by normalising to the cross-sectional area of the trabecula. The bottom trace shows the stimulus (red) delivered at 1 Hz.

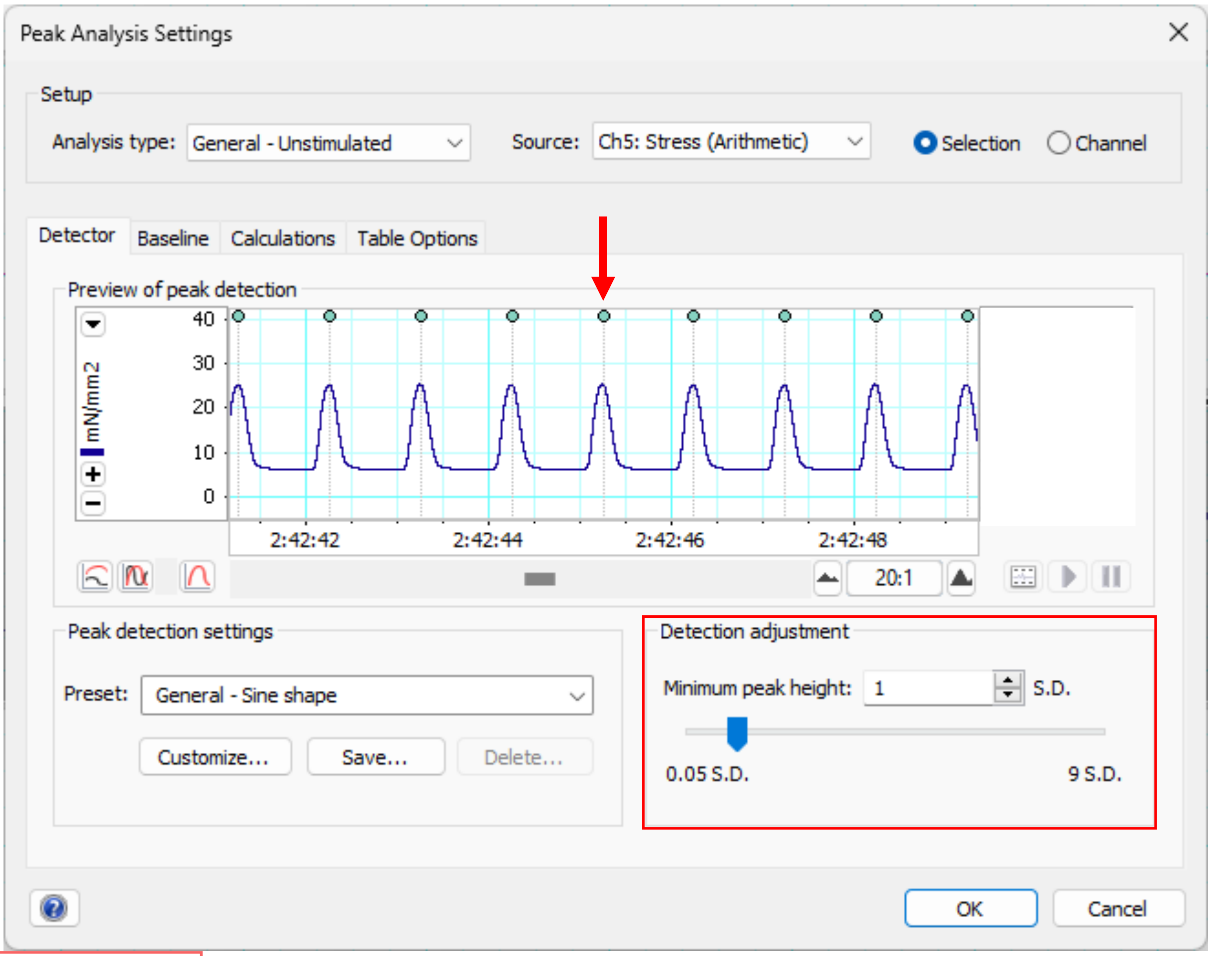

Figure S1s **LabChart peak analysis settings.**

Figures show the LabChart peak analysis settings used to detect and analyse the twitches from 10s. A representative section of steady-state data is shown in Figure S1r. The minimum peak height was adjusted accordingly (red rectangle) until all the twitches were correctly detected with green markers placed on top (red arrow).

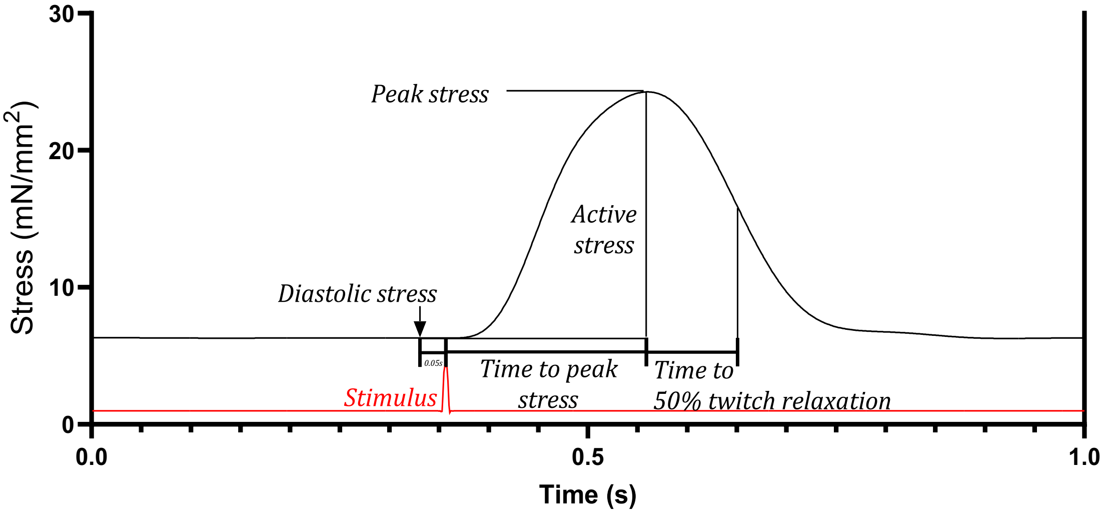

Figure S1t. Peak analysis of a twitch response.

The figure shows the measurements made using LabChart peak analysis of a representative twitch response (black line) stimulated at 1 Hz stimulation frequency (red line). Peak-developed stress is the stress value measured from the peak of the twitch. Diastolic stress is the baseline passive stress measured 0.05 s before stimulation. Active stress is the difference between the peak developed stress and the baseline stress. Time to peak stress is the time taken for the trabecula to develop peak stress from the stimulus. Time to 50% twitch relaxation is the time from peak stress to 50% of the peak stress.

References

Chan, H. Y., Keung, W., Li, R. A., Miller, A. L., & Webb, S. E. (2015). Morphometric Analysis of Human Embryonic Stem Cell-Derived Ventricular Cardiomyocytes: Determining the Maturation State of a Population by Quantifying Parameters in Individual Cells. *Stem Cells Int*, *2015*, 586908. https://doi.org/10.1155/2015/586908

Cooley, J. W., & Tukey, J. W. (1965). An algorithm for the machine calculation of complex Fourier series. *Mathematics of computation*, *19*(90), 297-301.

Dulbecco, R., Allen, R., Okada, S., & Bowman, M. (1983). Functional changes of intermediate filaments in fibroblastic cells revealed by a monoclonal antibody. *Proc Natl Acad Sci U S A*, *80*(7), 1915-1918. https://doi.org/10.1073/pnas.80.7.1915

Feret, L. (1930). *La grosseur des grains des matières pulvérulentes*. Eidgen. Materialprüfungsanstalt ad Eidgen. Technischen Hochschule.

Hillsley, A., Santoso, M. S., Engels, S. M., Halwachs, K. N., Contreras, L. M., & Rosales, A. M. (2022). A strategy to quantify myofibroblast activation on a continuous spectrum. *Scientific reports*, *12*(1), 12239. https://doi.org/10.1038/s41598-022-16158-7

Jones, T. L. M., Kaur, S., Kang, N., Ruygrok, P. N., & Ward, M.-L. (2023). Impaired calcium handling mechanisms in atrial trabeculae of diabetic patients. *Physiological Reports*, *11*(3), e15599. https://doi.org/10.14814/phy2.15599

Kapur, J. N., Sahoo, P. K., & Wong, A. K. C. (1985). A new method for gray-level picture thresholding using the entropy of the histogram. *Computer Vision, Graphics, and Image Processing*, *29*(3), 273-285. https://doi.org/10.1016/0734-189X(85)90125-2

Krebs, H. A., & Henseleit, K. (1932). Untersuchungen über die Harnstoffbildung im Tierkörper. *Klinische Wochenschrift*, *11*, 757-759.

Legland, D., Arganda-Carreras, I., & Andrey, P. (2016). MorphoLibJ: integrated library and plugins for mathematical morphology with ImageJ. *Bioinformatics*, *32*(22), 3532-3534. https://doi.org/10.1093/bioinformatics/btw413

Litviňuková, M., Talavera-López, C., Maatz, H., Reichart, D., Worth, C. L., Lindberg, E. L., Kanda, M., Polanski, K., Heinig, M., Lee, M., Nadelmann, E. R., Roberts, K., Tuck, L., Fasouli, E. S., DeLaughter, D. M., McDonough, B., Wakimoto, H., Gorham, J. M., Samari, S.,…Teichmann, S. A. (2020). Cells of the adult human heart. *Nature*, *588*(7838), 466-472. https://doi.org/10.1038/s41586-020-2797-4

Meyer, F., & Beucher, S. (1990). Morphological segmentation. *Journal of visual communication and image representation*, *1*(1), 21-46. https://doi.org/10.1016/1047-3203(90)90014-M

Mulieri, L. A., Hasenfuss, G., Ittleman, F., Blanchard, E. M., & Alpert, N. R. (1989). Protection of human left ventricular myocardium from cutting injury with 2,3-butanedione monoxime. *Circulation Research*, *65*(5), 1441-1449. https://doi.org/10.1161/01.RES.65.5.1441

Neerad, P., Sumit, M., Ashish, S., & Madhuri, J. (2011, 10-12 Feb. 2011). Adaptive local thresholding for detection of nuclei in diversity stained cytology images. 2011 International Conference on Communications and Signal Processing,

Saito, T., & Toriwaki, J.-I. (1994). New algorithms for euclidean distance transformation of an n-dimensional digitized picture with applications. *Pattern Recognition*, *27*(11), 1551-1565. https://doi.org/10.1016/0031-3203(94)90133-3

Sellin, L. C., & McArdle, J. J. (1994). Multiple Effects of 2,3-Butanedione Monoxime. *Pharmacology & Toxicology*, *74*(4-5), 305-313. https://doi.org/10.1111/j.1600-0773.1994.tb01365.x

Wiggins, J. R., Reiser, J., Fitzpatrick, D. F., & Bergey, J. L. (1980). Inotropic actions of diacetyl monoxime in cat ventricular muscle. *J Pharmacol Exp Ther*, *212*(2), 217-224.

References
