# Supplementary material for: Myocardial composition and contractile function of right atrial trabeculae from type 2 diabetic and nondiabetic male patients
Source: Physiol Rep. 2025 Aug 11;13(15):e70509. doi: 10.14814/phy2.70509 (PMC12339417; doi:10.14814/phy2.70509)
Supplement: Supplementary file 2 — Figure S2. [file PHY2-13-e70509-s001.docx]

Results


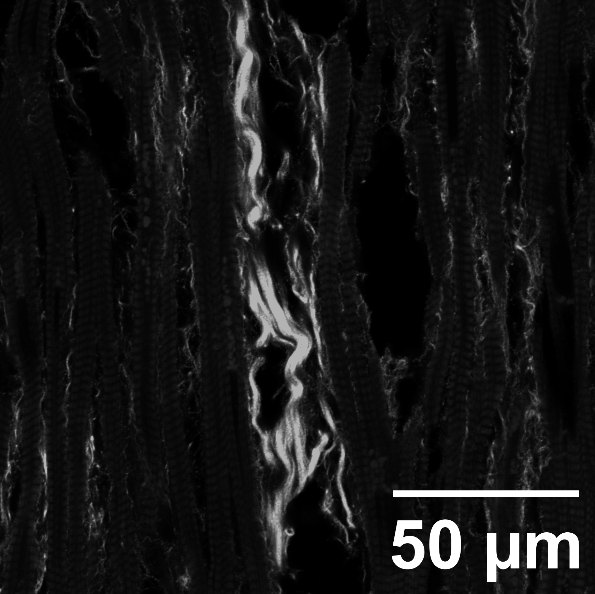

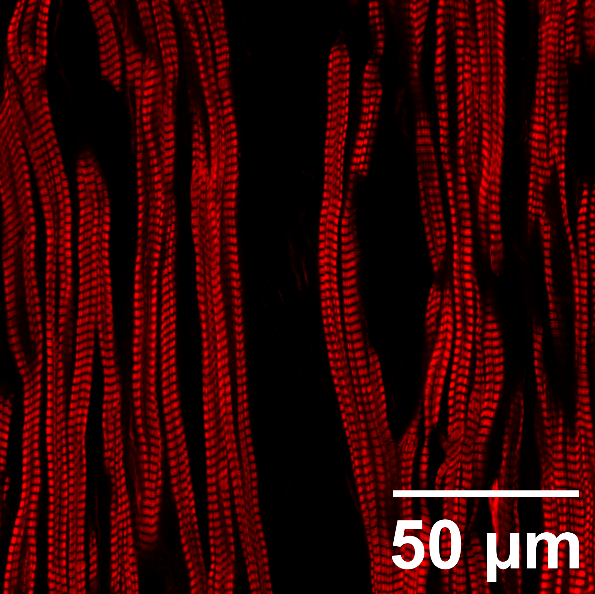

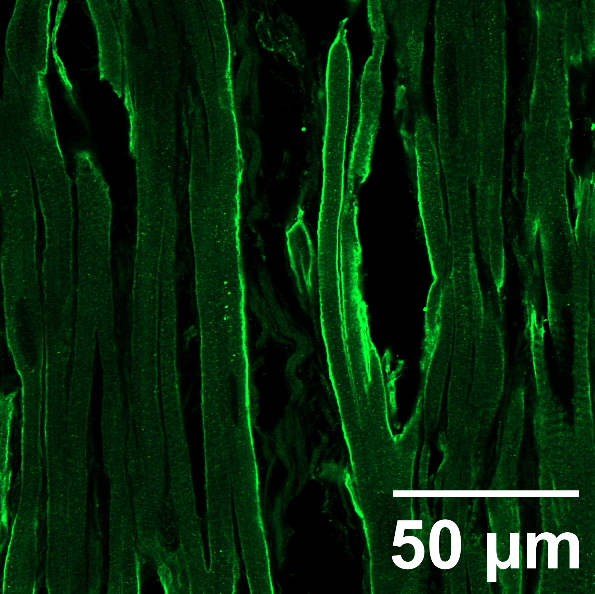

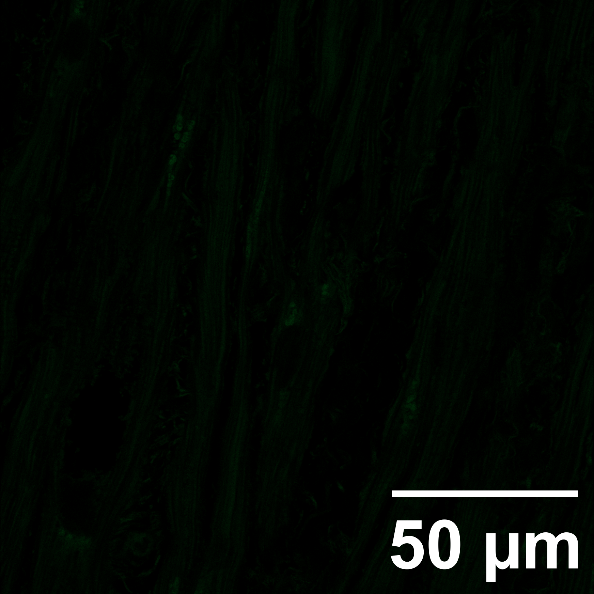

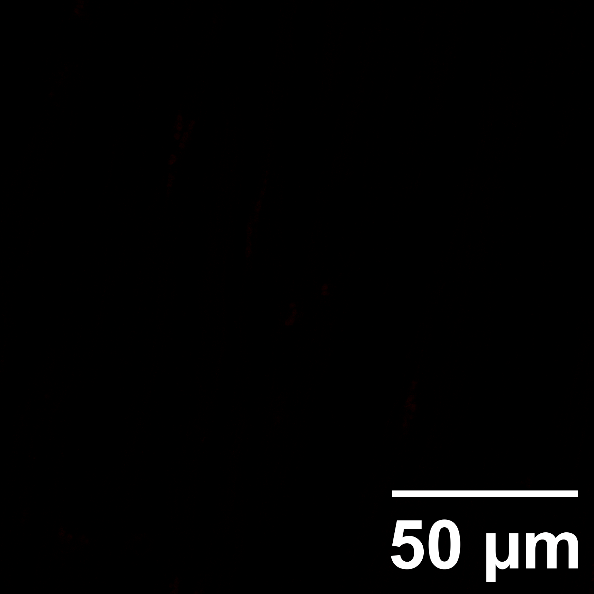

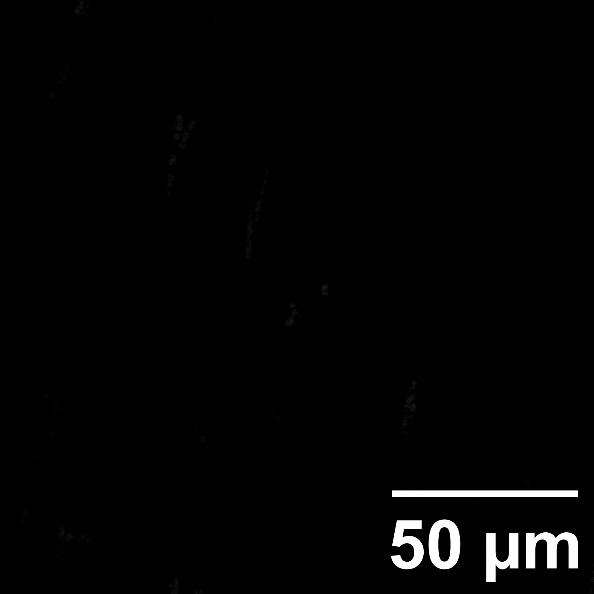

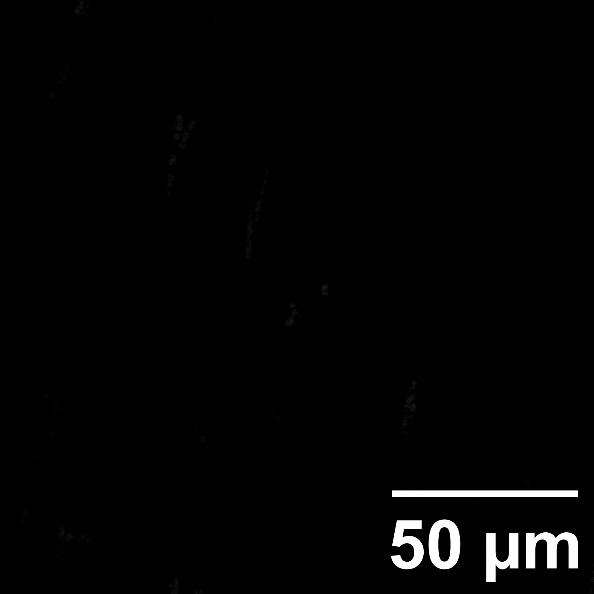

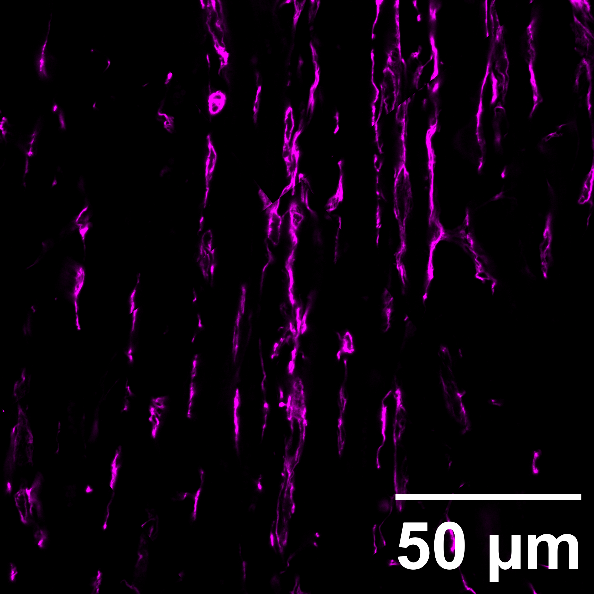


Type I collagen

Myofilament

(F-actin)

Type III collagen

Vimentin

No primary control

Negative control

No primary control

Negative control

Figure S2-1. Antibody validation.

Confocal images show examples of right atrial appendage (RAA) wall tissue labelled with the myofilament-marker phalloidin (red), type I collagen antibody (green), and type III collagen antibody (grey). Images in the top row demonstrate the positive labelling of the targeted proteins. Images in the bottom row demonstrate the no primary antibody controls and negative controls.

- Non-diabetic (n = 5)
- Diabetic (n = 5)

**Figure S2-2. Comparison of relative cell composition from trabeculae transverse sections.**

Panel A shows the cellular composition of right atrial appendage (RAA) trabeculae obtained from ND and T2D patient samples. Panel B illustrates the occurrence of fibroblasts and cardiomyocytes in the myocardial cell population. Data shown in panel B are presented as mean ± SEM with significance determined by nested t-tests. **p* ≤ 0.05 indicates a significant difference between groups.


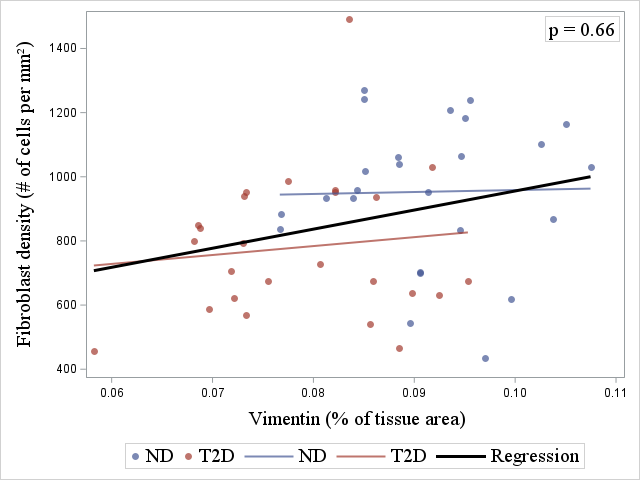


**Figure S2-3. Relationship of fibroblast tissue density with vimentin content in right atrial appendage (RAA) trabeculae.**

The relative abundance of vimentin in the RAA tissue was plotted against fibroblast tissue density. Data points and regression lines from the ND group are shown in blue, and the T2D group are shown in red. The regression lines of the two groups combined are shown in black. The relationship of the two variables in each plot was examined using mixed model analysis. *p* ≤ 0.05 indicates a statistically significant relationship between the variables for the combined groups.

- Non-diabetic (n = 4)
- Diabetic (n = 4)

Figure S2-4. Correlation of time to 50% twitch relaxation with the relative abundance of collagen in the right atrial appendage (RAA) trabeculae.

Time to 50% twitch relaxation (ms) recorded from 4 ND (black) and 4 T2D RAA trabeculae (red) at 2 Hz stimulation frequencies is plotted against the relative abundance of collagen in trabeculae myocardium in panel (A) and endocardium in panel (B). The significance of correlation (*p* ≤ 0.05) was determined by two-tailed tests, and Pearson’s correlation coefficient (r) was calculated and displayed.
